# Mechanochemical synthesis of bis-benzoquinonylmethanes promoted by sulfonic acid-functionalized chitosan

**DOI:** 10.1039/d6ra00136j

**Published:** 2026-03-24

**Authors:** Iva Souza de Jesus, Juliana Baptista de Pontes, Daniel Tadeu Gomes Gonzaga, Fernando de Carvalho da Silva, Vitor Francisco Ferreira

**Affiliations:** a Laboratório de Inovação Em Química e Tecnologia Farmacêutica, Faculdade de Farmácia, Universidade Federal Fluminense Niterói RJ 24241-000 Brazil ivasouza.quimica@gmail.com vitorferreira@id.uff.br; b Laboratório de Síntese Orgânica Aplicada, Instituto de Química, Universidade Federal Fluminense Niterói RJ 24020-141 Brazil; c Instituto Biomédico, Universidade Do Estado Do Rio de Janeiro Rio de Janeiro RJ 23070-200 Brazil

## Abstract

A sustainable mechanochemical method for synthesizing functionalized bis-lawsones from 2-hydroxynaphthalene-1,4-dione and aldehydes under solvent-free conditions is described. Sulfonated chitosan acts as both a biodegradable grinding auxiliary and a solid acid promoter, enabling high yields, broad tolerance to aryl-substituted aldehydes, and recyclability over multiple cycles with minimal loss of activity. The protocol is readily scalable without compromising efficiency.

## Abstract 2

Mechanochemical synthesis of bis-benzoquinonylmethanes using a recyclable sulfonic acid-functionalized chitosan as a promoter under solvent-free conditions, affording high yields with broad substrate scope and a sustainable, scalable protocol..

## Graphical abstract

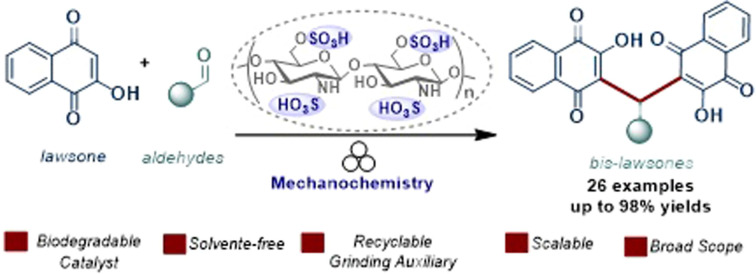

## Introduction

**Scheme 1:**
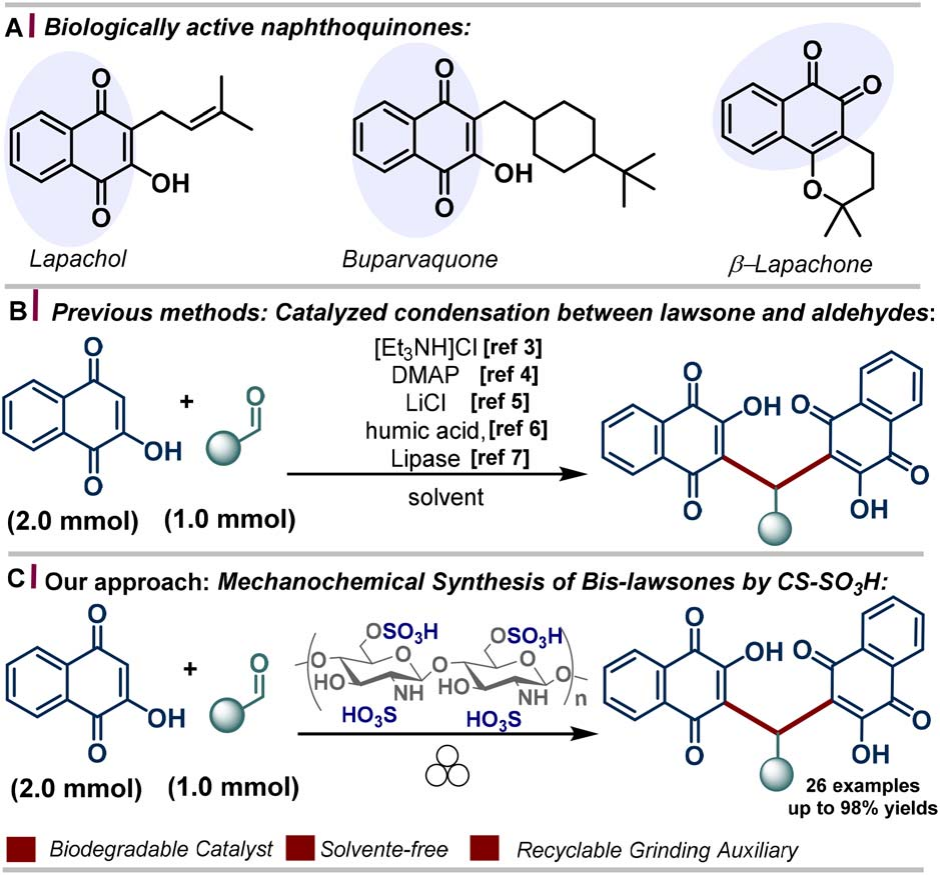
(A) Biologically active quinones. (B) Previous methods for the synthesis of bis-lawsones. (C) This study: mechanochemical synthesis of arylmethylene-bislawsones (3) promoted by CS-SO_3_H.

Quinone-based molecules are a noteworthy class of organic compounds due to their broad biological activities (Scheme 1A), valuable industrial applications, and utility as intermediates in heterocycle synthesis.^1^ These properties have generated substantial interest among synthetic chemists and pharmacologists, motivating the development of sustainable strategies for their synthesis and functionalization.

Among the established strategies for the synthesis of 3,3′-(arylmethylene)bis(2-hydroxynaphthalene-1,4-diones), the pseudo three-component reaction between 2-hydroxy-1,4-naphthoquinone (HNQ) and aldehydes represents a straightforward way for accessing this class of compound. Since this synthesis is based on an inherently slow reaction, several catalysts have been employed to facilitate this reaction (Scheme 1B).^2–10^ Moreover, non-conventional techniques, such as microwave and ultrasound irradiation, have been demonstrated to exert a promotional effect on these reactions.^11^

Despite substantial methodological advancements, the majority of existing protocols suffer from several disadvantages, such as long reaction times, difficult product isolation, high temperatures, low-yielding products, and the employment of toxic organic solvents. Consequently, the development of an operationally simple, efficient, eco-friendly, and energy-saving protocol for the one-pot assembly of biologically important compounds, exemplified by arylmethylene-bislawsones (3), continues to be of significant scientific interest.

Mechanochemical synthesis has emerged as a practical and sustainable alternative to traditional solution-phase methodologies.^12^ These approaches effectively eliminate the need for solvents (sometimes toxic) and elevated temperatures by harnessing the mechanical energy generated by impact forces within the reaction vessel. The continuous development of specialized equipment suitable for small-scale reactions and easy scale-up has expanded the applicability of mechanosynthesis to solvent-free reactions and even to processes involving gas-phase reagents.^13^ Moreover, advances in the elucidation of the mechanisms involved have contributed to the identification of intermediates formed during these reactions.^14^

Recent developments in heterogeneous catalysis have significantly broadened the synthetic toolbox for accessing structurally complex and highly functionalized molecules. In this context, polymer-supported catalysts have attracted growing attention in recent years due to their operational simplicity, ease of product isolation, and advantageous economic and environmental benign.^15–18^ Among these, chitosan (CS) stands out as a promising support material due to its natural origin, biocompatibility, and biodegradability. Its structure, rich in amino and hydroxyl groups, offers multiple reactive sites for chemical modification and the anchoring or stabilization of catalytic species.^19–24^ A notable functionalization strategy involves sulfonation, yielding sulfonic acid-functionalized chitosan (CS-SO_3_H), which has demonstrated efficacy as a solid acid catalyst in various organic transformations.^25–30^ In certain cases, sulfonated chitosan exhibits superior catalytic performance compared to the unmodified form. This enhanced activity is generally attributed to improvements in surface area, a greater density of accessible acidic sites, and increased thermal stability, making the functionalized biopolymer particularly suitable for reactions under elevated temperatures.^31^

We herein report the mechanochemical synthesis of functionalized bis-lawsones (3a–z) *via* the reaction between lawsone (2-hydroxynaphthalene-1,4-dione) (1) and a arylaldehydes (2). The transformation is carried out under solvent-free conditions, employing sulfonated chitosan as a biodegradable, readily available, and recyclable material that acts both as a grinding auxiliary and a solid acid reaction promoter. This protocol enables the efficient and environmentally benign construction of the target compounds, aligning with the principles of green chemistry (Scheme 1C).

## Results and discussion

### Synthesis and characterization of CS-SO_3_H

**Scheme 2:**
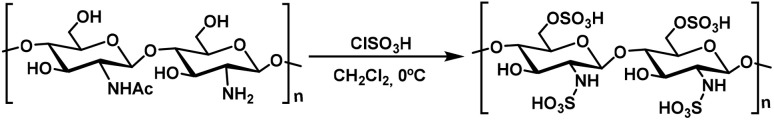
CS-SO_3_H preparation.

Chitosan-SO_3_H (CS-SO_3_H) was prepared according to a previously described protocol.^29^ Chlorosulfonic acid (2 mL) was added dropwise to a magnetically stirred suspension of chitosan (1.00 g) in anhydrous dichloromethane (10 mL) maintained at 0 °C over the course of 1 hour. After the addition was complete, the reaction mixture was allowed to stir at ambient temperature for an additional 2 hours to ensure full elimination of HCl. The solid was then collected by filtration and thoroughly washed with methanol until the filtrate reached neutral pH. The material was subsequently dried at room temperature, affording chitosan-SO_3_H as a white solid (Scheme 2).

For a comprehensive characterization of CS-SO_3_H and verification of its structural, thermal, and elemental features, a combination of complementary analytical techniques was employed.^29^ Fourier Transform Infrared (FT-IR) spectroscopy was applied to confirm the successful introduction of sulfonic groups onto the chitosan framework. Thermogravimetric Analysis was performed to assess the thermal stability, determine the organic fraction, and evaluate the efficiency of component incorporation throughout the synthesis process. X-ray Diffraction (XRD) analysis was conducted to evaluate the crystalline structure of the catalyst and ensure its phase purity and structural integrity during synthesis. Field Emission Scanning Electron Microscopy was used to observe the morphology and particle size distribution, while Energy Dispersive X-ray Spectroscopy provided information on the elemental composition and sulfur content at the catalyst surface.

**Fig. 1:**
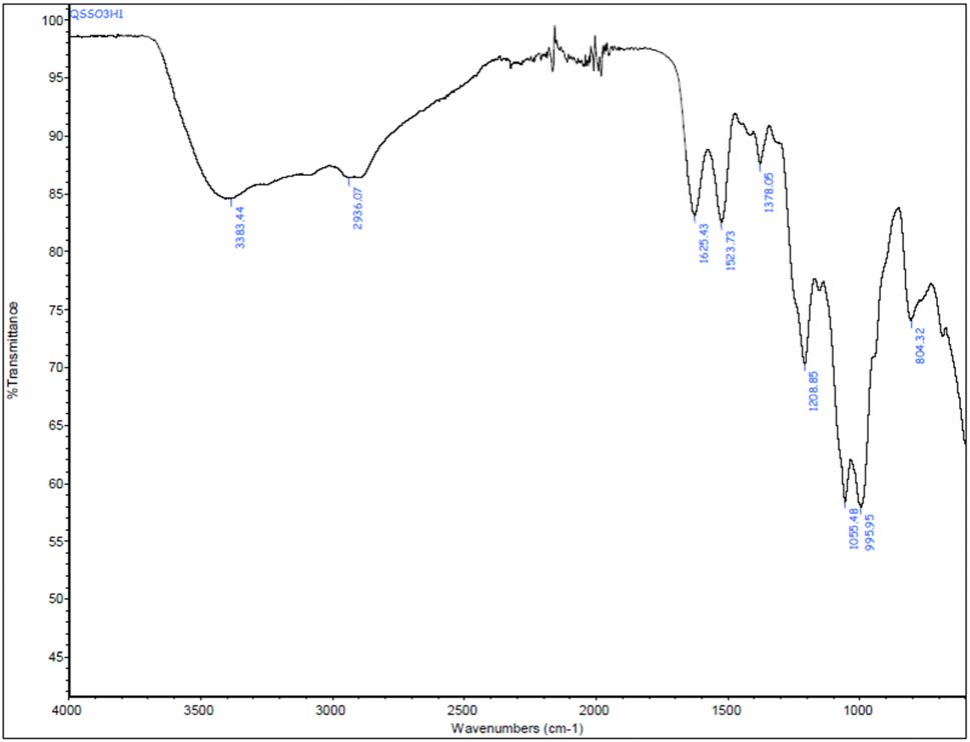
FT-IR spectrum of CS-SO_3_H.

The FT-IR spectrum confirmed the presence of distinct functional groups in CS-SO_3_H. Based on previous studies and the present data, the characteristic bands observed at 1208 cm^−1^ and 1055 cm^−1^ correspond to the S=O stretching vibrations of the –SO_3_H group in –O–SO_3_H and NH–SO_3_H, respectively. In addition, the peak at 804 cm^−1^ is attributed to the stretching vibration of the S–N bond in –HN–SO_3_H (Fig. 1).

**Fig. 2:**
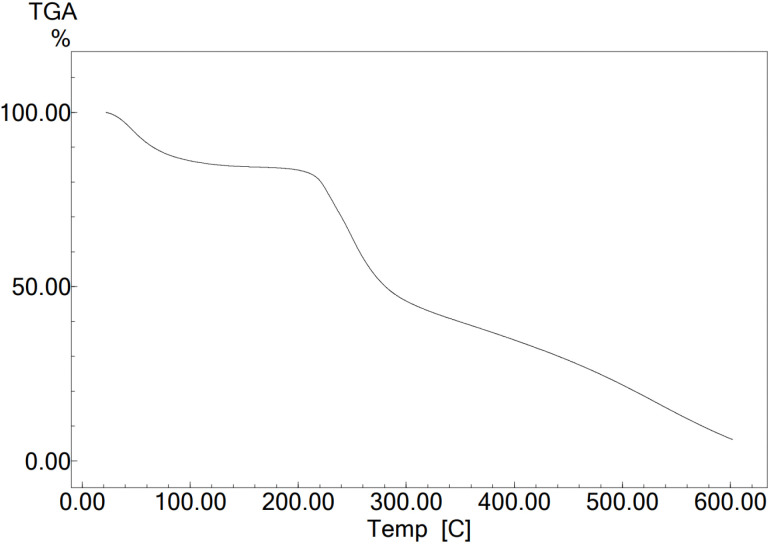
TGA curve of CS-SO_3_H.

The thermal stability of the synthesized catalyst (CS-SO_3_H) was assessed by thermogravimetric analysis (TGA) over the temperature range of 50–500 °C. The first weight loss (approximately 5%) at around 90 °C corresponds to the removal of residual solvent and other small molecules. The second major weight loss (approximately 20–75%) occurring between 250 and 300 °C is associated with the degradation of the chitosan polysaccharide backbone and the SO_3_H groups (Fig. 2).

**Fig. 3:**
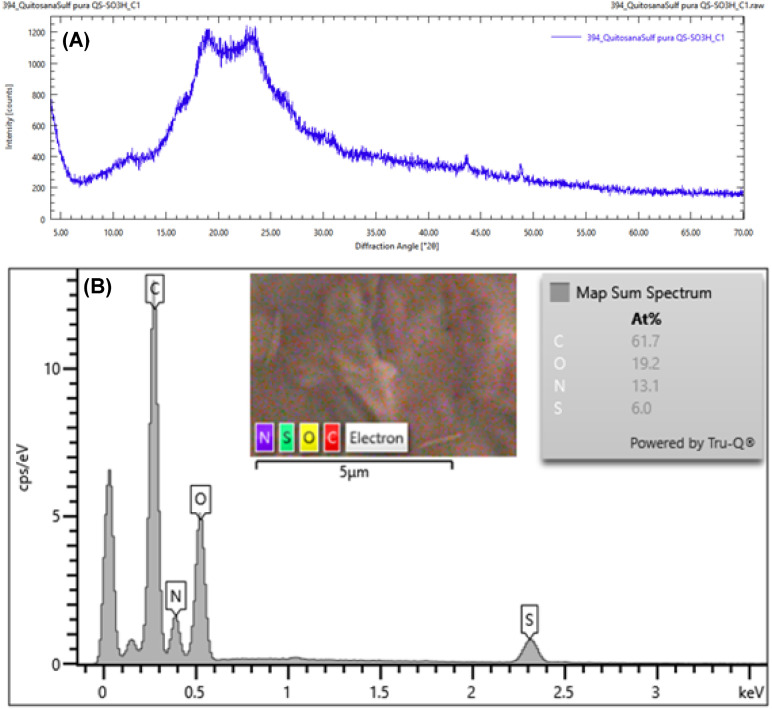
(A) XRD pattern of CS-SO_3_H; (B) EDS spectra of the CS-SO_3_H.

The XRD pattern of CS-SO_3_H exhibits a broad diffraction peak centered between 15° and 25° (2*θ*), characteristic of the semicrystalline nature of chitosan. After sulfonation, the peak becomes broader and less intense, indicating increased amorphization due to disruption of intermolecular interactions and hydrogen bonding. This structural change confirms that the introduction of –SO_3_H groups reduces crystallinity and enhances the amorphous character of the material (Fig. 3A). EDS analysis revealed the elemental composition of the material, confirming the presence of carbon, oxygen, nitrogen, and sulfur with weight percentages of 61.7%, 19.2%, 13.1%, and 6.0%, respectively. These results verify the successful incorporation of –SO_3_H groups onto the chitosan backbone (Fig. 3B). Furthermore, the surface morphology, particle features, and size distribution of CS-SO_3_H were examined using FE-SEM (Fig. 3). The observations showed a uniform fibrous surface with visible voids and cracks, which are expected to serve as active sites for the catalytic reaction.

### Evaluation of the activity of CS-SO_3_H in the mechanochemical synthesis of arylmethylene-bislawsones

The study was initiated using lawsone (1) and benzaldehyde (2a) as model substrates to evaluate the influence of different parameters on the reaction outcome. The reaction between 1 (2.0 equiv.) and 2a (1.0 equiv.) was carried out in the presence of 100 mg of CS-SO_3_H as catalyst, yielding the desired product (3a) in 54% after 2 h of grinding at 3000–4000 oscillations per minute, using 10 stainless steel balls (5 mm × 16) (Table 1, entry 1). During this process, the formation of a paste was observed (see Fig. S5 in the SI), reducing the efficiency of collisions during the milling process,^32^ and consequently lowering product yields. To overcome this limitation, a grinding aid was added. Thus, 800 mg of silica gel was added, as a grinding auxiliary solid, allowing the reaction to proceed efficiently (Table 1, entry 2). To enhance sustainability and allow catalyst reuse, acidic chitosan was evaluated in a dual role as grinding auxiliary and catalyst, providing 3a in 92% yield after 1 h under identical conditions (Table 1, entry 3). When the reaction was conducted using 10 mol% of *p*-toluenesulfonic acid, only 66% of 3a was obtained (Table 1, entry 4). In contrast, when the amount of *p*-TSA was increased to 1 equiv., the product 3a was obtained in 87% yield (Table 1, entry 5). Another advantage of CS-SO_3_H over conventional *p*-TSA is its straightforward and clean separation from the reaction medium, in addition to its recyclability. The reaction was subsequently evaluated using unmodified chitosan (low molecular weight); however, no formation of the target product was observed (Table 1, entry 6), underscoring the crucial role of the SO_3_H functionalities in aldehyde activation and stabilization of the reaction intermediates. Reducing the CS-SO_3_H amount (Table 1, entries 7 and 8) or the number of grinding balls (Table 1, entries 9 and 10) decreased efficiency. Control experiments confirmed that both CS-SO_3_H and the grinding balls were essential for the reaction (Table 1, entries 11 and 12).

**Table 1:** Optimization and control studies^a^

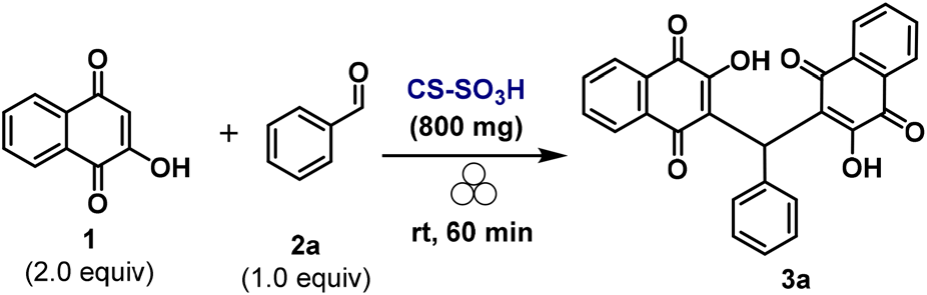		
Entry	Deviations from the optimized condition	Yield 3a^b^ (%)
1	100 mg of CS-S0_3_H	54
2	100 mg of CS-SO_3_H and 800 mg of SiO_2_	70
3	None	92
4	10 mol% of *p*-TSA	66
5	1.0 equiv. of *p*-TSA	87
6	Chitosan	nr
7	500 mg of CS-SO_3_H	81
8	700 mg of CS-S0_3_H	87
9	5 stainless steel grinding balls of 5 mm	Trace
10	8 stainless steel grinding balls of 5 mm	69
11	Without catalyst	nr
12	Without grinding balls	nr
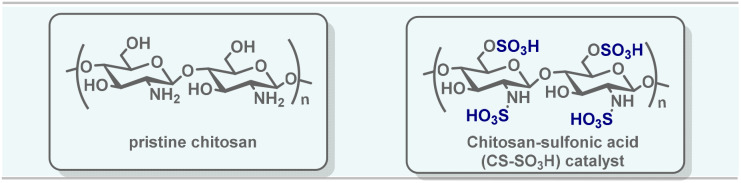		

a Unless otherwise noted, all reactions were carried out with 1 (1.00 mmol), 2a (2.00 mmol), and CS-SO_3_H (800 mg), using 10 stainless steel grinding balls (5 mm diameter × 16) in a Ultra-Turrax Tube Drive (IKA) of 15 mL.

b lsolated yield.

**Scheme 3:**
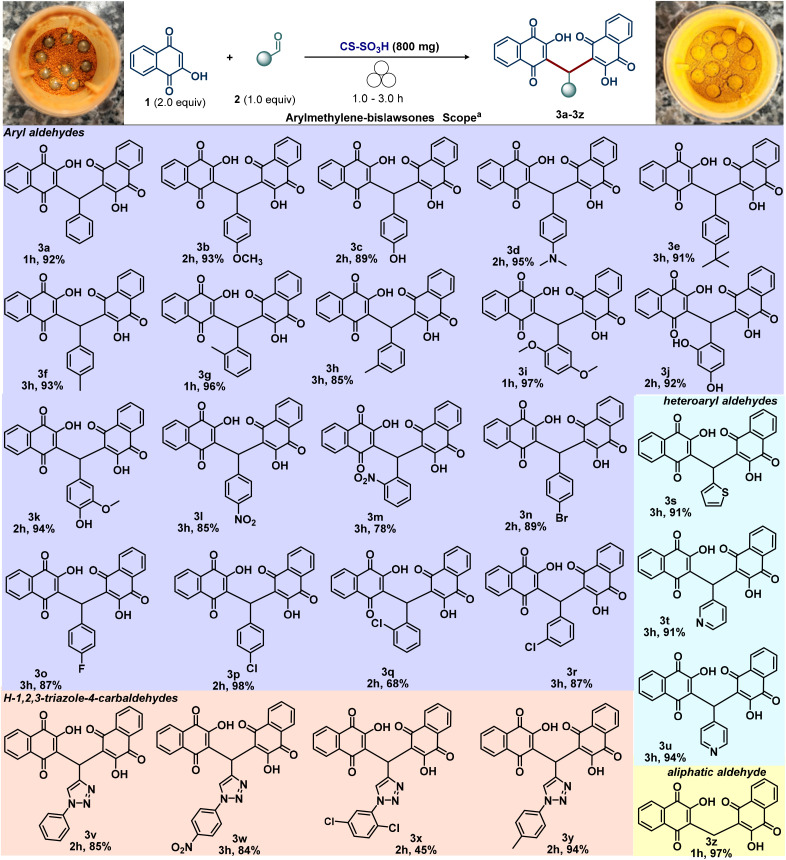
Mechanochemical synthesis of arylmethylene-bislawsones (3) using CS-SO_3_H as both a biodegradable grinding auxiliary and catalyst.

With the optimal reaction conditions established, we then focused our attention on evaluating the scope and limitations of the mechanochemical synthesis of arylmethylene-bislawsones (3) promoted by CS-SO_3_H (Scheme 3). Accordingly, unsubstituted aldehyde (3a) as well as aryl aldehydes bearing electron-donating (3b–3k) or electron-withdrawing (3l–3r) groups successfully underwent the transformation, furnishing the corresponding products in moderate to good yields. Notably, substituents in the *ortho*, *meta*, and *para* positions were all well accommodated under the optimized conditions. In addition, the bromine-containing aryl aldehyde—an important synthon for transition-metal-catalyzed cross-coupling reactions—also reacted efficiently, providing product 3n in good yield. Furthermore, a series of heteroaryl substituents, including pyridyl (3t and 3u) and thienyl (3s), proved to be compatible under the reaction conditions. The corresponding products were obtained in satisfactory yields, demonstrating the good functional group tolerance of the mechanochemical synthesis of bis-benzoquinonylmethanes.

**Fig. 4:**
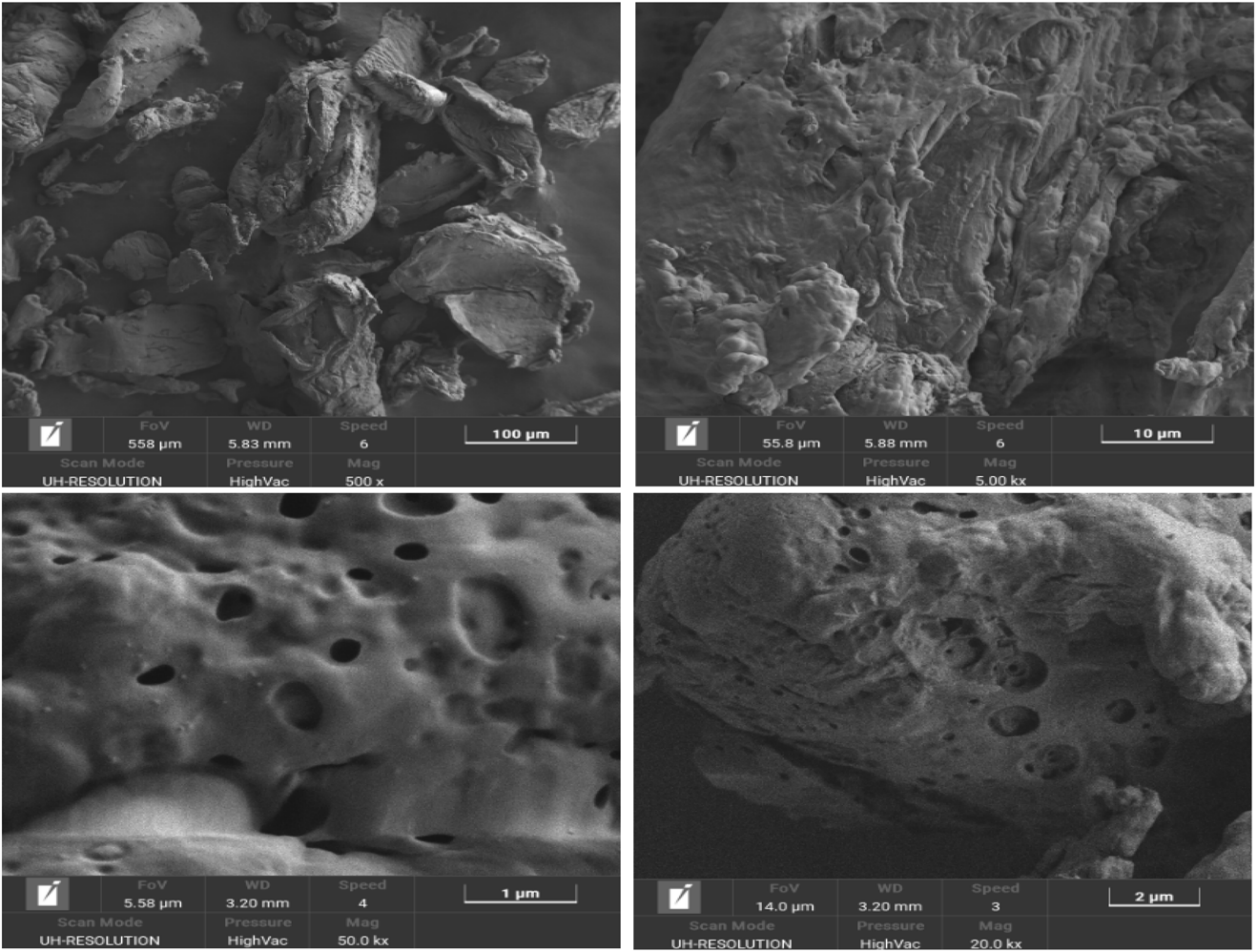
FE-SEM images of CS-SO_3_H.

Evaluation of aliphatic aldehydes, including paraformaldehyde, phenylacetaldehyde, citral, and citronellal, revealed that only paraformaldehyde underwent successful conversion to product 3z in high yield (94%). We assume that aliphatic aldehydes exhibited inferior performance due to the absence of electronic conjugation and stabilization that enhance the reactivity of aromatic aldehydes. Moreover, steric effects and higher volatility under milling further decreased their efficiency, with only paraformaldehyde affording high yields. Other aldehydes, such as furfural, glucose, and reactive ketones like isatin, were also investigated; however, no product formation was observed. Densely functionalized triazole derivatives are very important chemical architectures, also presenting physiological and pharmacological properties.^33^ Therefore, the development of straightforward protocol to achieve this important scaffold is highly desirable. Gratifyingly, employing our protocol afforded the 3,3′-((1-aryl-1*H*-1,2,3-triazol-4-yl)methylene)bis(2-hydroxynaphthalene-1,4-diones) 3v–3y, highlighting the robustness of this method (Scheme 3 and Fig. 4).

**Fig. 5:**
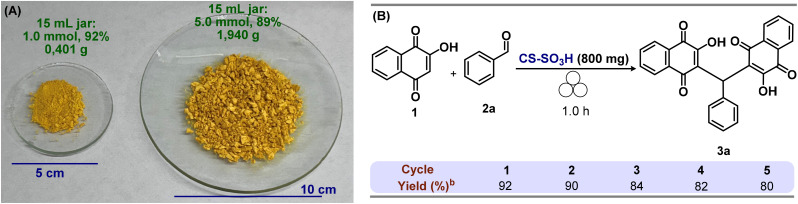
(A) Mechanochemical synthesis scale-up of arylmethylene-bislawsone 3a (B) evaluation of CS-SO_3_H recycling.

To demonstrate the robustness and efficiency of the method, the standard reaction using model substrate 1a was performed on a 5.0 mmol scale. The desired product 3a was obtained in 89% yield, with no observable loss in reactivity, highlighting the potential of this protocol as a sustainable and environmentally benign synthetic approach (Fig. 5A). Furthermore, the recyclability of CS-SO_3_H was further investigated, as the ability to reuse a heterogeneous catalyst over multiple cycles represents a major advantage and a key criterion for its practical applicability in sustainable synthetic methodologies.

After separation from the reaction mixture by filtration, CS-SO_3_H was washed with hot ethanol and air-dried. The recovered material was then reused in the model reaction for five consecutive cycles. A slight decrease in product yield was observed with each reuse (run 1: 92%; run 2: 90%; run 3: 84%; run 4: 82%; run 5: 80%), highlighting the catalyst's notable recoverability and demonstrating its potential for recyclability with only a moderate loss in catalytic activity (Fig. 5B).

**Fig. 6:**
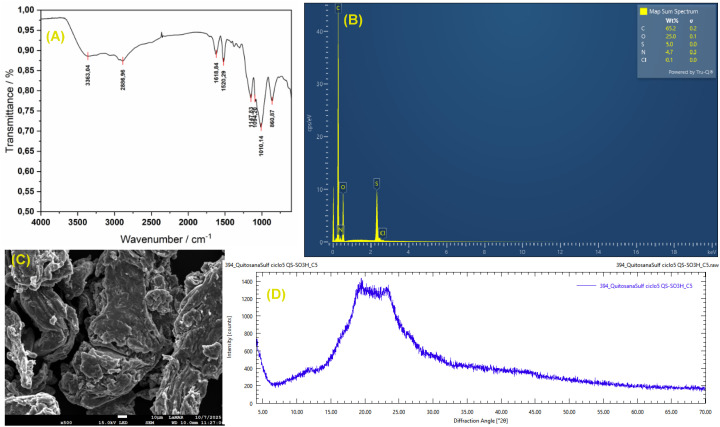
FT-IR spectrum (A), EDS analysis (B), FE-SEM image (C), and XRD pattern (D) of CS-SO_3_H recycled.

To assess the structural and functional stability of CS-SO_3_H under solvent-free mechanochemical conditions, FT-IR, EDS, FESEM, and XRD analyses were performed on the material before and after the reaction. The consistent spectral and morphological features confirm that CS-SO_3_H remains stable throughout the process (Fig. 6).

By examining the FT-IR spectrum of the recycled material, it was observed that the structure of CS-SO_3_H remained intact after the reaction (Fig. 6A). EDS analysis further confirmed that the elemental composition of the CS-SO_3_H was preserved after reuse (Fig. 6B). FE-SEM images revealed that the overall morphology, particle size, and surface uniformity of CS-SO_3_H were virtually unchanged, with no evidence of particle aggregation, surface collapse, or structural degradation—demonstrating the strong structural integrity of the CS-SO_3_H under both chemical and mechanical stress (Fig. 6C). Moreover, comparison of the XRD patterns before and after the reaction showed no significant changes or shifts in the position or intensity of the diffraction peaks (Fig. 6D). Collectively, these results confirm the high crystallographic and structural stability of CS-SO_3_H, and, together with its excellent recyclability, underscore its effectiveness as a dual-function material serving both promoter and grinding-assistant roles in mechanochemical synthesis.

This work advances beyond previous methods by combining the dual functionality of sulfonated chitosan as both solid acid and grinding auxiliary under solvent-free conditions, leading to significantly improved reaction efficiency. Compared with recent protocols employing humic acid, sulfamic acid, or camphor sulfonic acid catalysts, the present method delivers higher yields (up to 97%) in markedly shorter reaction times (1–3 h *versus* 6–12 h) without the need for organic solvents or high temperatures. Moreover, the CS-SO_3_H can be reused for at least five consecutive cycles with only minor loss of activity, highlighting its superior sustainability and environmental performance relative to conventional approaches.

**Scheme 4:**
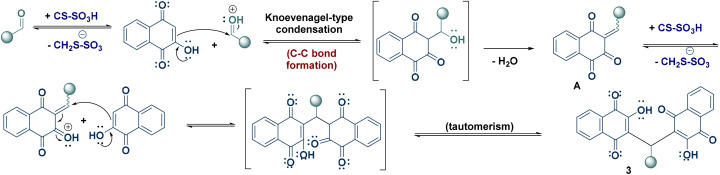
Proposed mechanism for the synthesis of arylmethylene-bislawsones (3).

Based on the obtained results and previous literature reports,^10^ a plausible reaction mechanism is proposed, highlighting the catalytic role of CS-SO_3_H (Scheme 4). In the first step, quinone methide intermediate A is formed *via* nucleophilic attack of lawsone on the protonated aldehyde 2, followed by elimination of water. Subsequently, intermediate A reacts with a second molecule of lawsone to generate species B, which then undergoes tautomerization to yield the desired product 3.

## Experimental

### General experimental information

All chemicals were purchased and used without further purification. Anhydrous solvents were either purchased or dried employing standard drying agents and freshly distilled before use. Reactions were monitored by Thin-layer chromatography (TLC) (Silica gel 60 F254, Merck KGaA, Darmstadt, Germany). Flash column chromatography was performed using Silica Gel 60 M (40–63 µm, Machery Nagel GmbH & Co., Düren, Germany). Melting points were obtained on a Fisatom 430D apparatus and uncorrected. Infrared spectra were recorded on an FT-IR Thermo Nicolet IS-50 equipment operated in the ATR mode (32 scans) (resolution 4 cm^−1^). ^1^H and ^13^C NMR spectra were acquired on a Bruker Advance NEO spectrometer operating at 500 MHz, employing a direct broadband probe at 125 MHz in CDCl_3_ or DMSO-*d*_6_ at 25 °C. Chemical shifts (*δ*) are reported in parts per million relatives to residual solvent signals, and coupling constants (*J*) are reported in hertz. Multiplicities are described as brs = broad signal, s = singlet, d = doublet, t = triplet, q = quartet, dd = doublet of doublets, dt = doublet of triplets, and m = multiplet. APPI-Q-TOFMS measurements were obtained on a mass spectrometer equipped with an automatic syringe pump for sample injection.

Chitosan-SO_3_H (CS-SO_3_H) was synthesized following a literature process^30*a*^ (characterization data and FT-IR spectra, Thermogravimetric Analysis (TGA), Field Emission Scanning Electron Microscopy (FE-SEM), Energy Dispersive X-ray Spectroscopy (EDS), and X-ray Diffraction (XRD) analysis, are provided in the SI).

### General procedure

3,3′-(Arylmethylene)bis(2-hydroxynaphthalene-1,4-diones) synthesis general procedure on a 1.0 mmol scale (3a–z). In a 15 mL BMT-20-S tube (IKA Ultra-Turrax Tube Drive) containing 10 stainless steel balls (5 mm × 16), the appropriate aldehyde (1.0 mmol, 1.0 equiv.) and 2-hydroxy-1,4-naphthoquinone (2.0 mmol, 2.0 equiv.) were milled for 5 min at 300–4000 oscillations per minute. Subsequently, chitosan-SO_3_H (0.80 g) was added to the mixture and milling was continued for the time indicated in each case under the same oscillation conditions. The reaction progress was monitored by TLC. After grinding, the crude mixture was transferred to a becker and extracted with 30 mL of ethanol. The solid chitosan-SO_3_H was filtered off, washed with hot ethanol, and stored for reuse. The solvent was evaporated under reduced pressure, and the product was recrystallized from ethanol. 3,3′-(Arylmethylene)bis(2-hydroxynaphthalene-1,4-diones) 3a–z were determined by ^1^H, and ^13^C NMR spectroscopy (characterization data and ^1^H, and ^13^C NMR spectra are provided in the SI). General procedure on a 5 mmol scale. The aforementioned protocol was followed using lawsone (1,74 g, 10.0 mmol), benzaldehyde 2a (05 102 mL, 5.0 mmol), and chitosan-SO_3_H (0.800 g). The solid chitosan-SO_3_H was filtered off, washed with hot ethanol, and stored for reuse. The solvent was evaporated under reduced pressure, and the product was recrystallized from ethanol providing compound 3a as a yellow solid (1940 mg, 89%).

3,3′-(Phenylmethylene)bis(2-hydroxynaphthalene-1,4-dione) (3a): ^7^ reaction time: 1 hour (401 mg, 92% yield), yellow solid, purified by recrystallization in ethanol, mp: 206.2–209.0 °C. ^1^H NMR (DMSO-*d*_6_, 500 MHz) *δ* 7.99 (dd, *J* 7.5, 1.5 Hz, 2H), 7.93 (dd, *J* 7.5, 1.5 Hz, 2H), 7.85–7.74 (m, 4H), 7.26–7.16 (m, 4H), 7.15–7.09 (m, 1H), 6.03 (s, 1H). FT-IR (ATR, *v*max/cm^−1^): 3328, 3060, 1641, 1590, 1494, 1299, 1264, 1039, 721.

3,3′-((4-Methoxyphenyl)methylene)bis(2-hydroxynaphthalene-1,4-dione) (3b):^7^ reaction time: 2 hours (433 mg, 93% yield), yellow solid, purified by recrystallization in ethanol, mp: 226.8–230.3 °C. ^1^H NMR (DMSO-*d*_6_, 500 MHz) *δ* 7.99–7.96 (m, 2H), 7.94–7.91 (m, 2H), 7.79 (ddd, *J* 16.1, 7.5, 1.5 Hz, 4H), 7.17–7.08 (m, 2H), 6.75 (d, *J* 8.7 Hz, 2H), 5.97 (s, 1H), 3.70 (s, 3H). FT-IR (ATR, *v*_max_/cm^−1^): 3395, 3239, 1634, 1458, 1333, 1042, 967, 821, 718.

3,3′-((4-Hydroxyphenyl)methylene)bis(2-hydroxynaphthalene-1,4-dione) (3c):^8*c*^ reaction time: 02 hours (401 mg, 89% yield), brown solid, purified by recrystallization in ethanol, mp: 207.2–209.0 °C. ^1^H NMR (DMSO-*d*_6_, 500 MHz) *δ* 7.99–7.95 (m, 2H), 7.93–7.90 (m, 2H), 7.85–7.72 (m, 5H), 7.05–6.96 (m, 2H), 6.62–6.54 (m, 2H), 5.92 (s, 1H). FT-IR (ATR, *v*_max_/cm^−1^): 3388, 3277, 1654, 1450, 1042, 1007, 829, 724, 689.

3,3′-((4-(Dimethylamino)phenyl)methylene)bis(2-hydroxynaphthalene-1,4-dione) (3d):^7^ reaction time: 02 hours (456 mg, 95% yield), yellow solid, purified by recrystallization in ethanol, mp: 154.0–156.8 °C. ^1^H NMR (DMSO-*d*_6_, 500 MHz) *δ* 7.94 (ddd, *J* 8.8, 7.6, 1.4 Hz, 4H), 7.83–7.70 (m, 4H), 7.12 (d, *J* 8.2 Hz, 2H), 6.15 (s, 1H), 2.93 (s, 6H). FT-IR (ATR, *v*_max_/cm^−1^): 3537, 2363, 1670, 1460, 1214, 1133, 1050, 912, 838.

3,3′-((4-(Tert-butyl)phenyl)methylene)bis(2-hydroxynaphthalene-1,4-dione) (3e): previously unreported compound reaction time: 03 hours (449 mg, 91% yield), yellow solid, purified by recrystallization in ethanol, mp: 226.7–227.7 °C. ^1^H NMR (DMSO-*d*_6_, 500 MHz) *δ* 8.02–7.70 (m, 8H), 7.33–7.02 (m, 4H), 6.00 (s, 1H), 1.25 (s, 9H). ^13^C{^1^H} NMR (DMSO-*d*_6,_ 125 MHz) *δ* 31.7, 34.4, 37.6, 123.7, 124.8, 126.0, 126.5, 128.3, 130.3, 132.7, 133.5, 135.1, 138.1, 147.9, 156.6, 181.6, 184.0. FT-IR (ATR, *v*_max_/cm^−1^): 3239, 2957, 1639, 1345, 1277, 1050, 904, 830, 791. HRMS (ESI): *m*/*z* calc. for C_31_H_24_NaO_6_ [M + Na]^+^ 515.1465, found 515.1458.

3,3′-(*p*-Tolylmethylene)bis(2-hydroxynaphthalene-1,4-dione) (3f):^8*b*^ reaction time: 03 hours (421 mg, 93% yield), yellow solid, purified by recrystallization in ethanol, mp: 179.1–182.0 °C. ^1^H NMR (DMSO-*d*_6_, 500 MHz) *δ* 7.98 (dd, *J* 7.4, 1.5 Hz, 2H), 7.92 (dd, *J* 7.6, 1.4 Hz, 2H), 7.85–7.74 (m, 4H), 7.11 (d, *J* 7.9 Hz, 2H), 6.99 (d, *J* 7.9 Hz, 2H), 5.99 (s, 1H), 2.24 (s, 3H). FT-IR (ATR, *v*_max_/cm^−1^): 3335, 3027, 1653, 1626, 1459, 1301, 897, 866, 722.

3,3′-(*o*-Tolylmethylene)bis(2-hydroxynaphthalene-1,4-dione) (3g):^3*c*^ reaction time: 01 hours (431 mg, 96% yield), yellow solid, purified by recrystallization in ethanol, mp: 220.6–223.7 °C. ^1^H NMR (DMSO-*d*_6_, 500 MHz) *δ* 8.02–7.96 (m, 2H), 7.95–7.90 (m, 2H), 7.85–7.74 (m, 4H), 7.17 (dd, *J* 7.4, 1.6 Hz, 1H), 7.11–6.98 (m, 3H), 6.01 (s, 1H), 2.21 (s, 3H). FT-IR (ATR, *v*_max_/cm^−1^): 3290, 3247, 1671, 1630, 1459, 1336, 1270, 779, 724.

3,3′-(*m*-Tolylmethylene)bis(2-hydroxynaphthalene-1,4-dione) (3h):^8*c*^ reaction time: 3 hours (382 mg, 85% yield), yellow solid, purified by recrystallization in ethanol, mp: 221.2–223.7 °C. ^1^H NMR (DMSO-*d*_6_, 500 MHz) *δ* 8.01–7.96 (m, 2H), 7.95–7.90 (m, 2H), 7.80 (ddd, *J* 15.8, 7.5, 1.5 Hz, 4H), 7.11–6.98 (m, 3H), 6.95–6.89 (m, 1H), 6.01 (s, 1H), 2.22 (s, 3H). FT-IR (ATR, *v*_max_/cm^−1^): 3342, 2901, 1654, 1628, 1459, 901, 860, 755, 698.

3,3′-((2,5-Dimethoxyphenyl)methylene)bis(2-hydroxynaphthalene-1,4-dione) (3i): previously unreported compound reaction time: 1 hour (481 mg, 97% yield), orange solid, purified by recrystallization in ethanol, mp: 217.8–218.7 °C. ^1^H NMR (DMSO-*d*_6_, 500 MHz) *δ* 8.09 (ddd, *J* 11.8, 7.7, 1.4 Hz, 5H), 7.80–7.58 (m, 7H), 6.74 (s, 3H), 6.29 (s, 1H), 3.71 (d, *J* 9.6 Hz, 6H). ^13^C{^1^H} NMR (DMSO-*d*_6,_ 125 MHz) *δ* 33.6, 55.5, 56.5, 110.3, 111.2, 116.8, 123.8, 126.0, 126.5, 130.2, 130.6, 132.6, 133.5, 135.1, 152.0, 153.3, 156.2, 181.5, 183.7; FT-IR (ATR, *v*_max_/cm^−1^): 3206, 3129, 3038, 2835, 1652, 1444, 1303, 920, 861. HRMS (ESI): *m*/*z* calc. for C_29_H_20_NaO_8_ [M + Na]^+^ 519,4608, found 519.1046.

3,3′-((2,4-Dihydroxyphenyl)methylene)bis(2-hydroxynaphthalene-1,4-dione) (3j): previously unreported compound reaction time: 2 hours (159 mg, 92% yield), orange solid, purified by recrystallization in ethanol, mp: 251.9–255.7 °C. ^1^H NMR (DMSO-*d*_6_, 500 MHz) *δ* 8.04–7.92 (m, 4H), 7.87–7.75 (m, 5H), 6.93 (d, *J* 8.4 Hz, 1H), 6.65–6.49 (m, 2H), 6.18 (s, 1H), 5.65 (s, 1H); ^13^C{^1^H} NMR (DMSO-*d*_6,_ 125 MHz) *δ* 103.3, 111.5, 113.6, 125.8, 126.1, 126.2, 126.4, 129.7, 130.3, 130.7, 131.0, 131.6, 132.0, 132.4133.6134.3134.9, 135.0, 135.2, 149.8, 157.9, 178.3, 181.7, 183.4; FT-IR (ATR, *v*_max_/cm^−1^): 3374, 1672, 1642, 1457, 1308, 1210, 904, 867, 797.

3,3′-((4-Hydroxy-3-methoxyphenyl)methylene)bis(2-hydroxynaphthalene-1,4-dione) (3k):^7^ reaction time: 2 hours (433 mg, 94% yield), yellow solid, purified by recrystallization in ethanol, mp: 224.3–228.6 °C. ^1^H NMR (DMSO-*d*_6_, 500 MHz) *δ* 8.03–7.90 (m, 5H), 7.85–7.74 (m, 5H), 6.80 (s, 1H), 6.59 (d, *J* 2.1 Hz, 2H), 5.94 (s, 1H), 3.64 (s, 3H); FT-IR (ATR, *v*_max_/cm^−1^): 3326, 1637, 1460, 1245, 1202, 1042, 903, 861, 800.

3,3′-((4-Nitrophenyl)methylene)bis(2-hydroxynaphthalene-1,4-dione) (3l):^8*b*^ reaction time: 3 hours (412 mg, 85% yield), orange solid, purified by recrystallization in ethanol, mp: 182.4–185.6 °C. ^1^H NMR (DMSO-*d*_6_, 500 MHz) *δ* 8.12–7.88 (m, 6H), 7.87–7.75 (m, 4H), 7.58–7.51 (m, 2H), 6.09 (s, 1H). FT-IR (ATR, *v*_max_/cm^−1^): 3324, 1644, 1459, 1348, 1294, 1052, 1017, 858, 820.

3,3′-((2-Nitrophenyl)methylene)bis(2-hydroxynaphthalene-1,4-dione) (3m):^8*c*^ reaction time: 3 hours (378 mg, 78% yield), yellow solid, purified by recrystallization in ethanol, mp: 212.9–214.0 °C. ^1^H NMR (DMSO-*d*_6_, 500 MHz) *δ* 8.01–7.90 (m, 6H), 7.88–7.74 (m, 8H), 7.57–7.50 (m, 3H), 7.47–7.40 (m, 2H), 6.42 (s, 1H). FT-IR (ATR, *v*_max_/cm^−1^): 3353, 3083, 1643, 1607, 1578, 1460, 1297, 1249, 902.

3,3′-((4-Bromophenyl)methylene)bis(2-hydroxynaphthalene-1,4-dione) (3n):^7^ reaction time: 2 hours (458 mg, 89% yield), yellow solid, purified by recrystallization in ethanol, mp: 200.6–202.9 °C. ^1^H NMR (DMSO-*d*_6_, 500 MHz) *δ* 8.03–7.88 (m, 4H), 7.86–7.73 (m, 4H), 7.36 (d, *J* 8.5 Hz, 2H), 7.20 (d, *J* 8.5 Hz, 2H), 5.97 (s, 1H); FT-IR (ATR, *v*_max_/cm^−1^): 3335, 1653, 1641, 1459, 1277, 1209, 1300, 897, 826.

3,3′-((4-Fluorophenyl)methylene)bis(2-hydroxynaphthalene-1,4-dione) (3o):^8*c*^ reaction time: 3 hours (396 mg, 87% yield), yellow solid, purified by recrystallization in ethanol, mp: 165.0–168.3 °C. ^1^H NMR (DMSO-*d*_6_, 500 MHz) *δ* 8.03–7.88 (m, 4H), 7.86–7.73 (m, 4H), 7.27 (d, *J* 3.6 Hz, 2H), 7.00 (t, *J* 8.9 Hz, 2H), 5.98 (s, 1H). FT-IR (ATR, *v*_max_/cm^−1^): 3294, 1638, 1459, 1274, 1213, 1007, 905, 797, 721.

3,3′-((4-Chlorophenyl)methylene)bis(2-hydroxynaphthalene-1,4-dione) (3p):^7^ reaction time: 2 hours (461 mg, 98% yield), yellow solid, purified by recrystallization in ethanol, mp: 171.1–175.5 °C. ^1^H NMR (DMSO-*d*_6_, 500 MHz) *δ* 8.07–7.89 (m, 4H), 7.88–7.71 (m, 4H), 7.33–7.16 (m, 4H), 5.99 (s, 1H); FT-IR (ATR, *v*_max_/cm^−1^): 3339, 1641, 1590, 1459, 1300, 1277, 898, 808, 722.

3,3′-((2-Chlorophenyl)methylene)bis(2-hydroxynaphthalene-1,4-dione) (3q):^8*c*^ reaction time: 2 hours (320 mg, 68% yield), yellow solid, purified by recrystallization in ethanol, mp: 226.6–230.6 °C. ^1^H NMR (DMSO-*d*_6_, 500 MHz) *δ* 8.00 (dd, *J* 7.4, 1.5 Hz, 2H), 7.93 (dd, *J* 7.7, 1.5 Hz, 2H), 7.87–7.75 (m, 4H), 7.33 (td, *J* 7.7, 2.1 Hz, 2H), 7.25–7.14 (m, 2H), 6.10 (s, 1H); FT-IR (ATR, *v*_max_/cm^−1^): 3410, 3224, 3071, 1663, 1635, 1470, 1299, 873, 820.

3,3′-((3-Chlorophenyl)methylene)bis(2-hydroxynaphthalene-1,4-dione) (3r):^8*c*^ reaction time: 3 hours (412 mg, 87% yield), yellow solid, purified by recrystallization in ethanol, mp: 225.2–228.8 °C. ^1^H NMR (DMSO-*d*_6_, 500 MHz) *δ* 8.02–7.90 (m, 4H), 7.86–7.73 (m, 4H), 7.32–7.12 (m, 4H), 6.00 (s, 1H); FT-IR (ATR, *v*_max_/cm^−1^): 3345, 1643, 1627, 1297, 1265, 907, 816, 790, 691.

3,3′-(Thiophen-2-ylmethylene)bis(2-hydroxynaphthalene-1,4-dione) (3s):^33^ reaction time: 3 hours (405 mg, 91% yield), yellow solid, purified by recrystallization in ethanol, mp: 201.2–204.6 °C. ^1^H NMR (DMSO-*d*_6_, 500 MHz) *δ* 7.96 (ddd, *J* 12.0, 7.6, 1.4 Hz, 4H), 7.86–7.74 (m, 4H), 7.25 (dd, *J* 4.9, 1.5 Hz, 1H), 6.89–6.79 (m, 2H), 6.30 (d, *J* 1.1 Hz, 1H); FT-IR (ATR, *v*_max_/cm^−1^): 3254, 1672, 1638, 1459, 1343, 1267, 972, 726, 690.

3,3′-(Pyridin-3-ylmethylene)bis(2-hydroxynaphthalene-1,4-dione) (3t):^4^ reaction time: 3 hours (400 mg, 91% yield), orange solid, purified by recrystallization in ethanol, mp: 230.0–233.2 °C. ^1^H NMR (DMSO-*d*_6_, 500 MHz) *δ* 8.72–8.52 (m, 2H), 8.23 (d, *J* 8.3 Hz, 1H), 8.01–7.89 (m, 4H), 7.84–7.68 (m, 6H), 6.62 (s, 1H); FT-IR (ATR, *v*_max_/cm^−1^): 3440, 3056, 2887, 2455, 1640, 1251, 1080, 998, 917.

3,3′-(Pyridin-4-ylmethylene)bis(2-hydroxynaphthalene-1,4-dione) (3u): previously unreported compound reaction time: 3 hours (411 mg, 94% yield), orange solid, purified by recrystallization in ethanol, mp: 247.0–251.6 °C. ^1^H NMR (DMSO-*d*_6_, 500 MHz) *δ* 8.61 (d, *J* 6.9 Hz, 2H), 8.01–7.89 (m, 4H), 7.84–7.76 (m, 4H), 7.71 (td, *J* 7.5, 1.4 Hz, 2H), 6.78 (s, 1H). ^13^C{^1^H} NMR (DMSO-*d*_6,_ 125 MHz) *δ* 35.4120.5, 125.6, 125.8, 126.3, 131.4, 132.7, 133.4, 134.4, 142.2, 162.9, 164.1, 182.6, 183.2; FT-IR (ATR, *v*_max_/cm^−1^): 3059, 2856, 1671, 1637, 1278, 1059, 912, 874, 801. HRMS (ESI): *m*/*z* calc. for C_26_H_16_NO_6_ [M + H]^+^ 438.0978 found 438.0966.

3,3′-((1-Phenyl-1H-1,2,3-triazol-4-yl)methylene)bis(2-hydroxynaphthalene-1,4-dione) (3v):^34^ reaction time: 2 hours (251 mg, 85% yield), yellow solid, purified by recrystallization in ethanol, mp: 206.8–210.4 °C. ^1^H NMR (DMSO-*d*_6_, 500 MHz) *δ* 8.56 (s, 1H), 8.04–7.93 (m, 4H), 7.89–7.76 (m, 6H), 7.54 (t, *J* 8.0 Hz, 2H), 7.47–7.39 (m, 1H), 6.13 (s, 1H); FT-IR (ATR, *v*_max_/cm^−1^): 3290, 3145, 1649, 1460, 1252, 1047, 1028, 901, 726.

3,3′-((1-(4-Nitrophenyl)-1H-1,2,3-triazol-4-yl)methylene)bis(2-hydroxynaphthalene-1,4-dione) (3w):^34^ reaction time: 3 hours (320 mg, 84% yield), yellow solid, purified by recrystallization in ethanol, mp: 208.9–210.7 °C. ^1^H NMR (DMSO-*d*_6_, 500 MHz) *δ* 8.77 (s, 1H), 8.41 (d, *J* 9.3 Hz, 2H), 8.18 (d, *J* 9.3 Hz, 2H), 8.06–7.93 (m, 4H), 7.90–7.75 (m, 4H), 6.14 (s, 1H); FT-IR (ATR, *v*_max_/cm^−1^): 3348, 3132, 3077, 1680, 1643, 1458, 1306, 1009, 903.

3,3′-((1-(2,5-Dichlorophenyl)-1H-1,2,3-triazol-4-yl)methylene)bis(2-hydroxynaphthalene-1,4-dione) (3x):^34^ reaction time: 2 hours (472 mg, 94% yield), yellow solid, purified by recrystallization in ethanol, mp: 206.6–208.6 °C. ^1^H NMR (DMSO-*d*_6_, 500 MHz) *δ* 10.14 (s, 1H), 9.36 (s, 1H), 8.03–7.99 (m, 3H), 7.94 (dd, *J* 7.3, 1.4 Hz, 2H), 7.86–7.77 (m, 7H), 6.17 (s, 1H). FT-IR (ATR, *v*_max_/cm^−1^): 3139, 1699, 1678, 1633, 1342, 1280, 1043, 808, 725.

3,3′-((1-(*p*-Tolyl)-1H-1,2,3-triazol-4-yl)methylene)bis(2-hydroxynaphthalene-1,4-dione) (3y): previously unreported compound reaction time: 2 hours (490 mg, 94% yield), yellow solid, purified by recrystallization in ethanol, mp: 217.9–220.0 °C. ^1^H NMR (DMSO-*d*_6_, 500 MHz) *δ* 8.59 (d, *J* 0.9 Hz, 1H), 8.03–7.87 (m, 6H), 7.86–7.74 (m, 4H), 7.64–7.58 (m, 2H), 6.11 (d, *J* 0.9 Hz, 1H), 2.07 (s, 3H). ^13^C{^1^H} NMR (DMSO-*d*_6,_ 125 MHz) *δ* 20.9, 30.2, 119.6, 121.2, 122.1, 126.0, 126.5, 130.4, 130.5, 132.7, 133.5, 135.1, 138.0, 148.0, 157.1, 181.7, 183.6; FT-IR (ATR, *v*_max_/cm^−1^): 3329, 3146, 1649, 1588, 1459, 1339, 1046, 899, 817. HRMS (ESI): *m*/*z* calc. for C_30_H_19_N_3_NaO_6_ [M + Na]^+^ 540.1172, found 540.1170.

3,3′-Methylenebis(2-hydroxynaphthalene-1,4-dione) (3z):^6^ reaction time: 1 hour (350 mg, 97% yield), yellow solid, purified by recrystallization in ethanol, mp: 251.5–254.0 °C. ^1^H NMR (DMSO-*d*_6_, 500 MHz) *δ* 7.97 (ddd, *J* 7.6, 6.2, 1.4 Hz, 4H), 7.88–7.71 (m, 4H), 3.76 (s, 2H); FT-IR (ATR, *v*_max_/cm^−1^): 3071, 1678, 1457, 1307, 1262, 1210, 1069, 974, 732.

## Conclusions

In summary, we have developed a simple, efficient, and environmentally benign mechanochemical protocol for the synthesis of functionalized arylmethylene-bislawsones (3) *via* the reaction of lawsone with a broad range of aldehydes. The use of sulfonated chitosan as both a biodegradable grinding auxiliary and a solid acid catalyst proved crucial, providing high yields, short reaction times, and broad functional group tolerance under solvent-free conditions. This approach also enables straightforward catalyst recovery and reuse over multiple cycles with minimal loss of activity, demonstrating its practical applicability. The scalability of the process without compromising efficiency further highlights its potential as a sustainable alternative to conventional solution-based methodologies for the preparation of arylmethylene-bislawsones.

## Author contributions

I. S. J. and J. B. P carried out most of the experiments, prepared the starting materials, and began the method optimization. The manuscript was written by I. S. J., F. C. S., D. T. G. G., and V. F. F. All authors provided manuscript inputs and result discussions.

## Conflicts of interest

There are no conflicts to declare.

## Supplementary Material

RA-016-D6RA00136J-s001

## Data Availability

The data presented in this study are available in the published article and its online supplementary information (SI). Supplementary information: experimental procedures, characterization data, nuclear magnetic resonance spectra, and high-resolution mass spectrometry analyses are provided. See DOI: https://doi.org/10.1039/d6ra00136j.
