# Supplementary material for: Mechanochemical synthesis of bis-benzoquinonylmethanes promoted by sulfonic acid-functionalized chitosan
Source: RSC Adv. 2026 Mar 24;16(18):16229–40. doi: 10.1039/d6ra00136j (PMC13010341; doi:10.1039/d6ra00136j)
Supplement: RA-016-D6RA00136J-s001 [file RA-016-D6RA00136J-s001.pdf]

Supplementary Information (SI) for RSC Advances.
This journal is © The Royal Society of Chemistry 2026

Supporting Information

Mechanochemical Synthesis of Bis-benzoquinonylmethanes Promoted by Sulfonic Acid–Functionalized Chitosan

Iva S. de Jesus,*[a,b] Juliana Baptista de Pontes,[a,b] Daniel T. G. Gonzaga,[a,c] Fernando de C. da Silva,[b] and Vitor Francisco Ferreira*[a]

[a] *Laboratório de Inovação em Química e Tecnologia Farmaceutica, Faculdade de Farmácia, Universidade Federal Fluminense, Niterói, RJ, 24241-000, Brazil.*

[b] *Laboratório de Síntese Orgânica Aplicada, Instituto de Química, Universidade Federal Fluminense, Niterói, RJ, 24020-141, Brazil.*

[c] *Instituto Biomédico, Universidade do Estado do Rio de Janeiro, Rio de Janeiro, RJ, 23070-200, Brazil.*

Corresponding authors: * *E-mail: ivasouza.quimica@gmail.com; vitorferreira@id.uff.br*

1. **General Considerations** **S3**
2. **Synthesis and characterization of Chitosan-SO_3H** **S3**
3. **Characterization of reuse Chitosan-SO_3H** **S8**
4. **Titration procedure** **S9**
5. **Scale-up Experiment** **S10**
6. **General Procedure for 3,3'-(arylmethylene)bis(2- hydroxynaphthalene-1,4-diones)** **S10**
7. **Compound Characterization Data** **S11**
8. **^1H, ^{13}C NMR and HRMS spectra** **S20**

1. General Considerations

All chemicals were purchased and used without further purification. Anhydrous solvents were either purchased or dried employing standard drying agents and freshly distilled before use. Reactions were monitored by Thin-layer chromatography (TLC) (Silica gel 60 F254, Merck KGaA, Darmstadt, Germany) and visualization was carried out by short wavelength UV light (254 nm). Flash column chromatography was performed using Silica Gel 60 M (40−63 μm, Machery Nagel GmbH & Co., Düren, Germany). TGA and FE-SEM, images were taken with Shimadzu TGA-60 Thermal Analyzer and scanning electron microscope (SEM) with field emission gun (Model: JSM 7100F), equipped with EDXS of SDD (Silicon drift detector) and STEM (Scanning Transmission Electron Microscope) detector respectively. X-ray Diffraction (XRD) analysis was conducted using a D2 PHASER diffractometer (Bruker). Melting points were obtained on a Fisatom 430D apparatus and uncorrected. Infrared spectra were recorded on an FT-IR Thermo Nicolet IS-50 apparatus operated in the ATR mode (32 scans) (resolution 4 cm^{-1}). ^{1}H and ^{13}C NMR spectra were acquired on a Bruker Advance NEO spectrometer operating at 500 MHz, employing a direct broadband probe at 125 MHz in $CDCl_3$ or DMSO-d_6 at 25 °C. Chemical shifts (δ) are reported in parts per million relative to the residual solvent signals, and coupling constants (*J*) are reported in hertz. Multiplicities are described as brs = broad signal, s = singlet, d = doublet, t = triplet, q = quartet, dd = doublet of doublets, dt = doublet of triplets, and m = multiplet. APPI-Q-TOFMS measurements were obtained on a mass spectrometer equipped with an automatic syringe pump for sample injection.

2. Chitosan-SO_3H synthesis and characterization

Chitosan-SO_3H (CS-SO_3H) was prepared according to a literature method [30a]. Chlorosulfonic acid (2 mL) was added dropwise at 0 °C for 1 h to a magnetically stirred suspension of chitosan (1.00 g) in dry dichloromethane (10 mL). After complete addition, the mixture was stirred for another 2 h at room temperature until HCl was removed from the reaction vessel. The mixture was then filtered and washed several times with methanol until obtaining neutral pH, followed by drying at room temperature to obtain chitosan-SO_3H as a white solid.

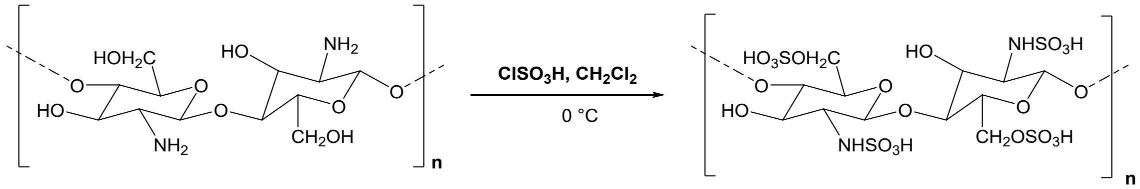

Scheme S1. CS-SO_3H preparation.

Catalyst characterization.

FT-IR catalyst analysis:

The absorption band at 1642 cm^{-1} is due to N-H bending vibration in the chitosan FT-IR spectra. The peaks at 1064 cm^{-1} and 1024 cm^{-1} are due to the stretching vibrations of C-O bonds. Concerning modified chitosan (CS-SO_3H) FT-IR spectra, the characteristic bands at 1205 $cm^{-1}$and 1084 cm^{-1} are due to-S=O stretching bands of -SO_3H in –O-SO_3H and NH-SO_3H groups, while the peak at 790 cm^{-1} is due to the stretching vibration of the S-N bond in -HN-SO_3H (Figure S2).

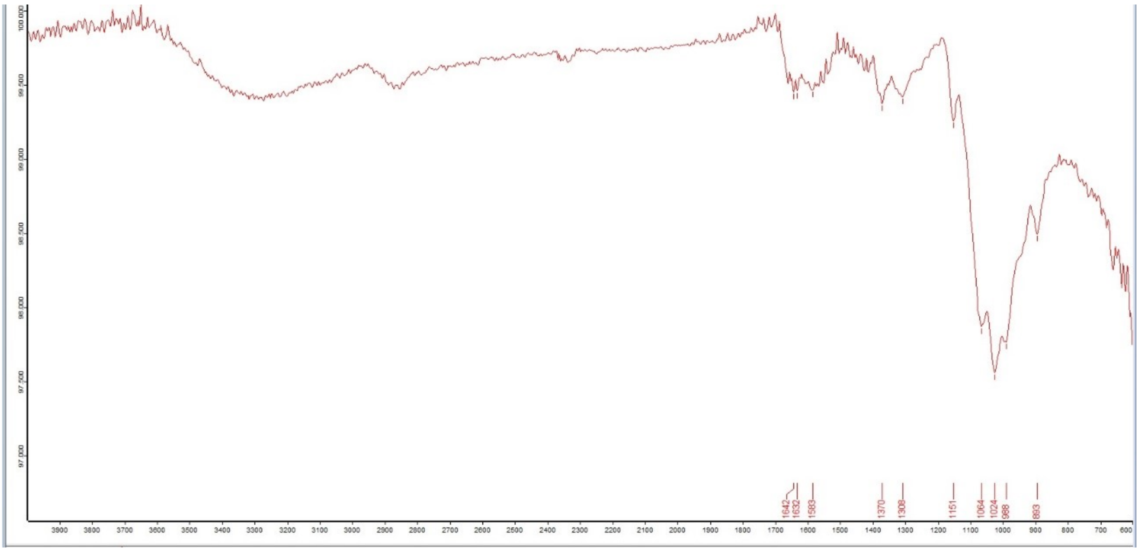

Figure S1: Chitosan FT-IR spectra.

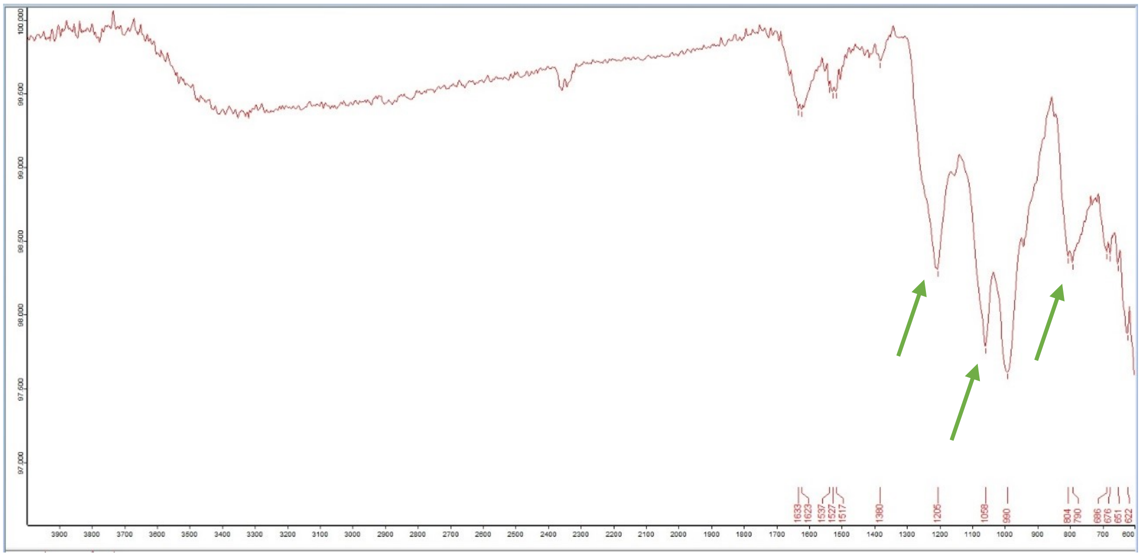

Figure S2: Chitosan-SO_3H FT-IR spectra.

Thermogravimetric Analysis (TGA):

The thermal stability of the prepared catalyst (CS-SO_3H) was evaluated through thermogravimetric analysis (TGA) within the temperature range of 50 – 500 °C. The first weight loss (approximately 5%) at 90 °C is due to the removal of solvent and other small molecules. The degradation of chitosan polysaccharide and SO_3H groups is the second largest loss of approximately 20–75% in the 250–300 °C (Figure S3).

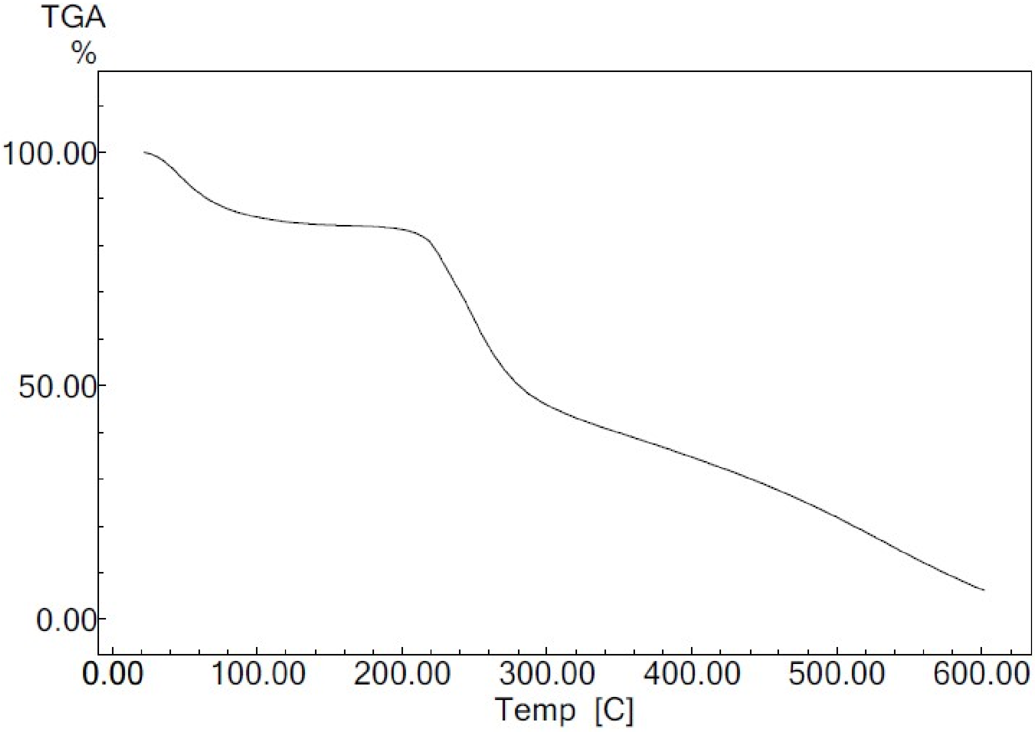

Figure S3: TGA curve of CS- SO_3H.

X-ray Diffraction (XRD) analysis:

The XRD pattern of CS–SO_3H exhibits a broad diffraction peak centered between 15° and 25° (2θ), characteristic of the semicrystalline nature of chitosan. After sulfonation, the peak becomes broader and less intense, indicating increased amorphization due to disruption of intermolecular interactions and hydrogen bonding. This structural change confirms that the introduction of –SO_3H groups reduces crystallinity and enhances the amorphous character of the material (Figure S4).

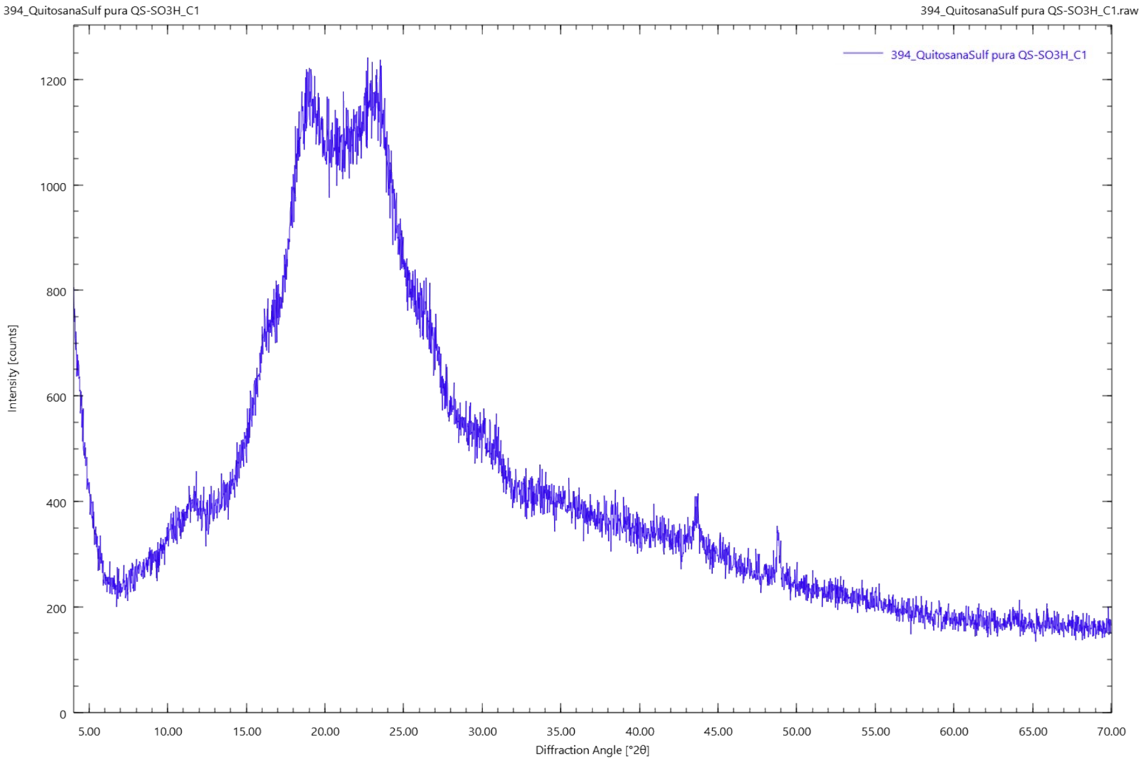

Figure S4: The XRD pattern of CS–SO_3H.

Energy Dispersive X-ray Spectroscopy (EDS) and Field Emission Scanning Electron Microscopy (FE-SEM):

EDS analysis determined the elemental composition of the material, confirming the presence of carbon, oxygen, nitrogen, and sulfur with weight percentages of 61.7%, 19.2%, 13.1%, and 6.0%, respectively. These findings confirmed the successful incorporation of –SO_3H groups on the chitosan backbone (Figure S5). In addition, the surface morphology, particle characteristics, and size distribution of CS-SO_3H were analyzed using FE-SEM (Figure S5). The findings revealed a uniform fibrous surface featuring voids and cracks, which acted as active sites for the specific reaction.

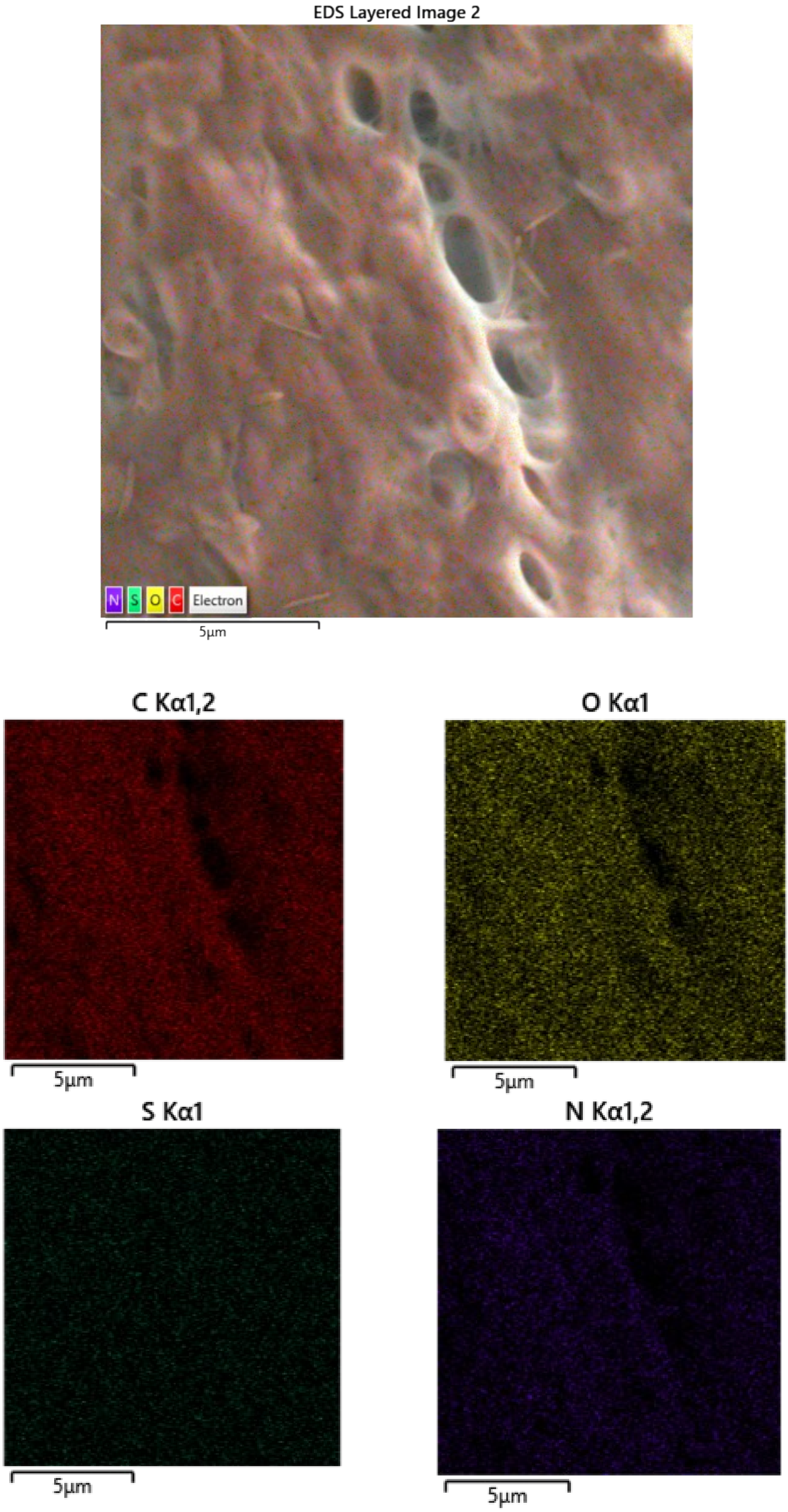
EDS Layered Image 2
N
S
O
C
Electron
5µm
C Kα1,2
5µm
O Kα1
5µm
S Kα1
5µm
N Kα1,2
5µm

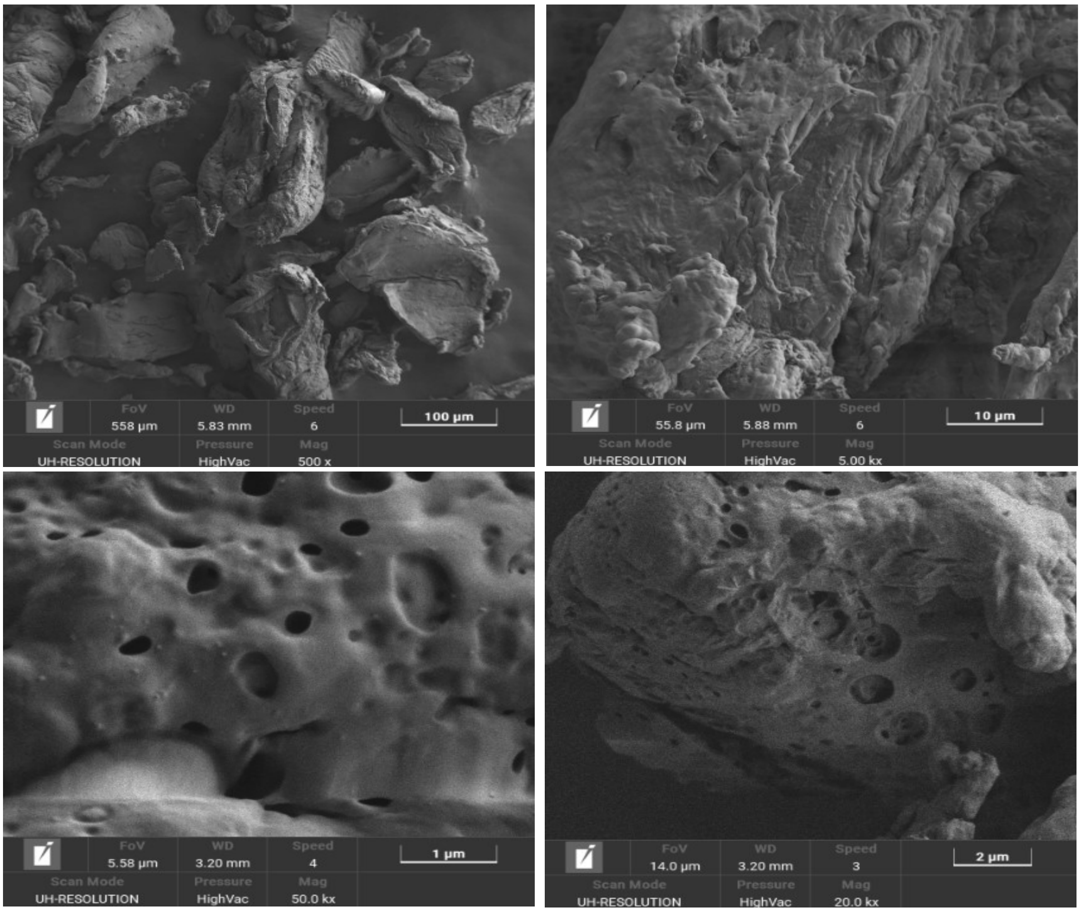

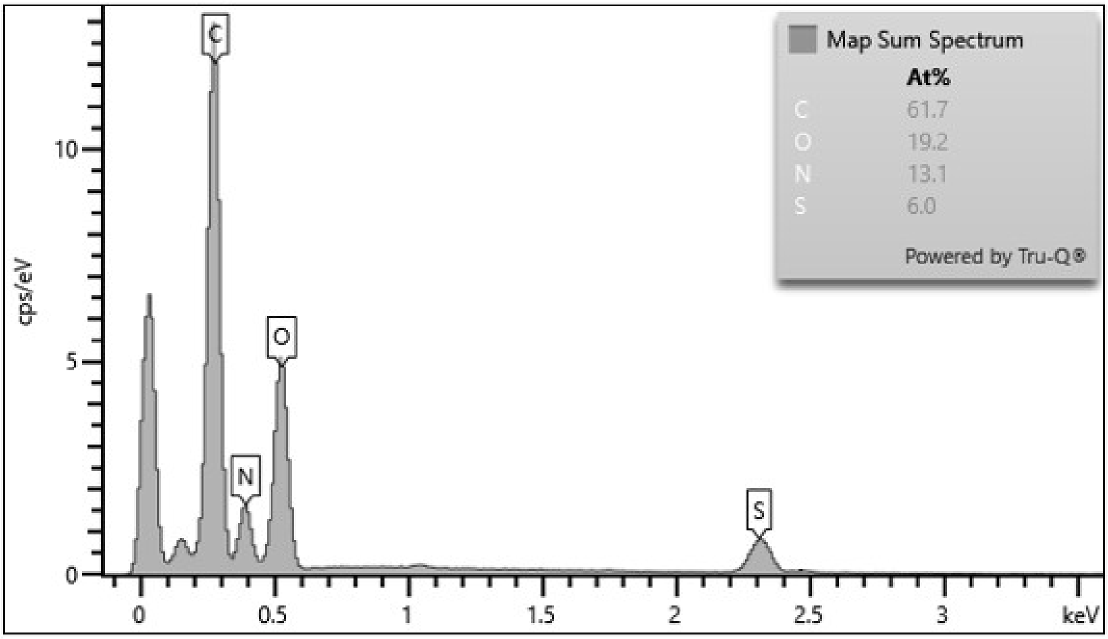

Figure S5: Chitosan-SO_3H EDS spectra.

3. Reused catalyst characterization.

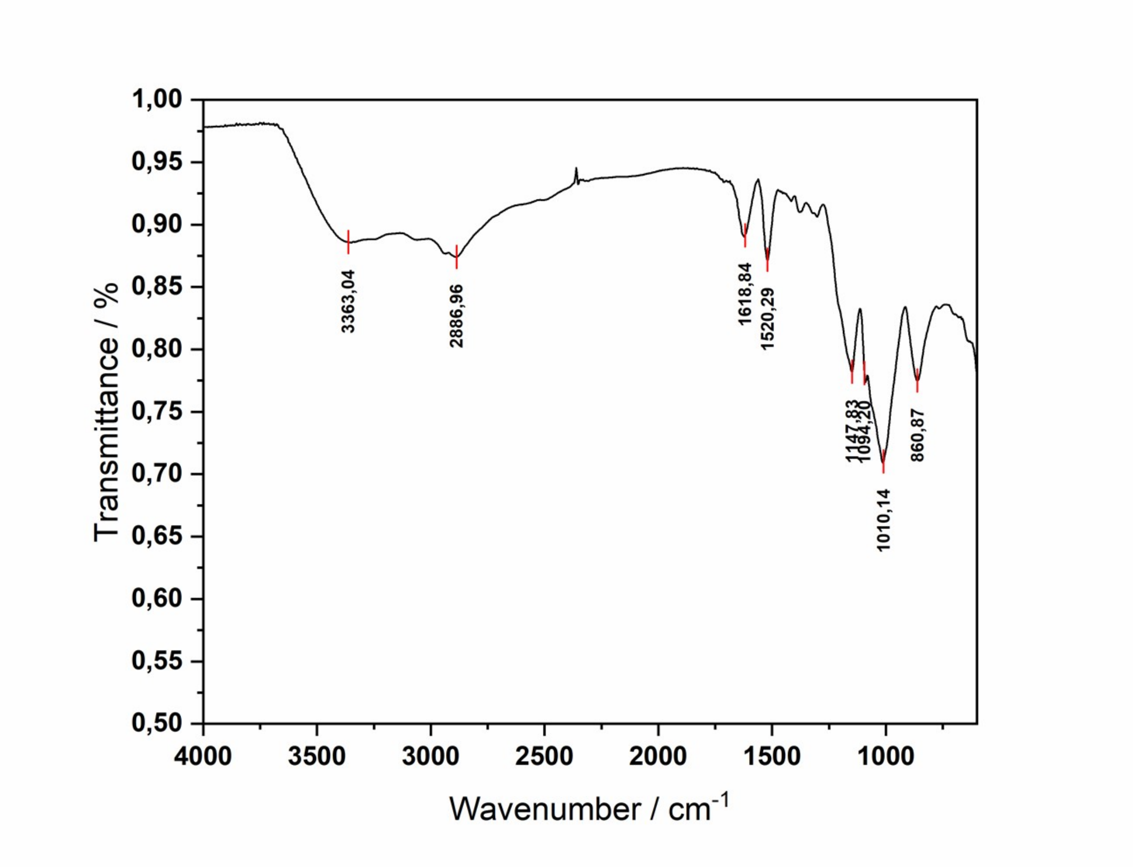

Figure S6: Reused chitosan-SO_3H FT-IR spectra.

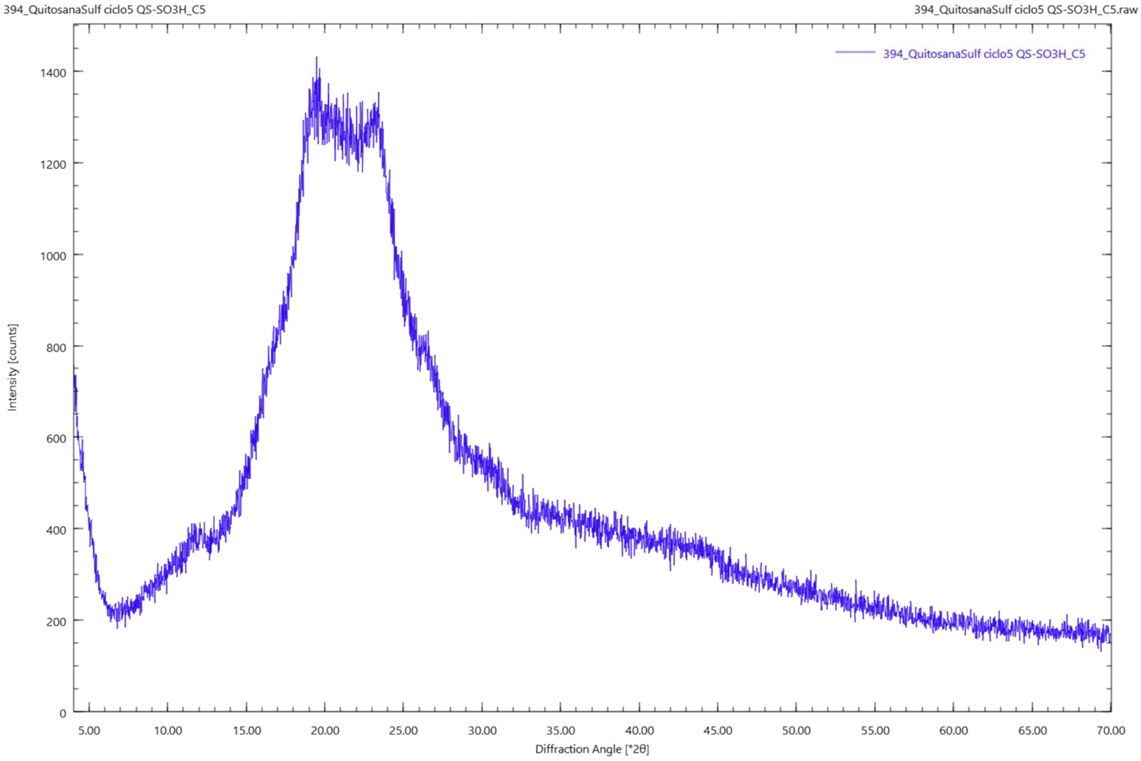

Figure S7: The XRD pattern of Reused CS–SO_3H.

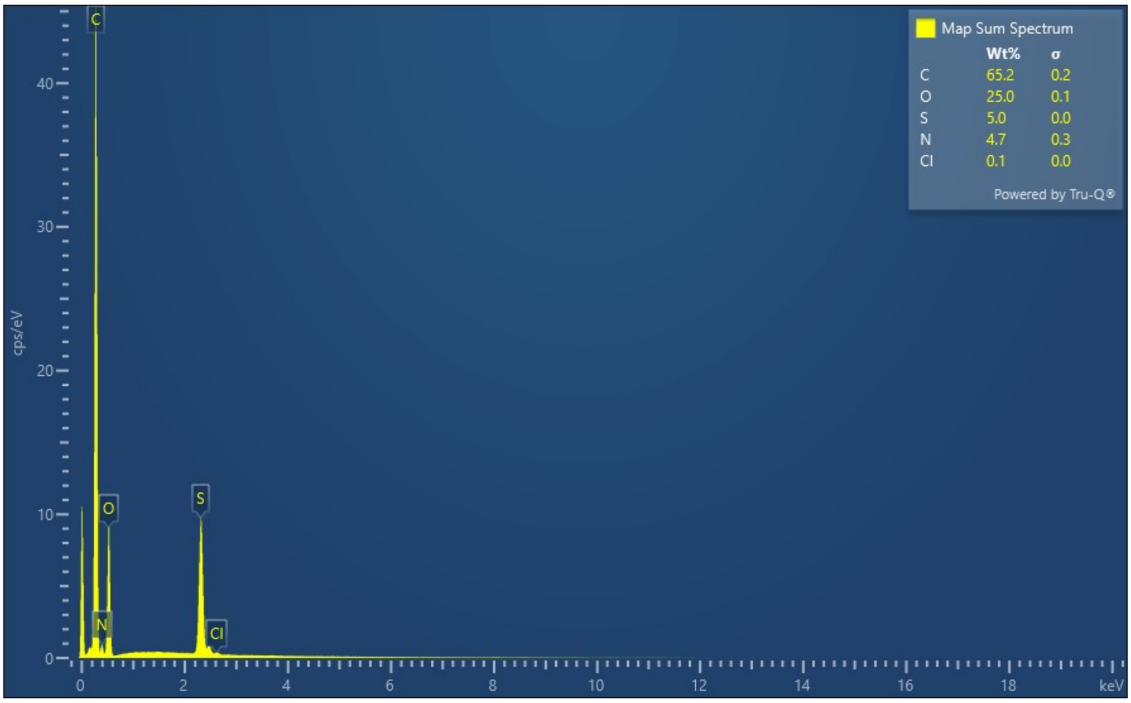

Figure S8: Reused CS–SO₃H EDS spectra.

4. Titration procedure

For each experiment, 300 mg of CS–SO_3H or reused CS–SO_3H was dispersed in 60 mL of 0.1 M KCl solution and stirred for 24 h. The suspension was filtered, and the filtrate was divided into 15.00 mL aliquots. A 0.005 mol·L^{-1} borax solution was prepared by dissolving 0.1938 g of borax in a 100.0 mL volumetric flask. The titration was carried out using methyl red as the indicator to determine the end point. Pure chitosan did not offer any significant acidity and titration was not possible.

	CS-SO_3H	Reused CS-SO_3H
Acidity 1	1,012 mmol/g	0,812 mmol/g
Acidity 2	1,003 mmol/g	0,803 mmol/g
Acidity 3	1,008 mmol/g	0,808 mmol/g
Medium	(1,008 ± 0,005) mmol/g	(0,808 ± 0,005) mmol/g

5. Scale-up Experiment

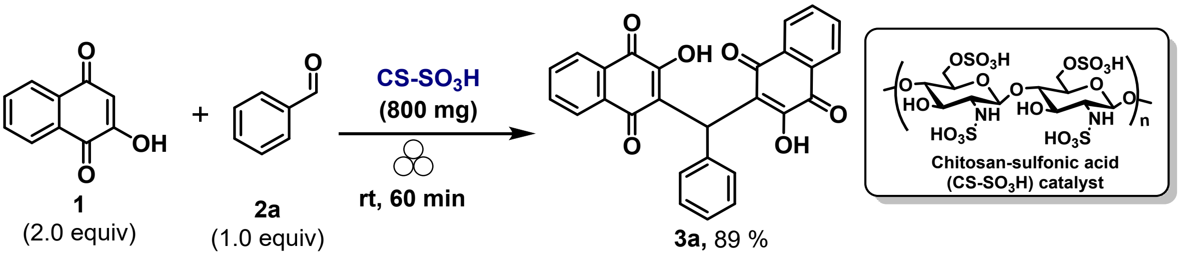

Scheme S2. Scale-up synthesis of **3a**.

In a 15 mL BMT-20-S tube (IKA Ultra-Turrax Tube Drive) containing 10 stainless steel balls (5 mm × 16), 0,5102 mL of benzaldehyde (5.0 mmol, 1.0 equiv.) and 1,74 g of 2-hydroxy-1,4-naphthoquinone (10.0 mmol, 2.0 equiv.) were milled for 10 min at 3000–4000 oscillations *per* minute. Subsequently, chitosan-SO_3H (0.80 g) was added to the mixture and milling was continued for the time indicated in each case under the same oscillation conditions. The reaction progress was monitored by TLC – thus, a small amount amount of the mixture was collected and dissolved in ethanol. After grinding, the crude mixture was transferred to a becker and extracted with 80 mL of ethanol. The solid chitosan-SO_3H was filtered off, washed with hot ethanol, and stored for reuse. The solvent was evaporated under reduced pressure, and the product was recrystallized from ethanol providing compound **3a** as a yellow solid (1,940 mg, 89%).

6. General Procedure for General procedure for mechanosynthesis of 3,3'-(arylmethylene)bis(2- hydroxynaphthalene-1,4-diones)

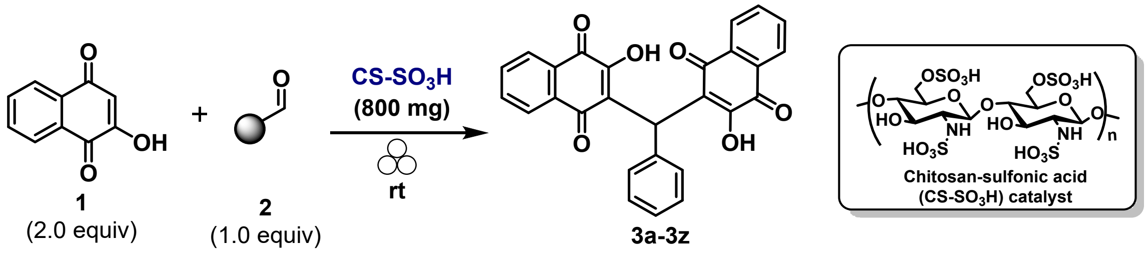

Scheme S3. Synthesis of 3,3'-(arylmethylene)bis(2- hydroxynaphthalene-1,4-diones).

In a 15 mL BMT-20-S tube (IKA Ultra-Turrax Tube Drive) containing 10 stainless steel balls (5 mm × 16), the appropriate aldehyde (1.0 mmol, 1.0 equiv.) and 2-hydroxy-1,4-naphthoquinone (2.0 mmol, 2.0 equiv.) were milled for 5 min at 3000–4000 oscillations *per* minute. In mechanochemistry, pre-milling is important because it reduces particle size, increases surface area, and improves mixing efficiency, which enhances reaction kinetics and product yield. Subsequently, chitosan-SO_3H (0.80 g) was added to the mixture and milling was continued for the time indicated in each case under the same oscillation conditions. The reaction progress was monitored by TLC. After grinding, the crude mixture was transferred to a becker and extracted with 30 mL of ethanol. The solid chitosan-SO_3H was filtered off, washed with hot ethanol, and stored for reuse. The solvent was evaporated under reduced pressure, and the product was recrystallized from ethanol.

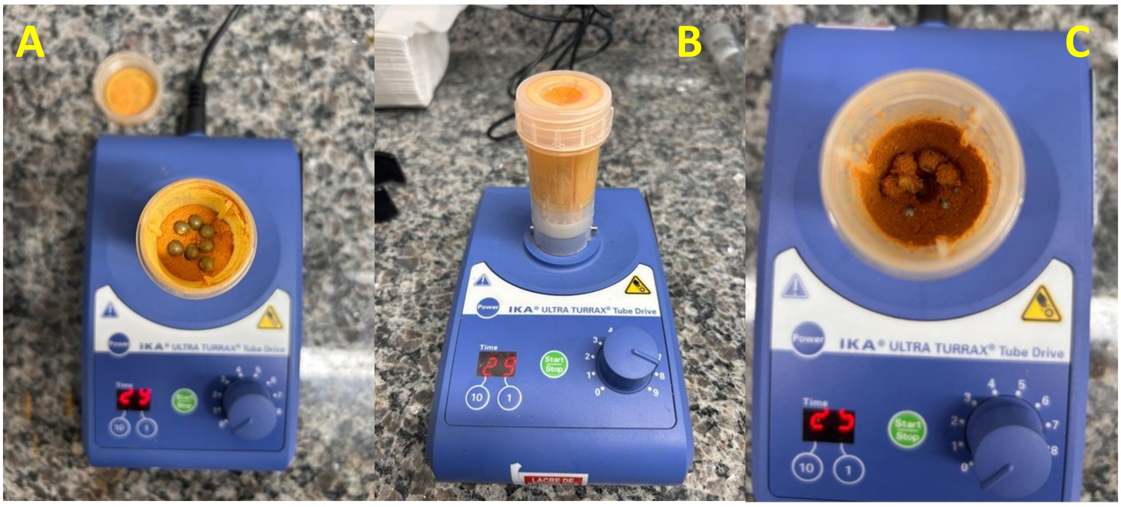

Figure S9: A and B: Set-up of the reaction with solid grinding aid. **C:** Set-up of the reaction without solid grinding aid.

7. Compound Characterization Data

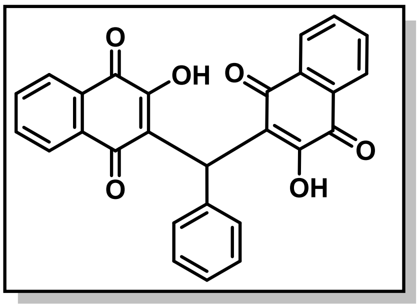

3,3'-(phenylmethylene)bis(2-hydroxynaphthalene-1,4-dione) (3a): *[ref 7]*

01 hour (401 mg, 92% yield), yellow solid, purified by recrystallization in ethanol, mp: 206.2-209.0°C. **^{1}H NMR** (DMSO-d_6, 500 MHz) δ 7.99 (dd, *J* 7.5, 1.5 Hz, 2H), 7.93 (dd, *J* 7.5, 1.5 Hz, 2H), 7.85 – 7.74 (m, 4H), 7.26 – 7.16 (m, 4H), 7.15 – 7.09 (m, 1H), 6.03 (s, 1H). **FT-IR** (ATR, *v*max/cm^{-1}): 3328, 3060, 1641, 1590, 1494, 1299, 1264, 1039, 721.

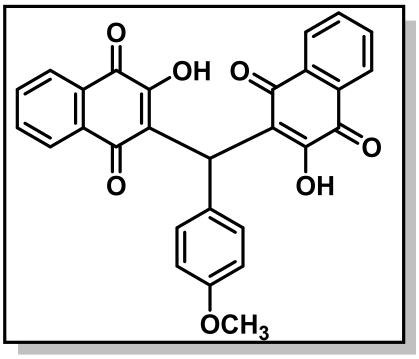

3,3'-((4-methoxyphenyl)methylene)bis(2-hydroxynaphthalene-1,4-dione) (3b): *[ref 7]*

02 hours (433 mg, 93% yield), yellow solid, purified by recrystallization in ethanol, mp: 226.8-230.3°C. **^{1}H NMR** (DMSO-d_6, 500 MHz) δ 7.99 – 7.96 (m, 2H), 7.94 – 7.91 (m, 2H), 7.79 (ddd, *J* 16.1, 7.5, 1.5 Hz, 4H), 7.17 – 7.08 (m, 2H), 6.75 (d, *J* 8.7 Hz, 2H), 5.97 (s, 1H), 3.70 (s, 3H). **FT-IR** (ATR, *v*max/cm^{-1}): 3395, 3239, 1634, 1458, 1333, 1042, 967, 821, 718.

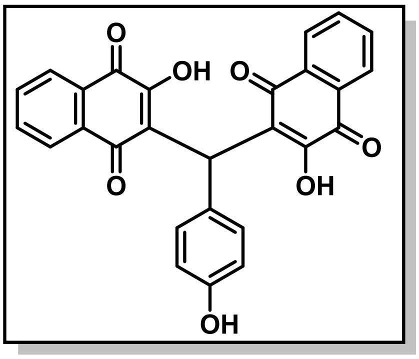

3,3'-((4-hydroxyphenyl)methylene)bis(2-hydroxynaphthalene-1,4-dione) (3c): *[ref 8c]*

02 hours (401 mg, 89% yield), brown solid, purified by recrystallization in ethanol, mp: 207.2-209.0°C. **^{1}H NMR** (DMSO-d_6, 500 MHz) δ 7.99 – 7.95 (m, 2H), 7.93 – 7.90 (m, 2H), 7.85 – 7.72 (m, 5H), 7.05 – 6.96 (m, 2H), 6.62 – 6.54 (m, 2H), 5.92 (s, 1H). **FT-IR** (ATR, *v*max/cm^{-1}): 3388, 3277, 1654, 1450, 1042, 1007, 829, 724, 689.

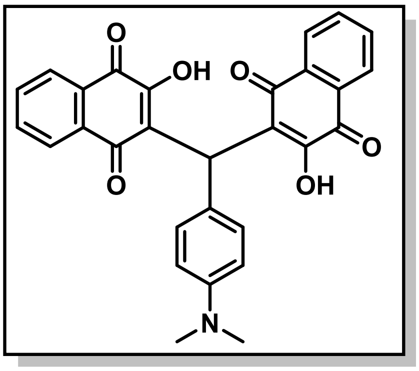

3,3'-((4-(dimethylamino)phenyl)methylene)bis(2-hydroxynaphthalene-1,4-dione) (3d): *[ref 7]*

02 hours (456 mg, 95% yield), yellow solid, purified by recrystallization in ethanol, mp: 154.0-156.8°C. **^{1}H NMR** (DMSO-d_6, 500 MHz) δ 7.94 (ddd, *J* 8.8, 7.6, 1.4 Hz, 4H), 7.83 – 7.70 (m, 4H), 7.12 (d, *J* 8.2 Hz, 2H), 6.15 (s, 1H), 2.93 (s, 6H). **FT-IR** (ATR, *v*max/cm^{-1}): 3537, 2363, 1670, 1460, 1214, 1133, 1050, 912, 838.

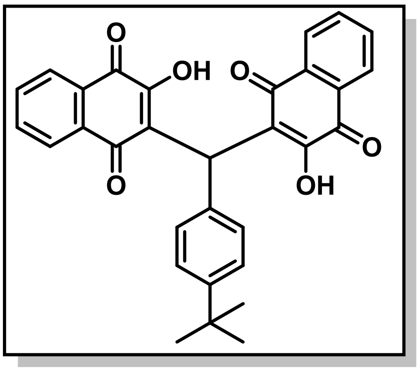

3,3'-((4-(tert-butyl)phenyl)methylene)bis(2-hydroxynaphthalene-1,4-dione) (3e): previously unreported compound

03 hours (449 mg, 91% yield), yellow solid, purified by recrystallization in ethanol, mp: 226.7-227.7°C. **^{1}H NMR** (DMSO-d_6, 500 MHz) δ 8.02 – 7.70 (m, 8H), 7.33 – 7.02 (m, 4H), 6.00 (s, 1H), 1.25 (s, 9H). **^{13}C{^{1}H} NMR** (DMSO-d_6, 125 MHz) δ 31.7, 34.4, 37.6, 123.7, 124.8, 126.0, 126.5, 128.3, 130.3, 132.7, 133.5, 135.1, 138.1, 147.9, 156.6, 181.6, 184.0. **FT-IR** (ATR, *v*max/cm^{-1}): 3239, 2957, 1639, 1345, 1277, 1050, 904, 830, 791. **HRMS (ESI):** m/z calc. for $C_{31}H_{24}NaO_6$ $[M+Na]^+$ 515.1465, found 515.1458.

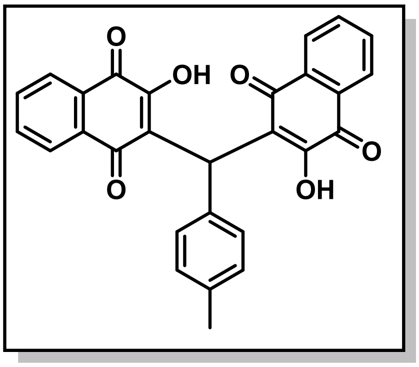

3,3'-(p-tolylmethylene)bis(2-hydroxynaphthalene-1,4-dione) (3f): *[ref 8b]*

03 hours (421 mg, 93% yield), yellow solid, purified by recrystallization in ethanol, mp: 179.1-182.0°C. **^{1}H NMR** (DMSO-d$_6$, 500 MHz) δ 7.98 (dd, *J* 7.4, 1.5 Hz, 2H), 7.92 (dd, *J* 7.6, 1.4 Hz, 2H), 7.85 – 7.74 (m, 4H), 7.11 (d, *J* 7.9 Hz, 2H), 6.99 (d, *J* 7.9 Hz, 2H), 5.99 (s, 1H), 2.24 (s, 3H). **FT-IR** (ATR, *v*max/cm^{-1}): 3335, 3027, 1653, 1626, 1459, 1301, 897, 866, 722.

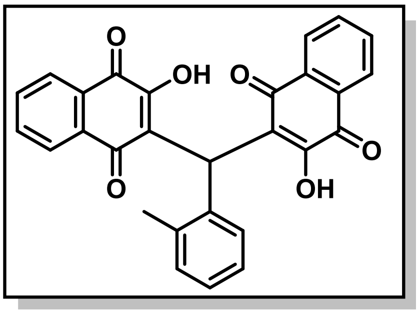

3,3'-(o-tolylmethylene)bis(2-hydroxynaphthalene-1,4-dione)(3g): ***[ref 3c]***

01 hour (431 mg, 96% yield), yellow solid, purified by recrystallization in ethanol, mp: 220.6-223.7°C. **^{1}H NMR** (DMSO-d$_6$, 500 MHz) δ 8.02 – 7.96 (m, 2H), 7.95 – 7.90 (m, 2H), 7.85 – 7.74 (m, 4H), 7.17 (dd, *J* 7.4, 1.6 Hz, 1H), 7.11 – 6.98 (m, 3H), 6.01 (s, 1H), 2.21 (s, 3H). **FT-IR** (ATR, *v*max/cm^{-1}): 3290, 3247, 1671, 1630, 1459, 1336, 1270, 779, 724.

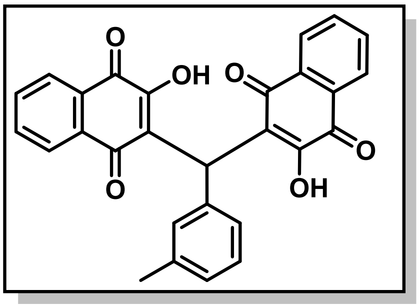

3,3'-(m-tolylmethylene)bis(2-hydroxynaphthalene-1,4-dione)(3h): ***[ref 8c]***

03 hours (382 mg, 85% yield), yellow solid, purified by recrystallization in ethanol, mp: 221.2-223.7°C. **^{1}H NMR** (DMSO-d$_6$, 500 MHz) δ 8.01 – 7.96 (m, 2H), 7.95 – 7.90 (m, 2H), 7.80 (ddd, *J* 15.8, 7.5, 1.5 Hz, 4H), 7.11 – 6.98 (m, 3H), 6.95 – 6.89 (m, 1H), 6.01 (s, 1H), 2.22 (s, 3H). **FT-IR** (ATR, *v*max/cm^{-1}): 3342, 2901, 1654, 1628, 1459, 901, 860, 755, 698.

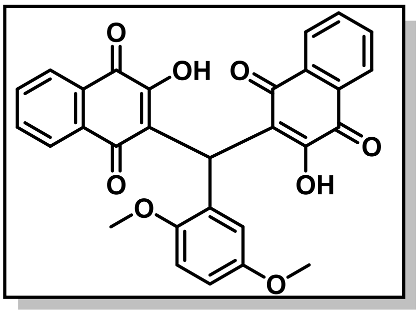

3,3'-((2,5-dimethoxyphenyl)methylene)bis(2-hydroxynaphthalene-1,4-dione) (3i): previously unreported compound

01 hours (481 mg, 97% yield), orange solid, purified by recrystallization in ethanol, mp: 217.8-218.7°C. **^{1}H NMR** (DMSO-d$_6$, 500 MHz) δ 8.09 (ddd, *J* 11.8, 7.7, 1.4 Hz, 5H), 7.80 – 7.58 (m, 7H), 6.74 (s, 3H), 6.29 (s, 1H), 3.71 (d, *J* 9.6 Hz, 6H). **^{13}C{^{1}H} NMR** (DMSO-d$_6$, 125 MHz) δ 33.6, 55.5, 56.5, 110.3, 111.2, 116.8, 123.8, 126.0, 126.5, 130.2, 130.6, 132.6, 133.5, 135.1,

152.0, 153.3, 156.2, 181.5, 183.7; **FT-IR** (ATR, *v*max/cm^{-1}): 3206, 3129, 3038, 2835, 1652, 1444, 1303, 920, 861. **HRMS (ESI):** m/z calc. for $C_{29}H_{20}NaO_8$ $[M+Na]^+$ 519,4608, found 519.1046.

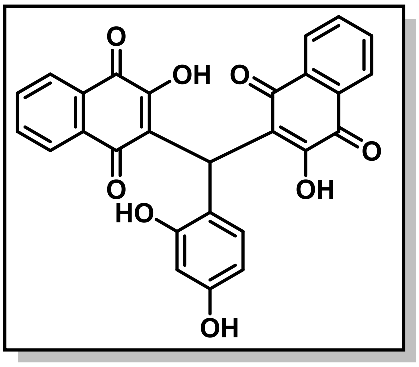

3,3'-((2,4-dihydroxyphenyl)methylene)bis(2-hydroxynaphthalene-1,4-dione)(3j): previously unreported compound

02 hours (159 mg, 92% yield), orange solid, purified by recrystallization in ethanol, mp: 251.9-255.7°C. **^{1}H NMR** (DMSO-d_6, 500 MHz) δ 8.04 – 7.92 (m, 4H), 7.87 – 7.75 (m, 5H), 6.93 (d, *J* 8.4 Hz, 1H), 6.65 – 6.49 (m, 2H), 6.18 (s, 1H), 5.65 (s, 1H); **^{13}C{^{1}H} NMR** (DMSO-d_6, 125 MHz) δ 103.3, 111.5, 113.6, 125.8, 126.1, 126.2, 126.4, 129.7, 130.3, 130.7, 131.0, 131.6, 132.0, 132.4,133.6,134.3,134.9, 135.0, 135.2, 149.8, 157.9, 178.3, 181.7, 183.4; **FT-IR** (ATR, *v*max/cm^{-1}): 3374, 1672, 1642, 1457, 1308, 1210, 904, 867, 797.

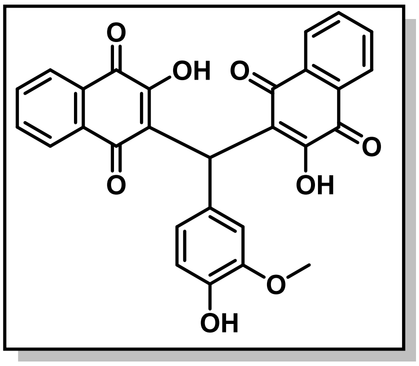

3,3'-((4-hydroxy-3-methoxyphenyl)methylene)bis(2-hydroxynaphthalene-1,4-dione) (3k): *[ref 7]*

02 hours (433 mg, 94% yield), yellow solid, purified by recrystallization in ethanol, mp: 224.3-228.6°C. **^{1}H NMR** (DMSO-d_6, 500 MHz) δ 8.03 – 7.90 (m, 5H), 7.85 – 7.74 (m, 5H), 6.80 (s, 1H), 6.59 (d, *J* = 2.1 Hz, 2H), 5.94 (s, 1H), 3.64 (s, 3H); **FT-IR** (ATR, *v*max/cm^{-1}):): 3326, 1637, 1460, 1245, 1202, 1042, 903, 861, 800.

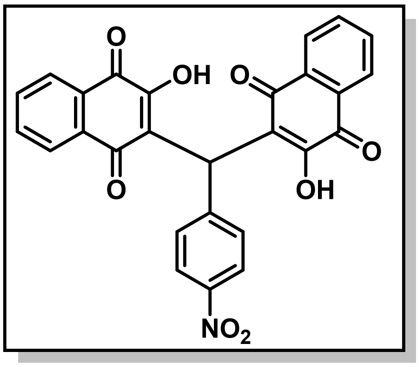

3,3'-((4-nitrophenyl)methylene)bis(2-hydroxynaphthalene-1,4-dione) (3l): *[ref 8b]*

03 hours (412 mg, 85% yield), orange solid, purified by recrystallization in ethanol, mp: 182.4-185.6°C. **^{1}H NMR** (DMSO-d_6, 500 MHz) δ 8.12 – 7.88 (m, 6H), 7.87 – 7.75 (m, 4H), 7.58 – 7.51 (m,

2H), 6.09 (s, 1H). **FT-IR** (ATR, *v*max/cm^{-1}): 3324, 1644, 1459, 1348, 1294, 1052, 1017, 858, 820.

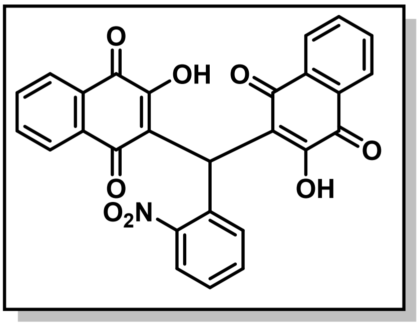

3,3'-((2-nitrophenyl)methylene)bis(2-hydroxynaphthalene-1,4-dione)(3m): *[ref 8c]*

03 hours (378 mg, 78% yield), yellow solid, purified by recrystallization in ethanol, mp: 212.9-214.0°C. **^{1}H NMR** (DMSO-d$_6$, 500 MHz) δ 8.01 – 7.90 (m, 6H), 7.88 – 7.74 (m, 8H), 7.57 – 7.50 (m, 3H), 7.47 – 7.40 (m, 2H), 6.42 (s, 1H). **FT-IR** (ATR, *v*max/cm^{-1}): 3353, 3083, 1643, 1607, 1578, 1460, 1297, 1249, 902.

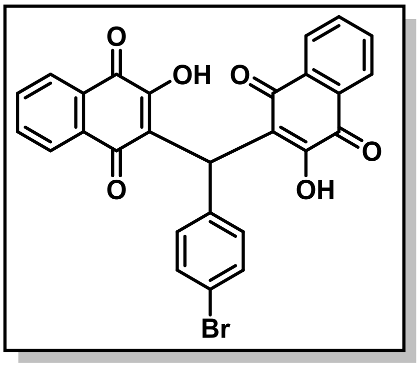

3,3'-((4-bromophenyl)methylene)bis(2-hydroxynaphthalene-1,4-dione) (3n): *[ref 7]*

02 hours (458 mg, 89% yield), yellow solid, purified by recrystallization in ethanol, mp: 200.6-202.9°C. **^{1}H NMR** (DMSO-d$_6$, 500 MHz) δ 8.03 – 7.88 (m, 4H), 7.86 – 7.73 (m, 4H), 7.36 (d, *J* 8.5 Hz, 2H), 7.20 (d, *J* 8.5 Hz, 2H), 5.97 (s, 1H); **FT-IR** (ATR, *v*max/cm^{-1}): 3335, 1653, 1641, 1459, 1277, 1209, 1300, 897, 826.

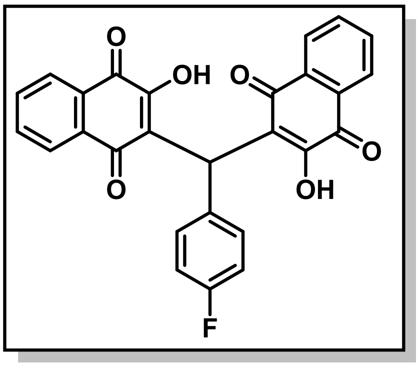

3,3'-((4-fluorophenyl)methylene)bis(2-hydroxynaphthalene-1,4-dione) (3o): *[ref 8c]*

03 hours (396 mg, 87% yield), yellow solid, purified by recrystallization in ethanol, mp: 165.0-168.3°C. **^{1}H NMR** (DMSO-d$_6$, 500 MHz) δ 8.03 – 7.88 (m, 4H), 7.86 – 7.73 (m, 4H), 7.27 (d, *J* 3.6 Hz, 2H), 7.00 (t, *J* 8.9 Hz, 2H), 5.98 (s, 1H). **FT-IR** (ATR, *v*max/cm^{-1}): 3294, 1638, 1459, 1274, 1213, 1007, 905, 797, 721.

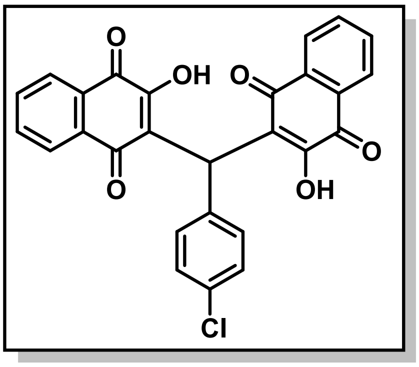

3,3'-((4-chlorophenyl)methylene)bis(2-hydroxynaphthalene-1,4-dione)(3p): *[ref 7]*

02 hours (461 mg, 98% yield), yellow solid, purified by recrystallization in ethanol, mp:

171.1-175.5°C. **^{1}H NMR** (DMSO-d_6, 500 MHz) δ 8.07 – 7.89 (m, 4H), 7.88 – 7.71 (m, 4H), 7.33 – 7.16 (m, 4H), 5.99 (s, 1H); **FT-IR** (ATR, νmax/cm^{-1}): 3339, 1641, 1590, 1459, 1300, 1277, 898, 808, 722.

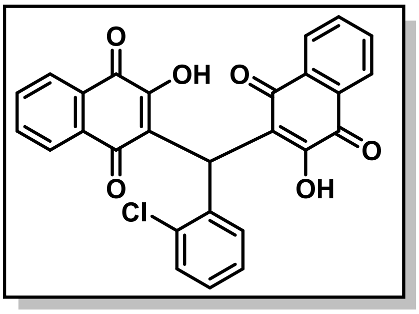

3,3'-((2-chlorophenyl)methylene)bis(2-hydroxynaphthalene-1,4-dione)(3q): *[ref 8c]*

02 hours (320 mg, 68% yield), yellow solid, purified by recrystallization in ethanol, mp: 226.6-230.6°C. **^{1}H NMR** (DMSO-d_6, 500 MHz) δ 8.00 (dd, J 7.4, 1.5 Hz, 2H), 7.93 (dd, J 7.7, 1.5 Hz, 2H), 7.87 – 7.75 (m, 4H), 7.33 (td, J 7.7, 2.1 Hz, 2H), 7.25 – 7.14 (m, 2H), 6.10 (s, 1H); **FT-IR** (ATR, νmax/cm^{-1}): 3410, 3224, 3071, 1663, 1635, 1470, 1299, 873, 820.

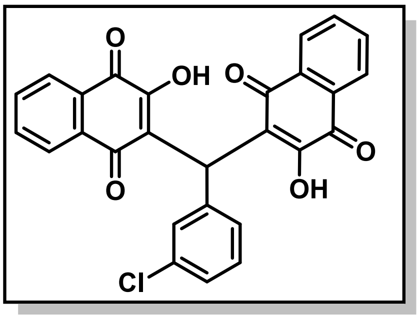

3,3'-((3-chlorophenyl)methylene)bis(2-hydroxynaphthalene-1,4-dione) (3r): *[ref 8c]*

03 hours (412 mg, 87% yield), yellow solid, purified by recrystallization in ethanol, mp: 225.2-228.8°C. **^{1}H NMR** (DMSO-d_6, 500 MHz) δ 8.02 – 7.90 (m, 4H), 7.86 – 7.73 (m, 4H), 7.32 – 7.12 (m, 4H), 6.00 (s, 1H); **FT-IR** (ATR, νmax/cm^{-1}): 3345, 1643, 1627, 1297, 1265, 907, 816, 790, 691.

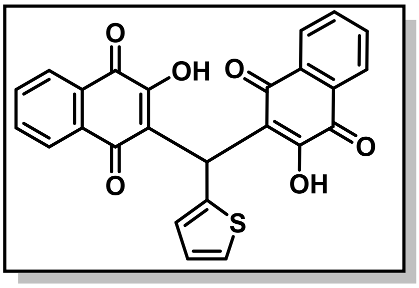

3,3'-(thiophen-2-ylmethylene)bis(2-hydroxynaphthalene-1,4-dione) (3s): *[ref 33]*

03 hours (405 mg, 91% yield), yellow solid, purified by recrystallization in ethanol, mp: 201.2-204.6°C. **^{1}H NMR** (DMSO-d_6, 500 MHz) δ 7.96 (ddd, J 12.0, 7.6, 1.4 Hz, 4H), 7.86 – 7.74 (m, 4H), 7.25 (dd, J 4.9, 1.5 Hz, 1H), 6.89 – 6.79 (m, 2H), 6.30 (d, J 1.1 Hz, 1H); **FT-IR** (ATR, νmax/cm^{-1}): 3254, 1672, 1638, 1459, 1343, 1267, 972, 726, 690.

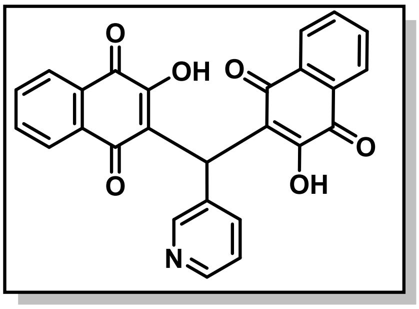

3,3'-(pyridin-3-ylmethylene)bis(2-hydroxynaphthalene-1,4-dione) (3t): *[ref 4]*

03 hours (400 mg, 91% yield), orange solid, purified by recrystallization in ethanol, mp: 230.0-233.2°C. **^{1}H NMR** (DMSO-d_6, 500 MHz) δ 8.72 – 8.52 (m, 2H), 8.23 (d, J 8.3 Hz, 1H), 8.01 – 7.89 (m, 4H), 7.84 – 7.68 (m, 6H), 6.62 (s, 1H); **FT-IR** (ATR, νmax/cm^{-1}): 3440, 3056, 2887, 2455, 1640, 1251, 1080, 998, 917.

3,3'-(pyridin-4-ylmethylene)bis(2-hydroxynaphthalene-1,4-dione) (3u): previously unreported compound

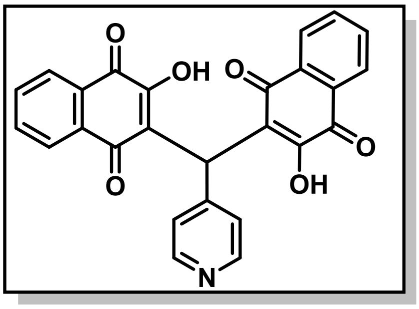

03 hours (411 mg, 94% yield), orange solid, purified by recrystallization in ethanol, mp: 247.0-251.6°C. **^{1}H NMR** (DMSO-d_6, 500 MHz) δ 8.61 (d, J 6.9 Hz, 2H), 8.01 – 7.89 (m, 4H), 7.84 – 7.76 (m, 4H), 7.71 (td, J 7.5, 1.4 Hz, 2H), 6.78 (s, 1H). **^{13}C{^{1}H} NMR** (DMSO-d_6, 125 MHz) δ 35.4,120.5, 125.6, 125.8, 126.3, 131.4, 132.7, 133.4, 134.4, 142.2, 162.9, 164.1, 182.6, 183.2; **FT-IR** (ATR, νmax/cm^{-1}): 3059, 2856, 1671, 1637, 1278, 1059, 912, 874, 801. **HRMS (ESI):** m/z calc. for $C_{26}H_{16}NO_6$ $[M + H]^+$ 438.0978 found 438.0966.

3,3'-((1-phenyl-1H-1,2,3-triazol-4-yl)methylene)bis(2-hydroxynaphthalene-1,4-dione) (3v): *[ref 34]*

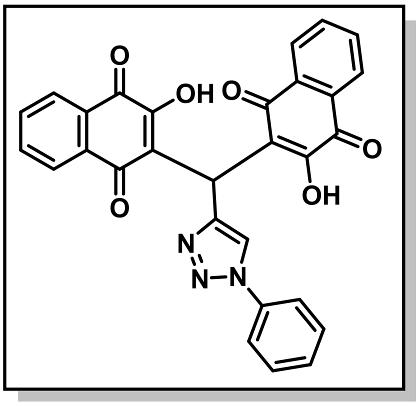

02 hours (251 mg, 85% yield), yellow solid, purified by recrystallization in ethanol, mp: 206.8-210.4°C. **^{1}H NMR** (DMSO-d_6, 500 MHz) δ 8.56 (s, 1H), 8.04 – 7.93 (m, 4H), 7.89 – 7.76 (m, 6H), 7.54 (t, J 8.0 Hz, 2H), 7.47 – 7.39 (m, 1H), 6.13 (s, 1H); **FT-IR** (ATR, νmax/cm^{-1}): 3290, 3145, 1649, 1460, 1252, 1047, 1028, 901, 726.

3,3'-((1-(4-nitrophenyl)-1H-1,2,3-triazol-4-yl)methylene)bis(2-hydroxynaphthalene-1,4-dione) (3w): *[ref 34]*

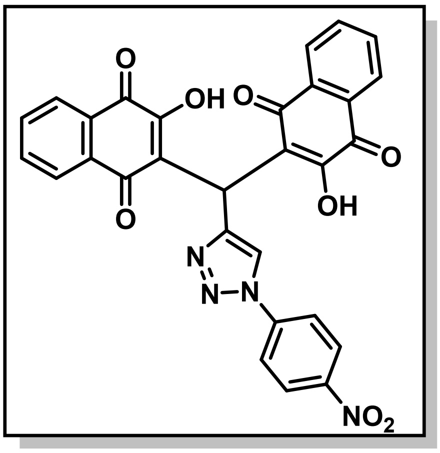

03 hours (320 mg, 84% yield), yellow solid, purified by recrystallization in ethanol, mp:

208.9-210.7°C. ^{1}**H NMR** (DMSO-d_6, 500 MHz) δ 8.77 (s, 1H), 8.41 (d, J 9.3 Hz, 2H), 8.18 (d, J 9.3 Hz, 2H), 8.06 – 7.93 (m, 4H), 7.90 – 7.75 (m, 4H), 6.14 (s, 1H); **FT-IR** (ATR, νmax/cm^{-1}): 3348, 3132, 3077, 1680, 1643, 1458, 1306, 1009, 903.

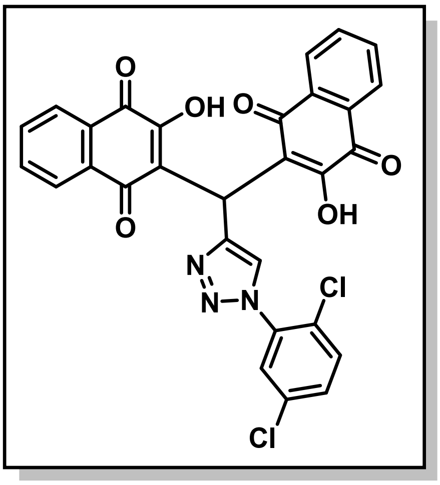

3,3'-((1-(2,5-dichlorophenyl)-1H-1,2,3-triazol-4-yl)methylene)bis(2-hydroxynaphthalene-1,4-dione) (3x): *[ref 34]*

02 hours (472 mg, 94% yield), yellow solid, purified by recrystallization in ethanol, mp: 206.6-208.6°C. ^{1}**H NMR** (DMSO-d_6, 500 MHz) δ 10.14 (s, 1H), 9.36 (s, 1H), 8.03 – 7.99 (m, 3H), 7.94 (dd, J 7.3, 1.4 Hz, 2H), 7.86 – 7.77 (m, 7H), 6.17 (s, 1H); **FT-IR** (ATR, νmax/cm^{-1}): 3139, 1699, 1678, 1633, 1342, 1280, 1043, 808, 725.

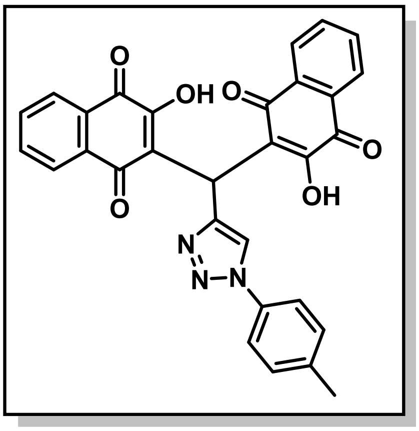

3,3'-((1-(p-tolyl)-1H-1,2,3-triazol-4-yl)methylene)bis(2-hydroxynaphthalene-1,4-dione) (3y): previously unreported compound

02 hours (490 mg, 94% yield), yellow solid, purified by recrystallization in ethanol, mp: 217.9-220.0°C. ^{1}**H NMR** (DMSO-d_6, 500 MHz) δ 8.59 (d, J 0.9 Hz, 1H), 8.03 – 7.87 (m, 6H), 7.86 – 7.74 (m, 4H), 7.64 – 7.58 (m, 2H), 6.11 (d, J 0.9 Hz, 1H), 2.07 (s, 3H). ^{13}**C{^{1}H} NMR** (DMSO-d_6, 125 MHz) δ 20.9, 30.2, 119.6, 121.2, 122.1, 126.0, 126.5, 130.4, 130.5, 132.7, 133.5, 135.1, 138.0, 148.0, 157.1, 181.7, 183.6; **FT-IR** (ATR, νmax/cm^{-1}): 3329, 3146, 1649, 1588, 1459, 1339, 1046, 899, 817. **HRMS (ESI):** m/z calc. for $C_{30}H_{19}N_3NaO_6$ $[M+Na]^+$ 540.1172, found 540.1170.

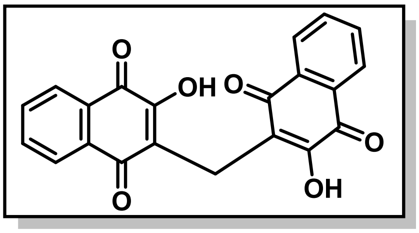

3,3'-methylenebis(2-hydroxynaphthalene-1,4-dione) (3z): *[ref 6]*

01 hour (350 mg, 97% yield), yellow solid, purified by recrystallization in ethanol, mp: 251.5-254.0°C. ^{1}**H NMR** (DMSO-d_6, 500 MHz)

δ 7.97 (ddd, *J* 7.6, 6.2, 1.4 Hz, 4H), 7.88 – 7.71 (m, 4H), 3.76 (s, 2H); **FT-IR** (ATR, *v*max/cm^{-1}): 3071, 1678, 1457, 1307, 1262, 1210, 1069, 974, 732.

8. ^{1}H, ^{13}C NMR and FT-IR spectra

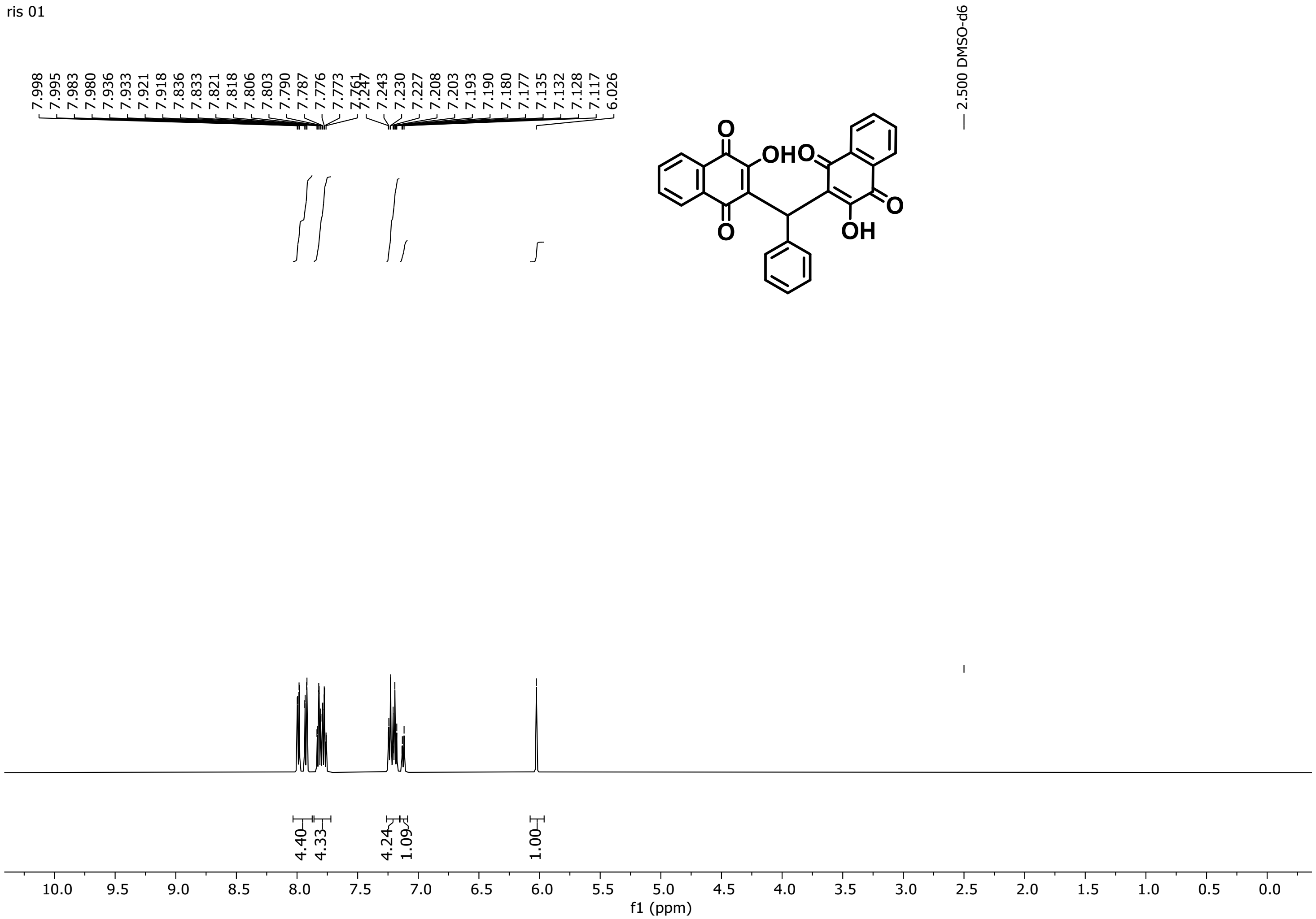

Figure S10. ^{1}H NMR spectrum of **3a** (500 MHz, DMSO-d_6).

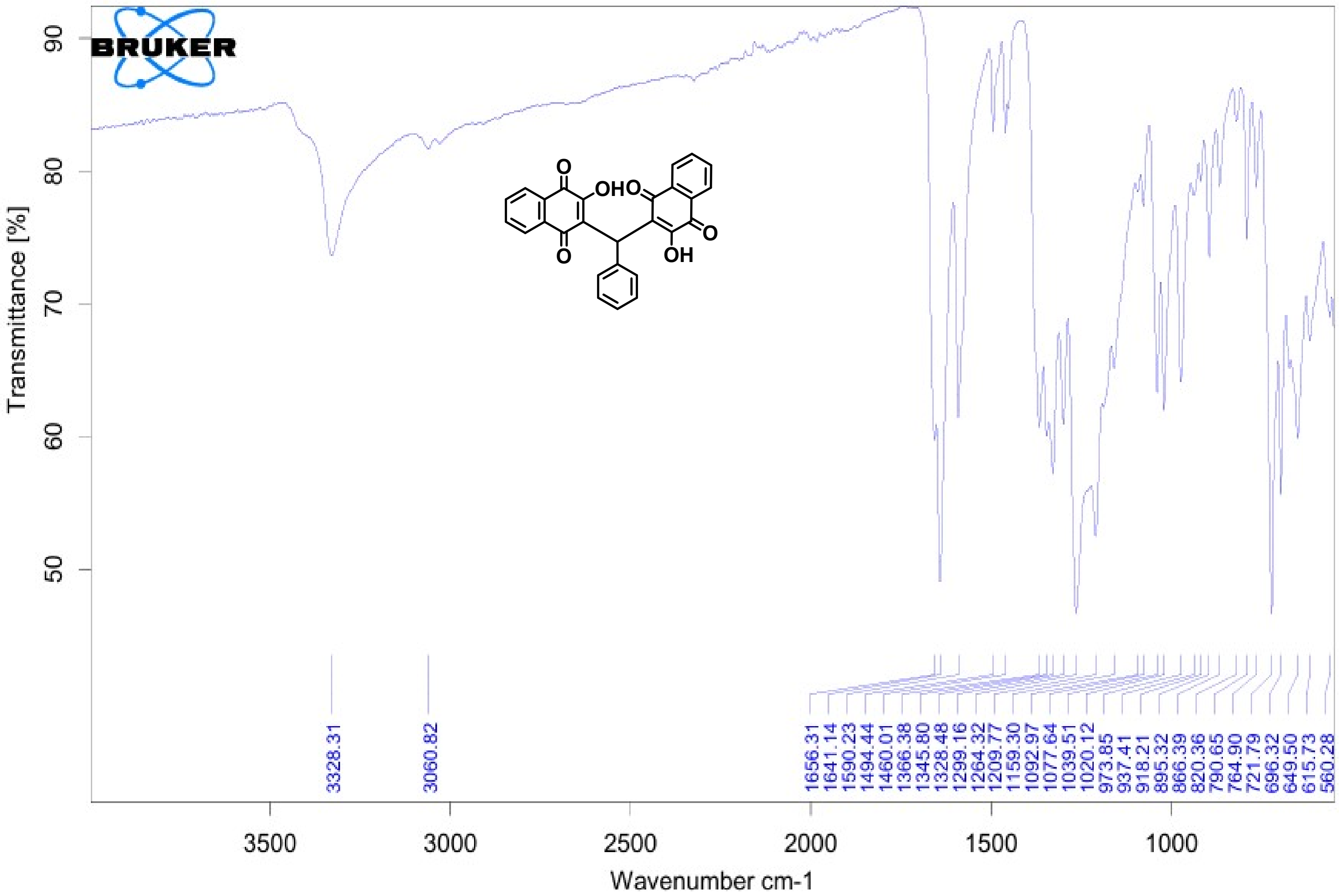

Figure S11. FT-IR spectrum of **3a**.

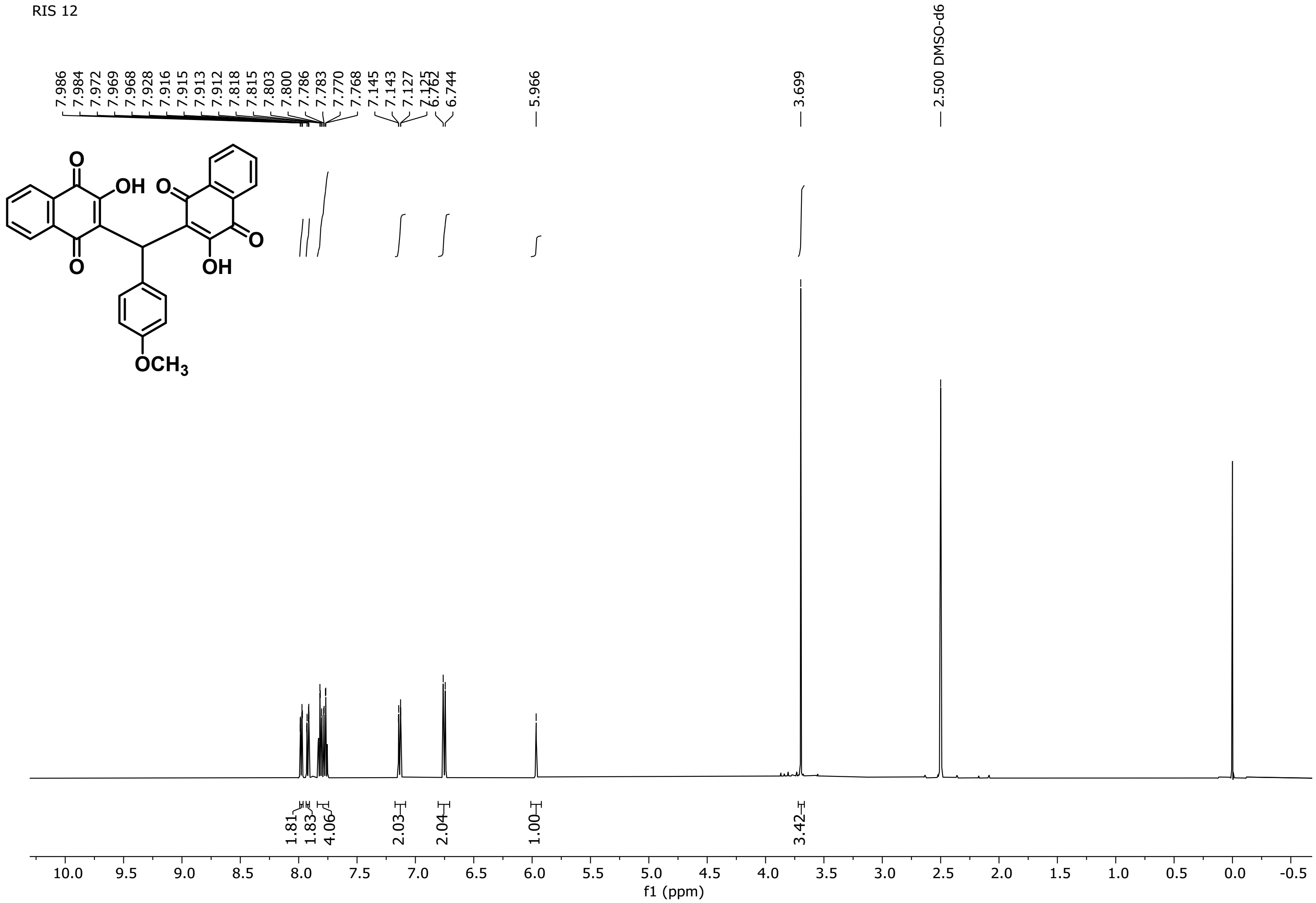

Figure S12. ^{1}H NMR spectrum of **3b** (500 MHz, DMSO-d$_6$).

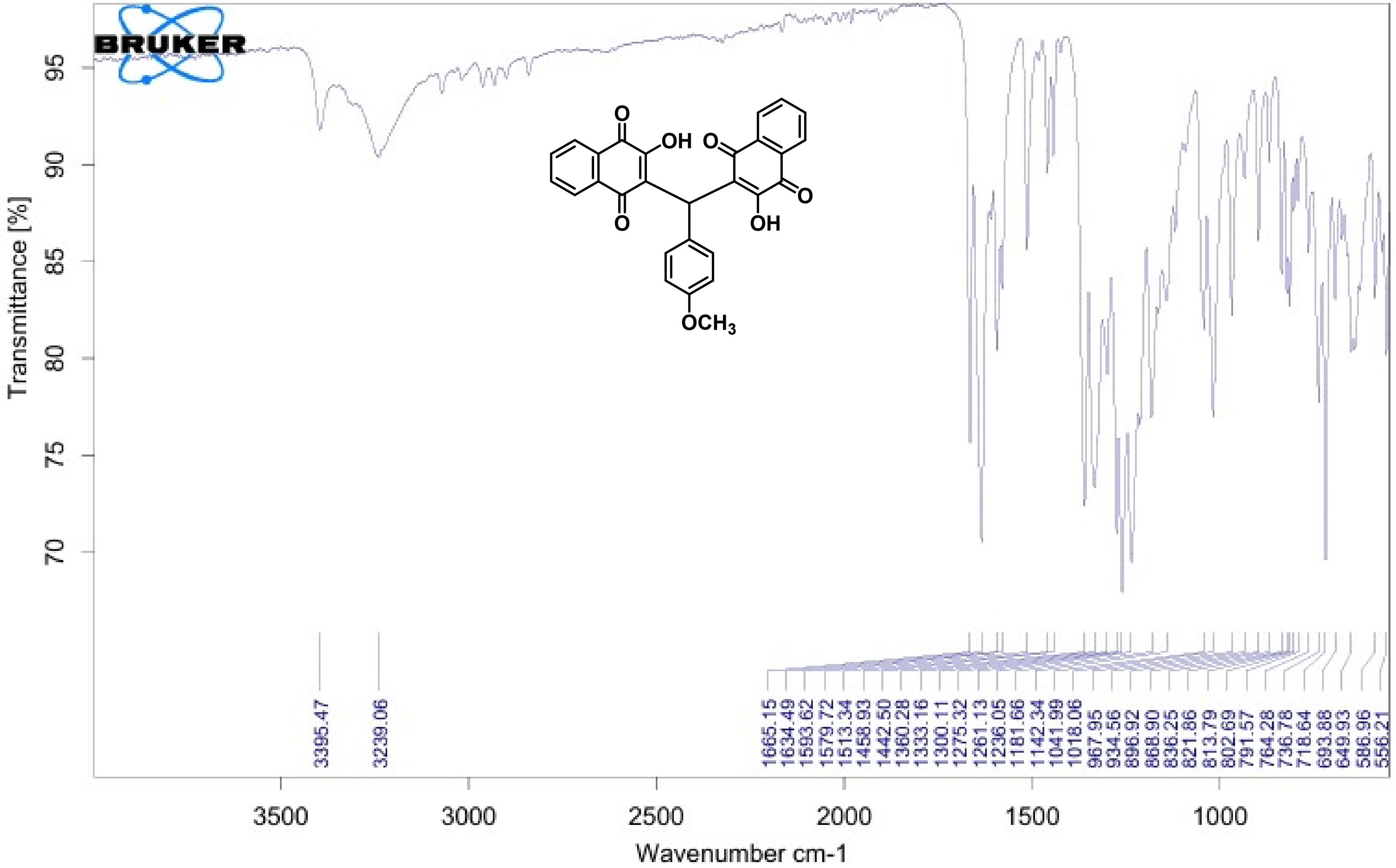

Figure S13. FT-IR spectrum of **3b**.

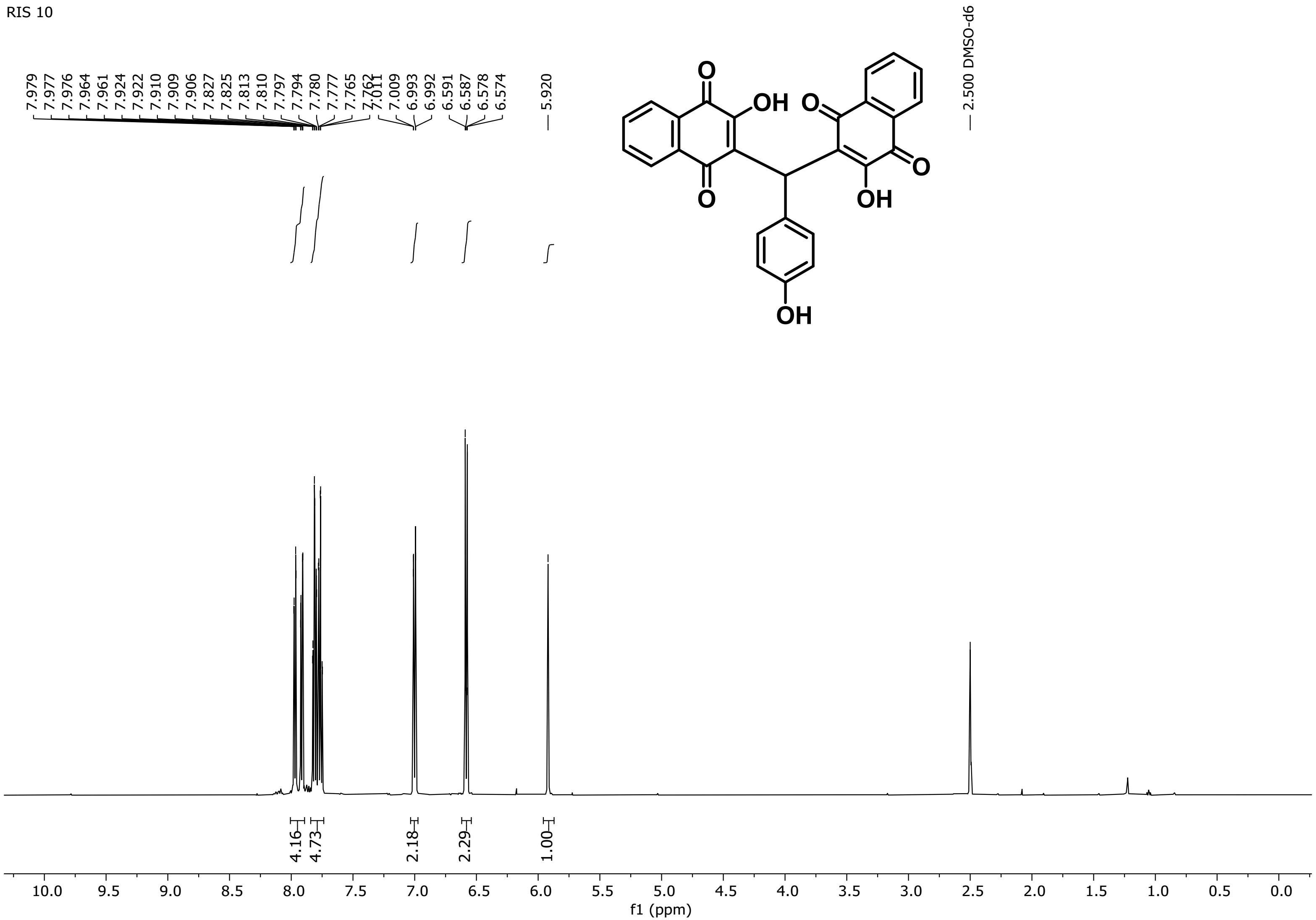

Figure S14. ^{1}H NMR spectrum of **3c** (500 MHz, DMSO-d$_6$).

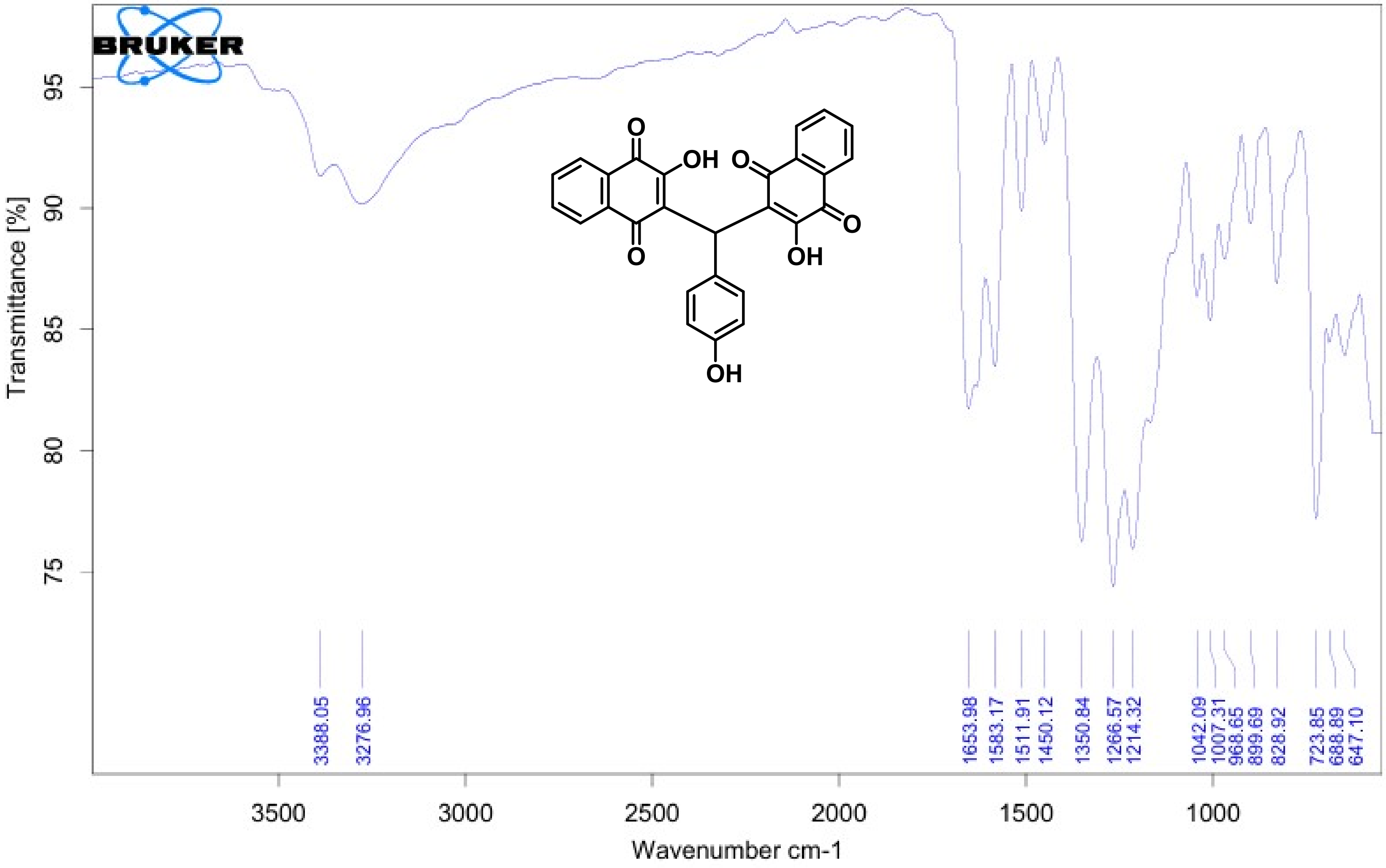

Figure S15. FT-IR spectrum of **3c**.

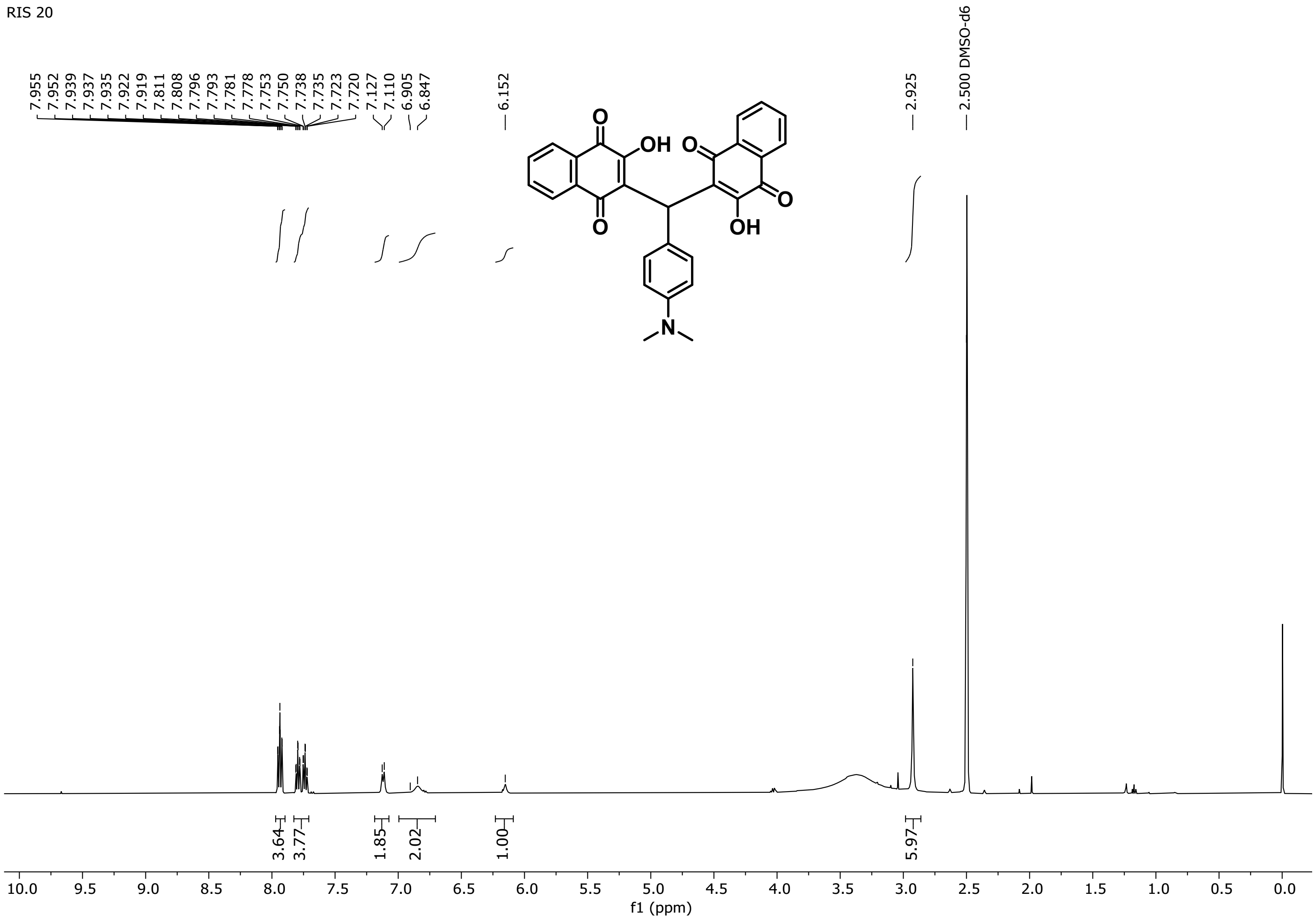

Figure S16. ^{1}H NMR spectrum of **3d** (500 MHz, DMSO-d_6).

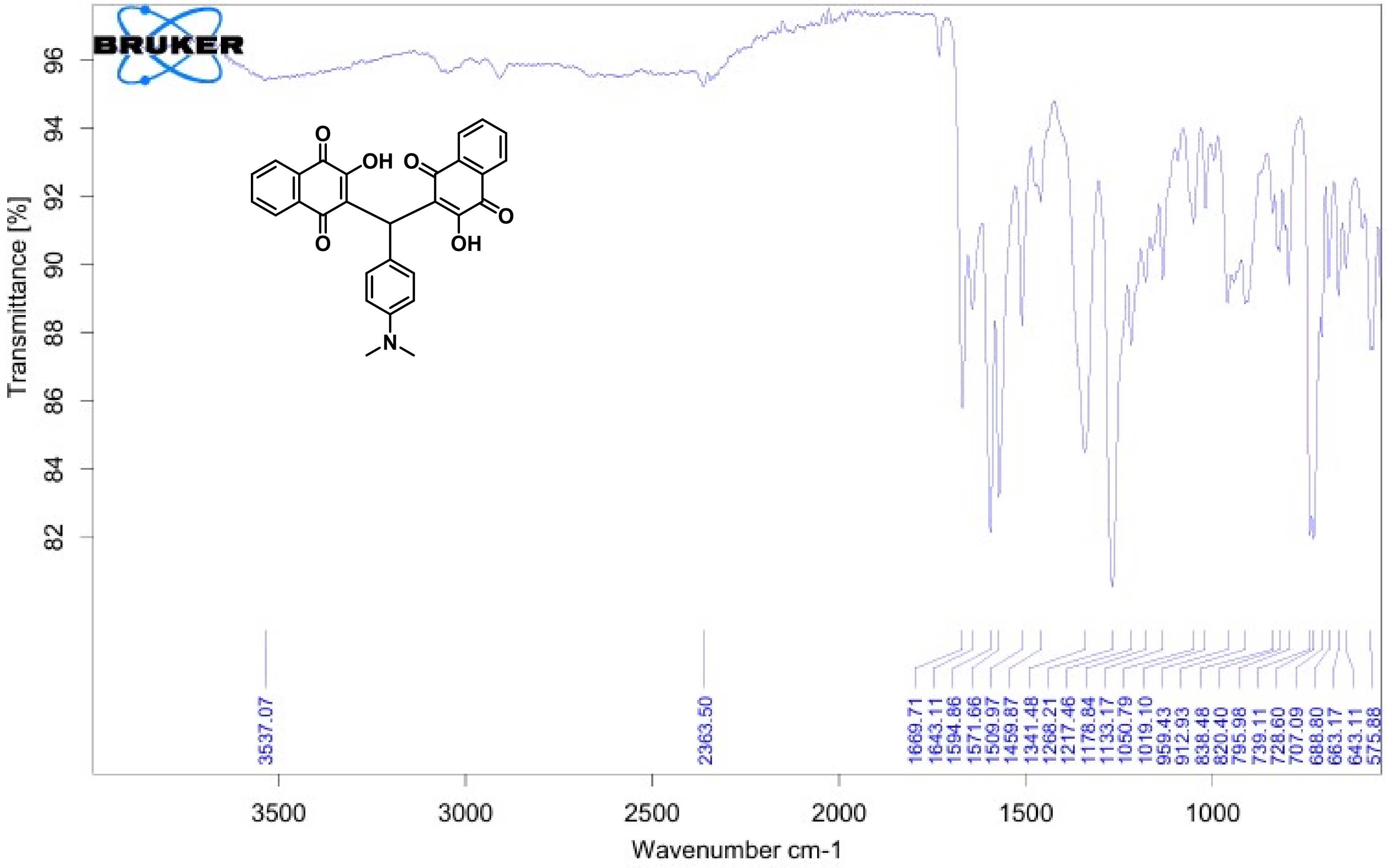

Figure S17. FT-IR spectrum of **3d**.

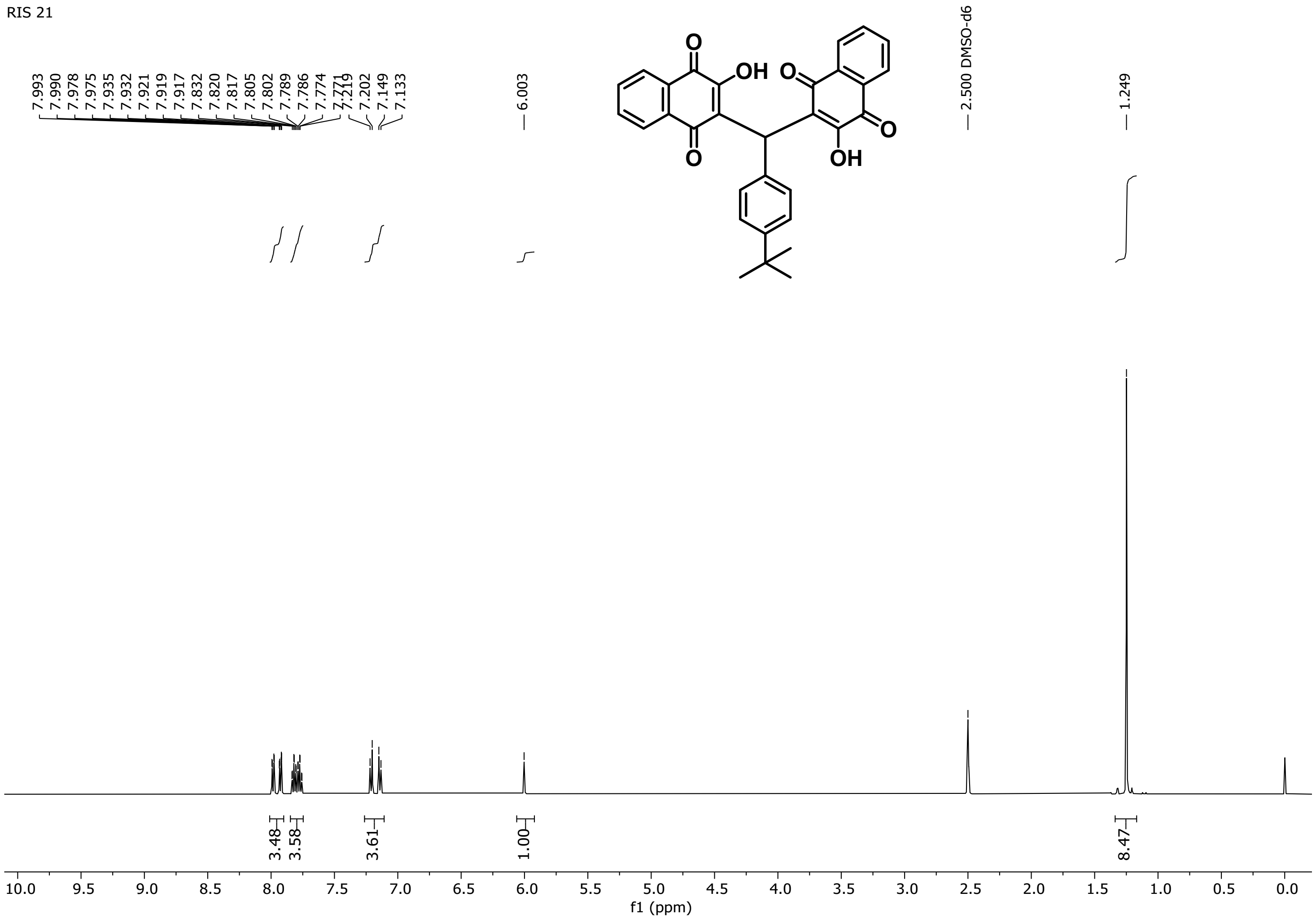

Figure S18. ^{1}H NMR spectrum of **3e** (500 MHz, DMSO-d_6).

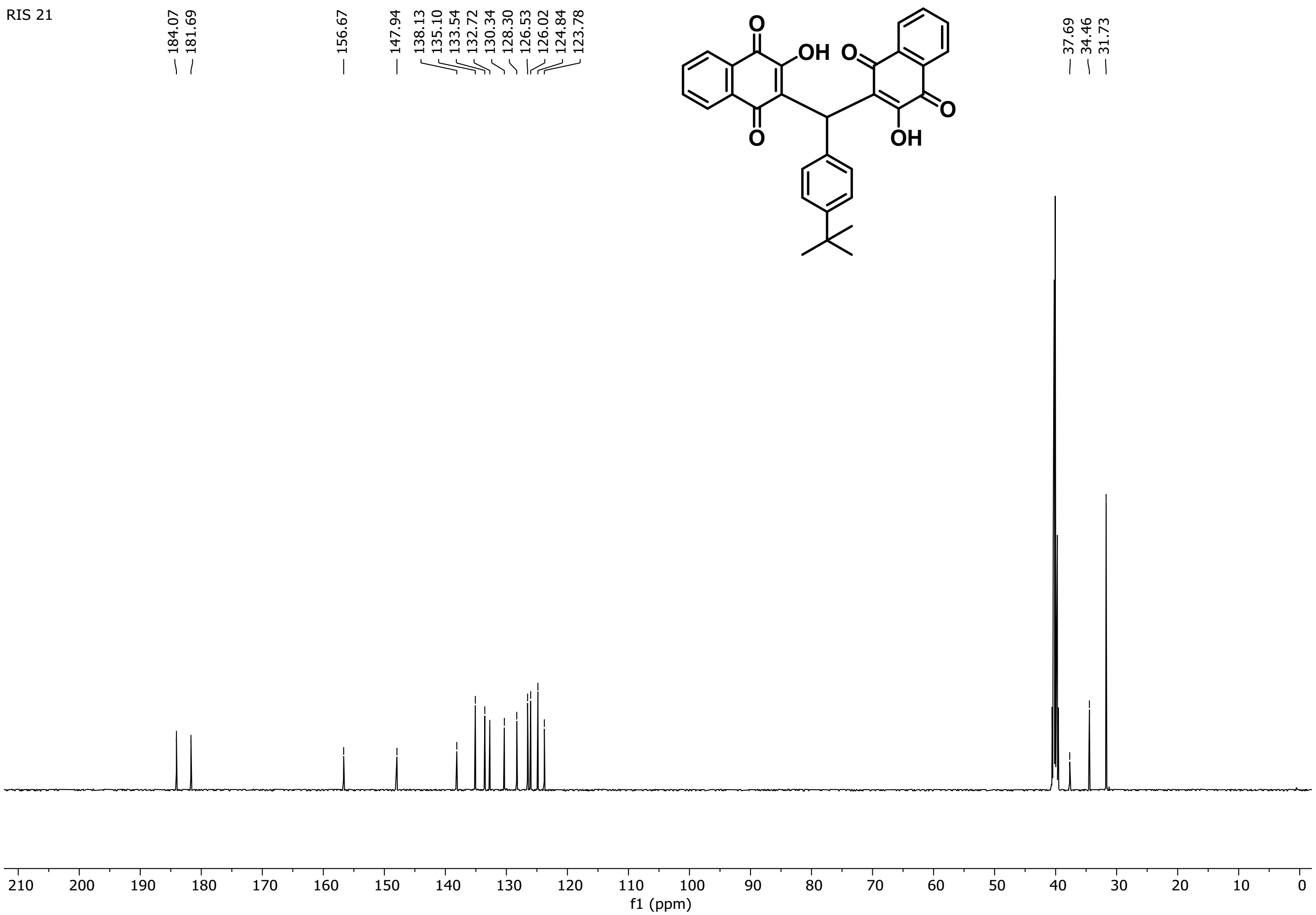

Figure S19. ^{13}C NMR spectrum of **3e** (125 MHz, DMSO-d_6).

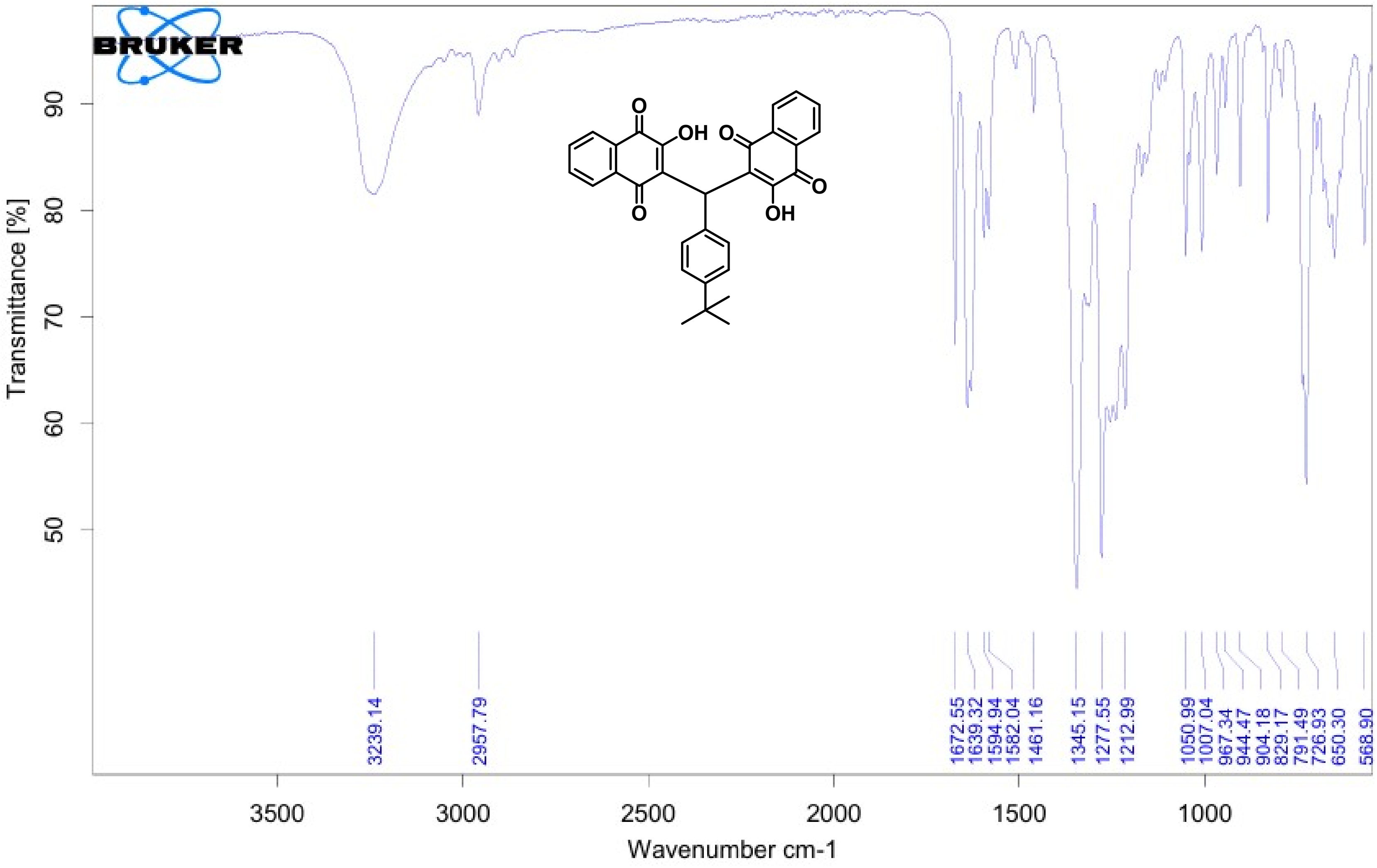

Figure S20. FT-IR spectrum of **3e**.

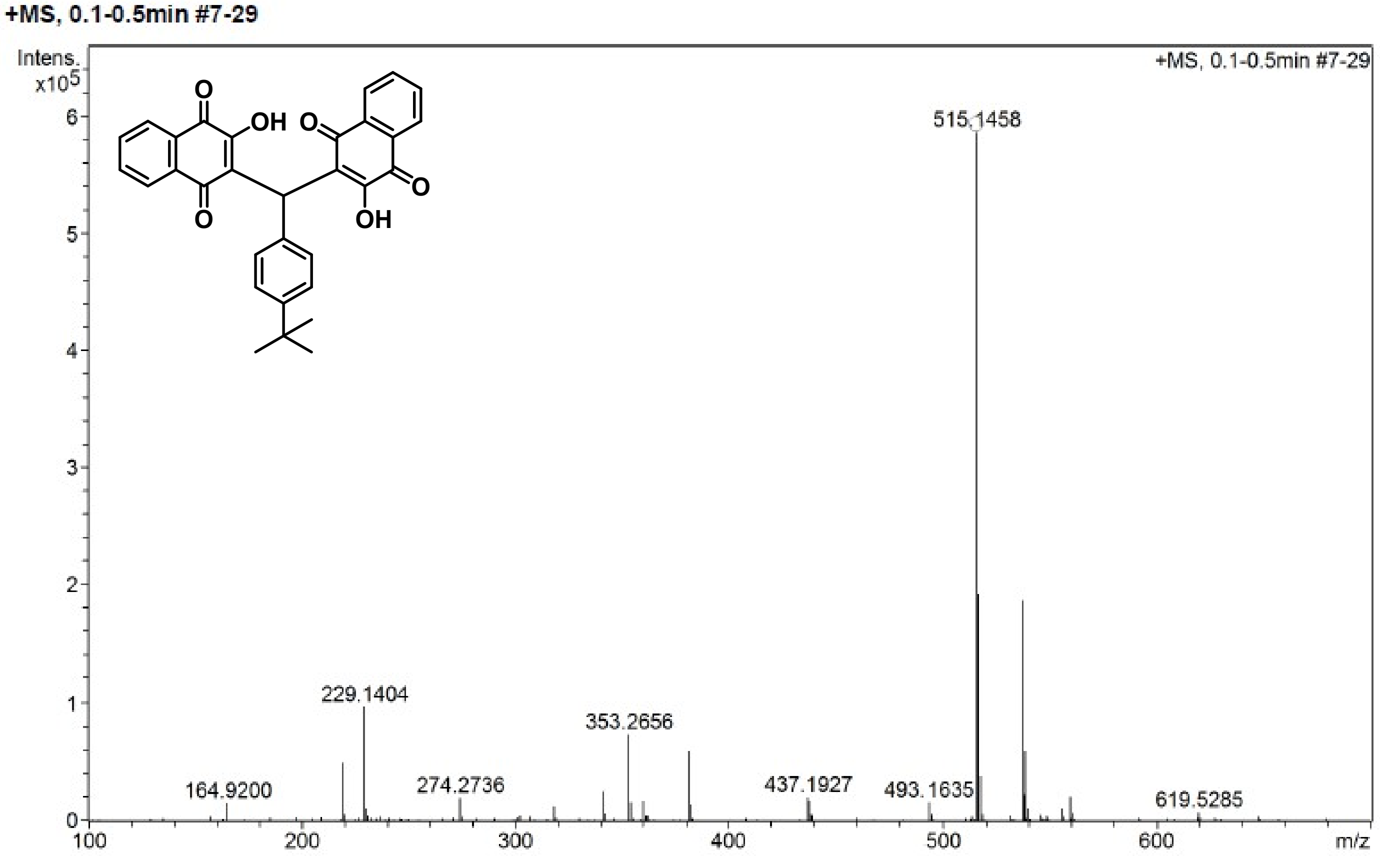

Meas. m/z	#	Ion Formula	m/z	err [ppm]	Mean err [ppm]	rdb	N-Rule	e⁻ Conf	mSigma	Std I	Std Mean m/z	Std I VarNorm	Std m/z Diff	Std Comb Dev
515.145772	1	C31H24NaO6	515.146509	1.4	1.5	19.5	ok	even	6.0	9.8	n.a.	n.a.	n.a.	n.a.
	2	C28H16N10Na	515.145161	-1.2	-1.8	25.5	ok	even	8.6	10.4	n.a.	n.a.	n.a.	n.a.

Figure S21. HRMS spectrum of **3e**.

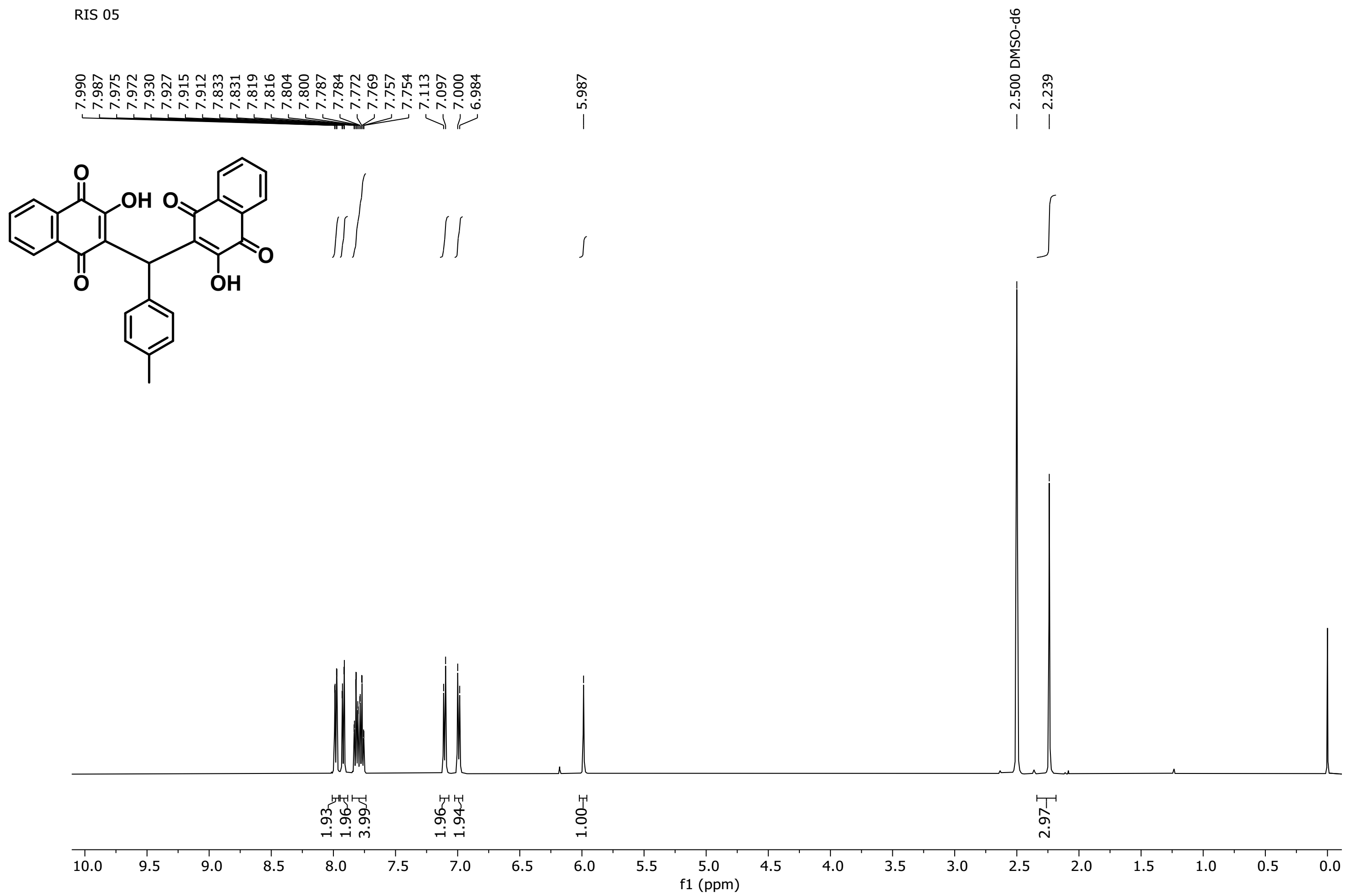

Figure S22. ^{1}H NMR spectrum of **3f** (500 MHz, DMSO-d$_6$).

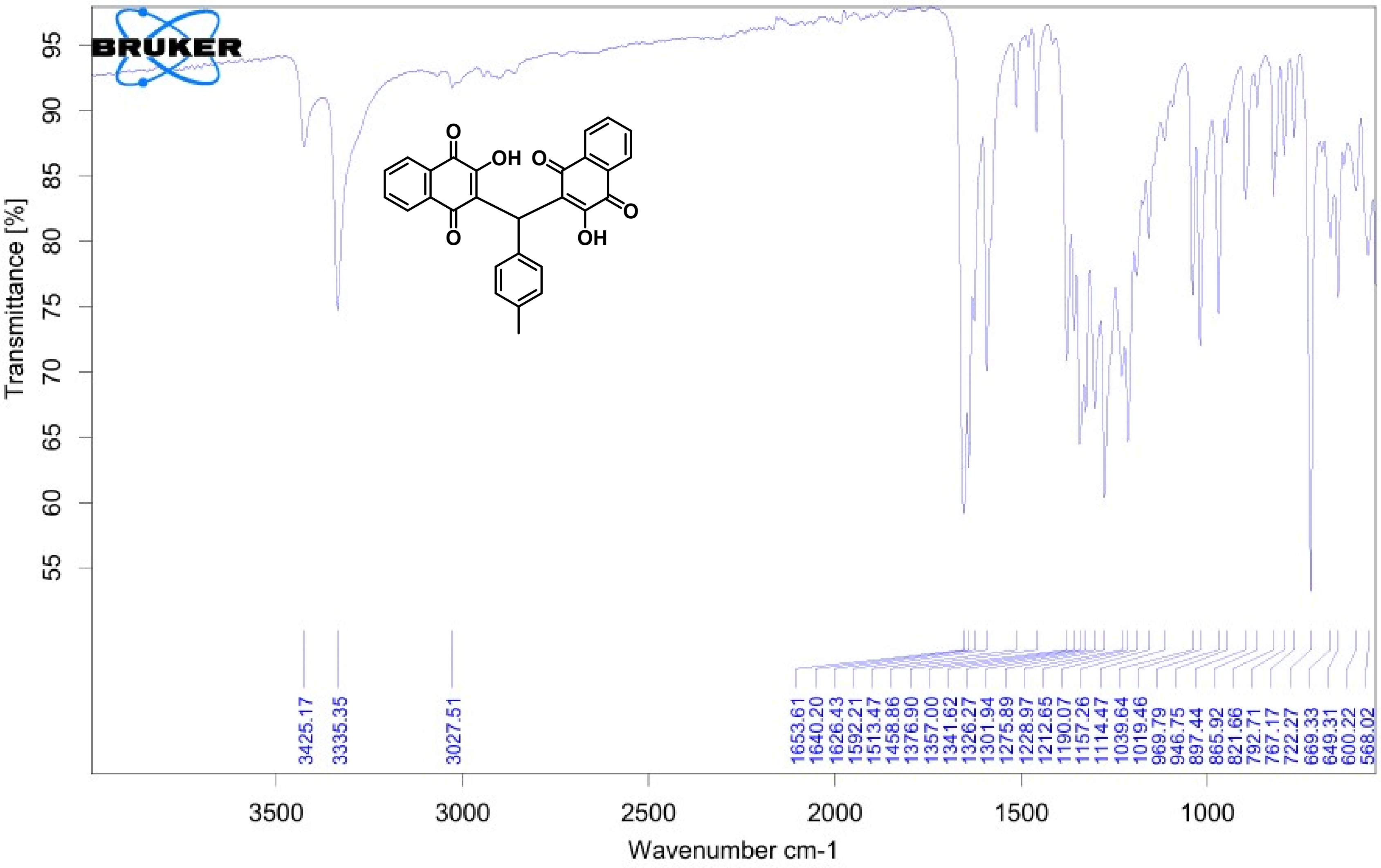

Figure S23. FT-IR spectrum of **3f**.

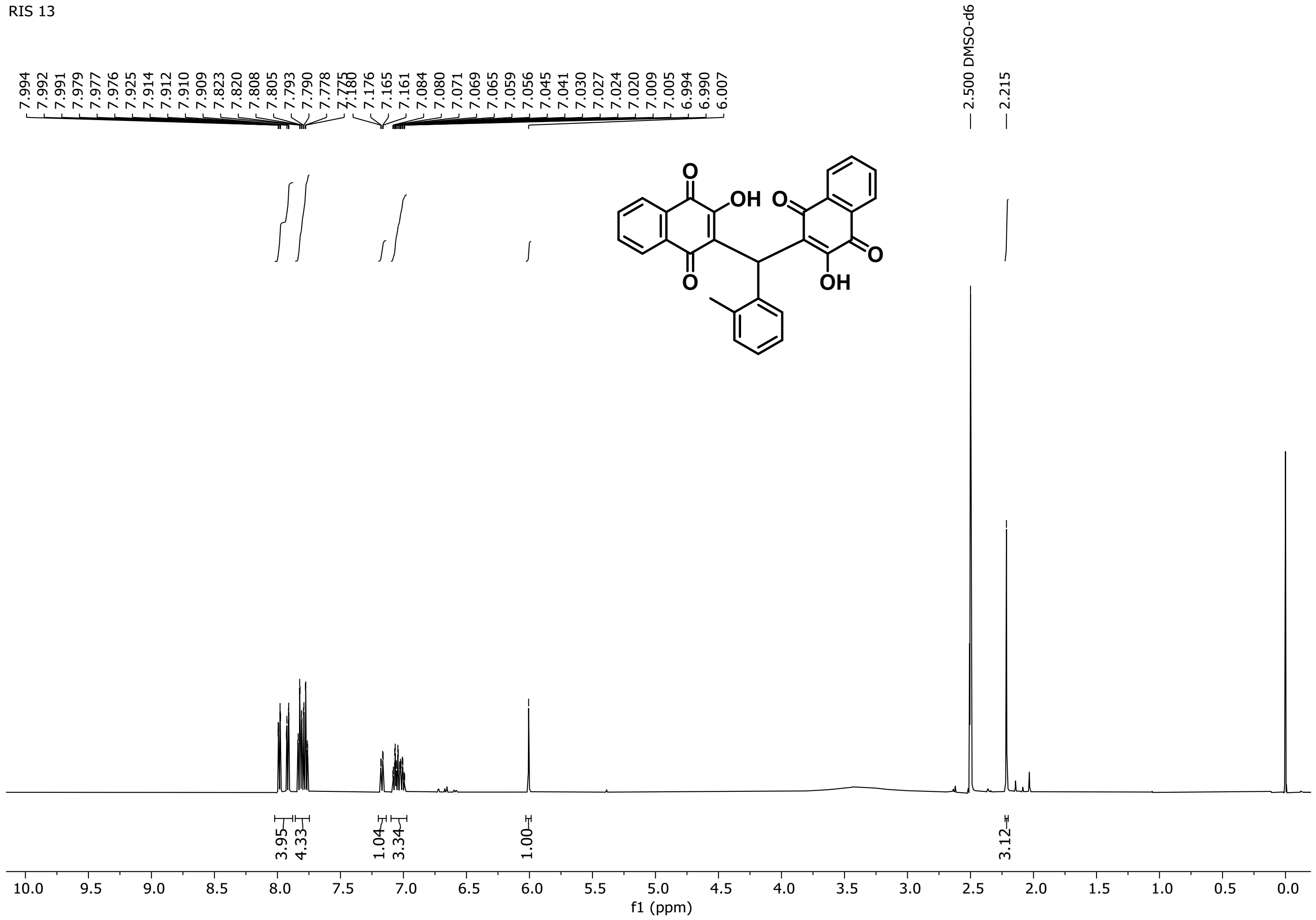

Figure S24. ^{1}H NMR spectrum of **3g** (500 MHz, DMSO-d$_6$).

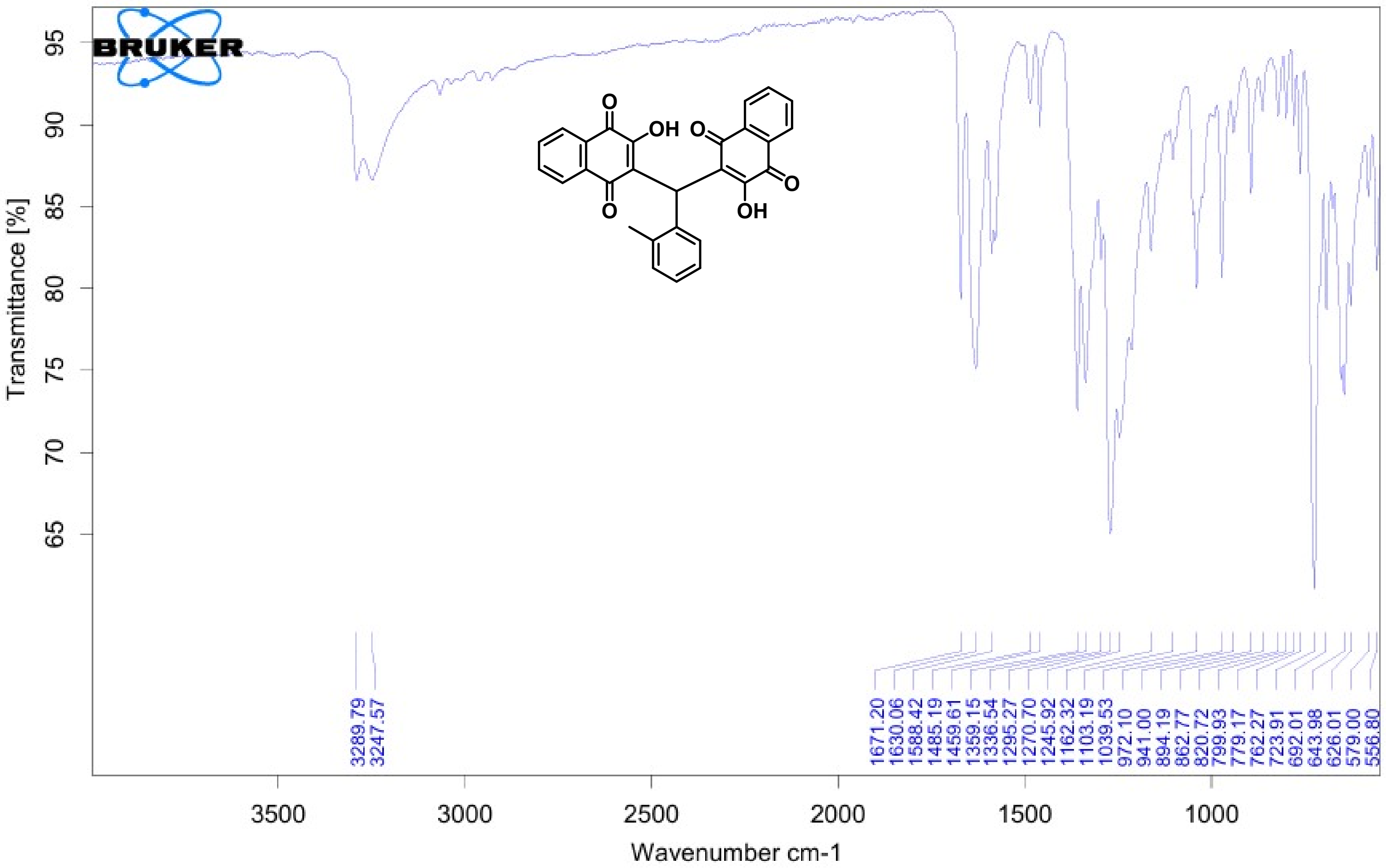

Figure S25. FT-IR spectrum of **3g**.

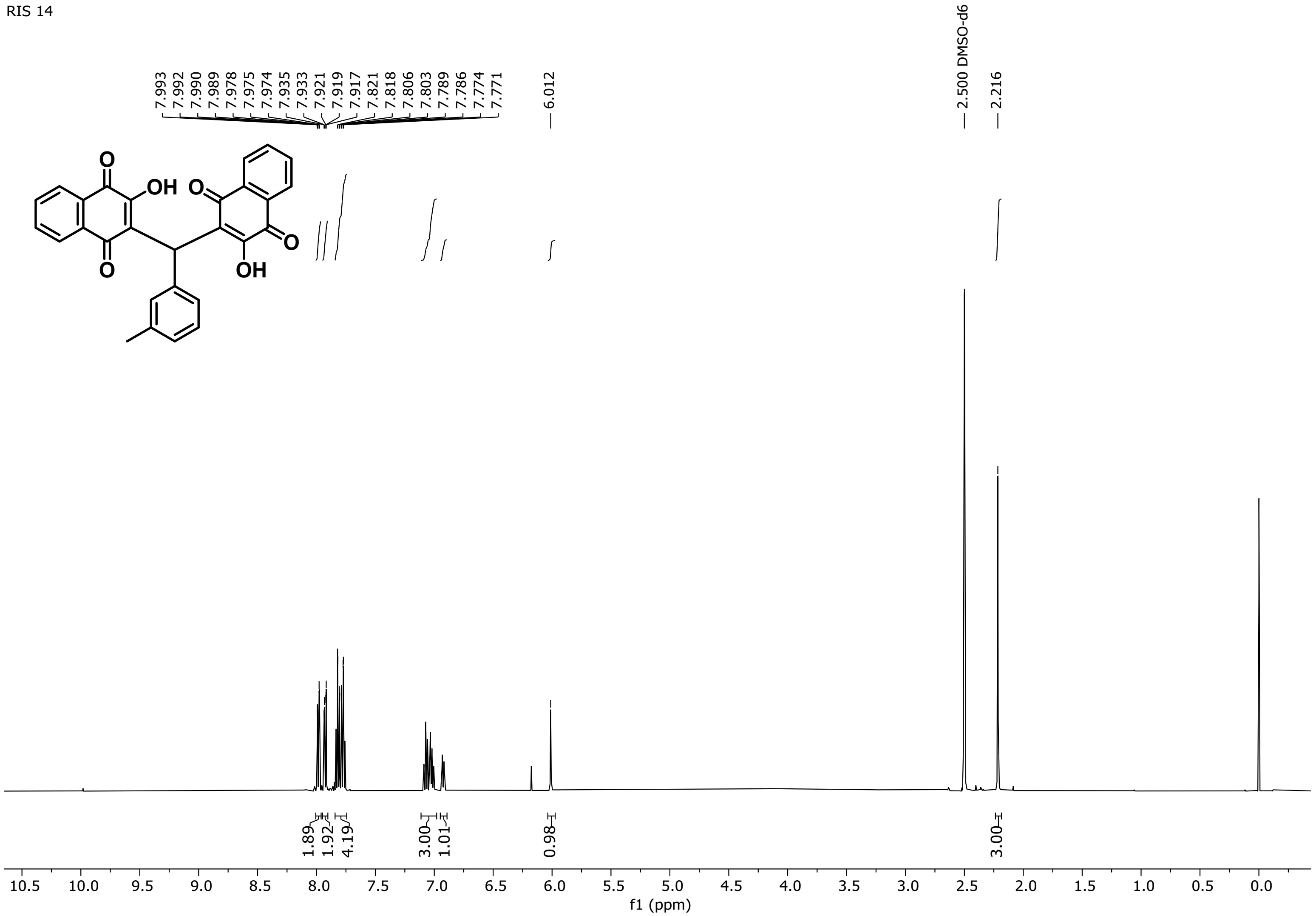

Figure S26. ^{1}H NMR spectrum of **3h** (500 MHz, DMSO-d_6).

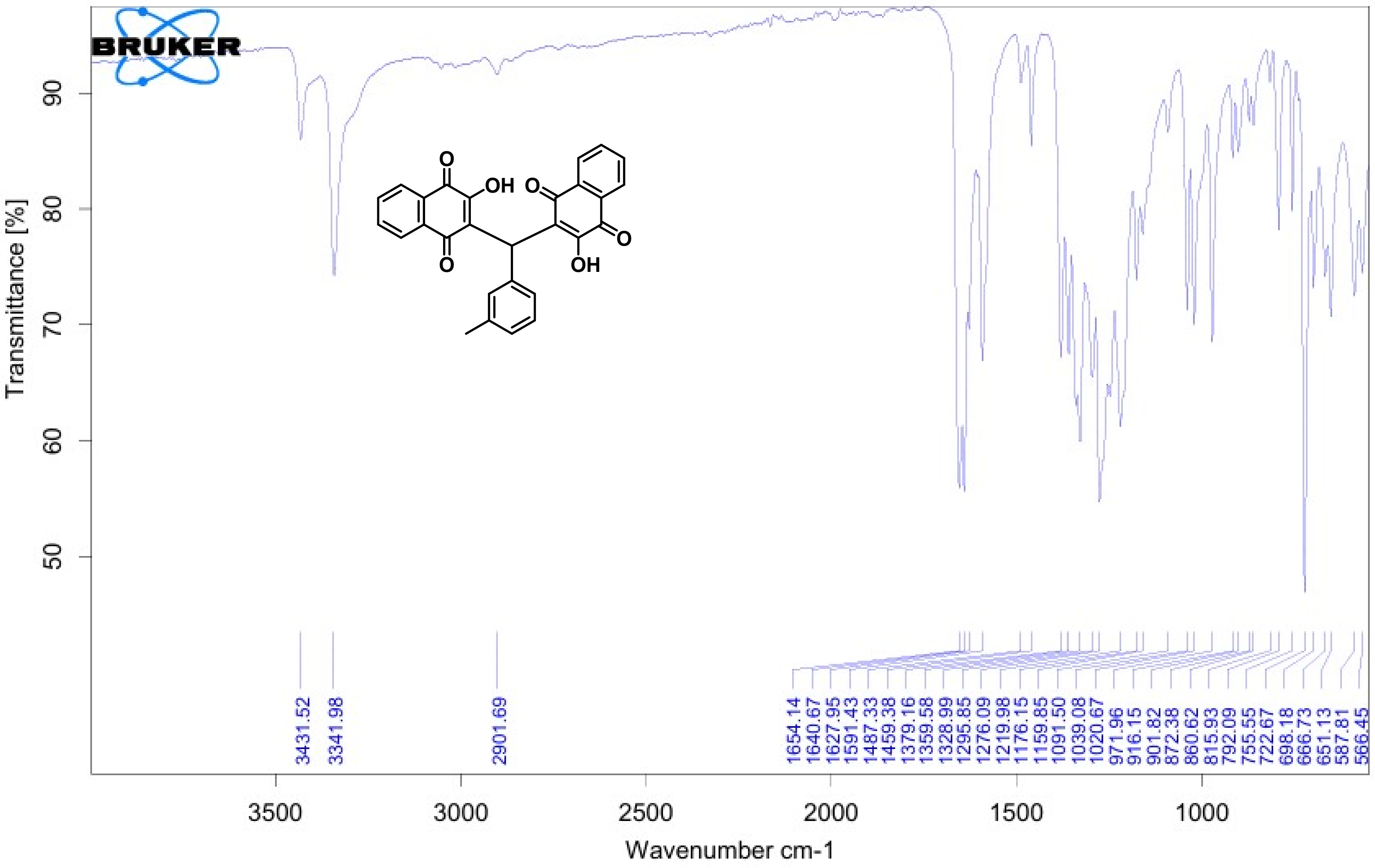

Figure S27. FT-IR spectrum of **3h**.

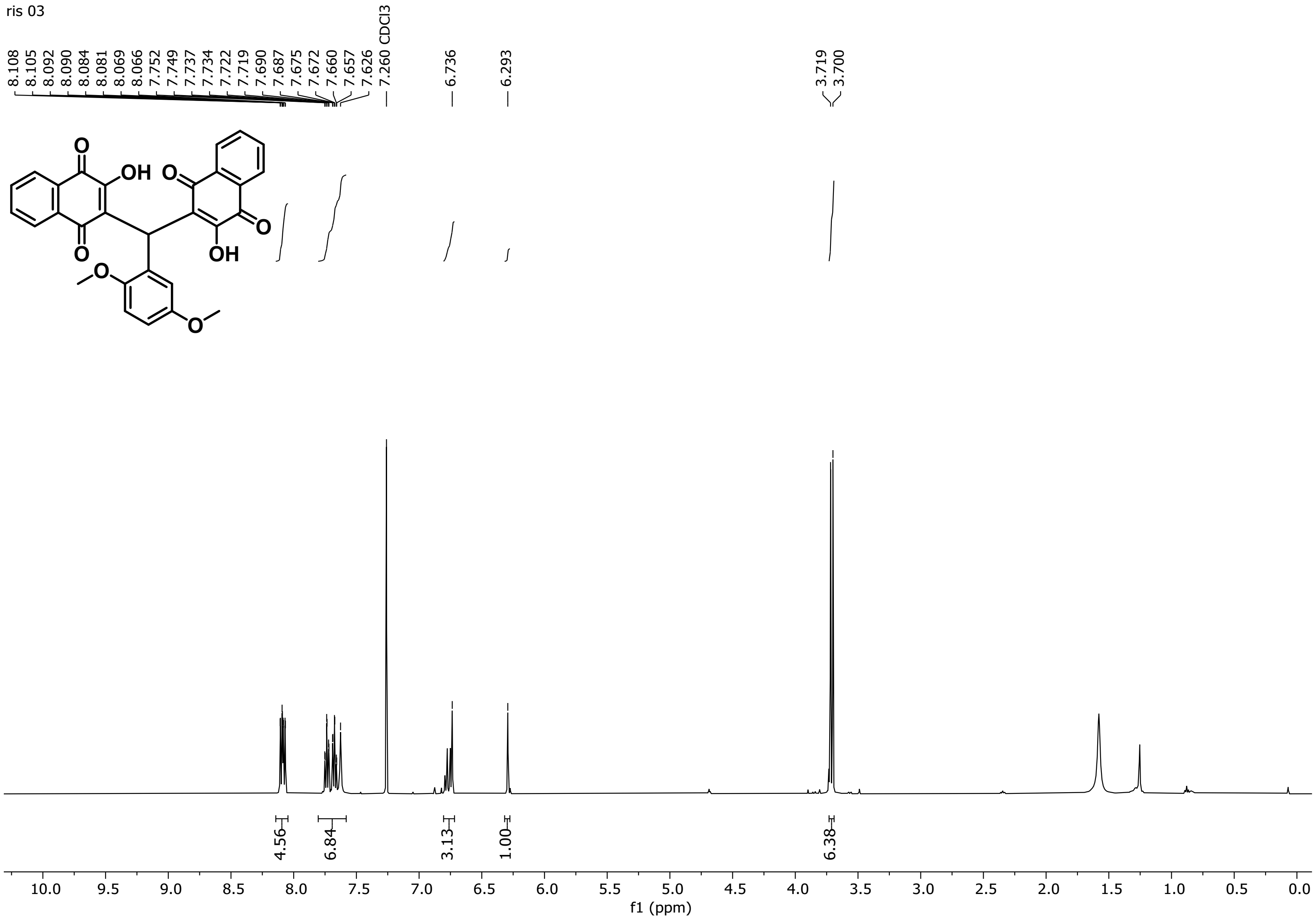

Figure S28. ^{1}H NMR spectrum of **3h** (500 MHz, DMSO-d$_6$).

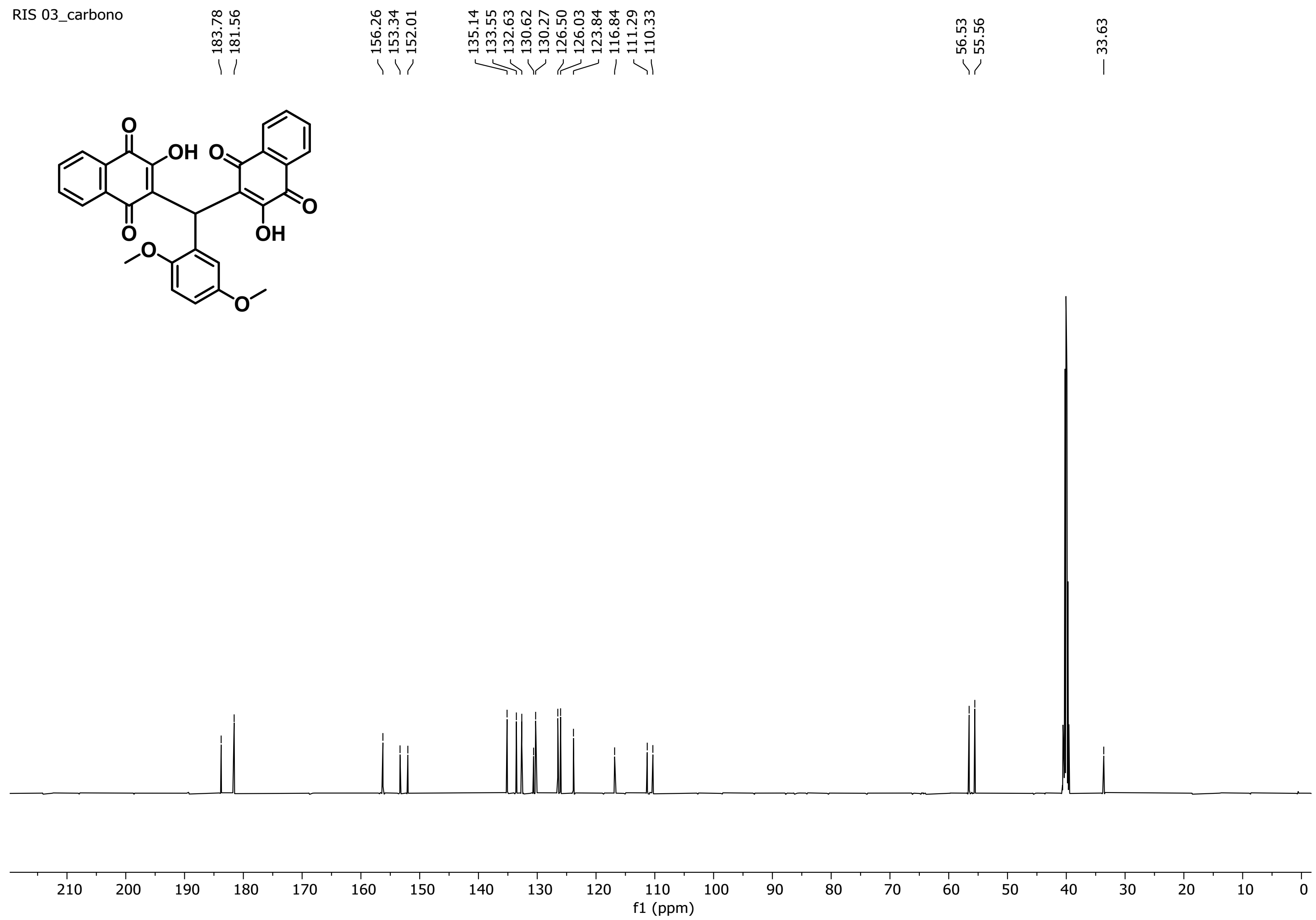

Figure S29. ^{13}C NMR spectrum of **3i** (125 MHz, DMSO-d_6).

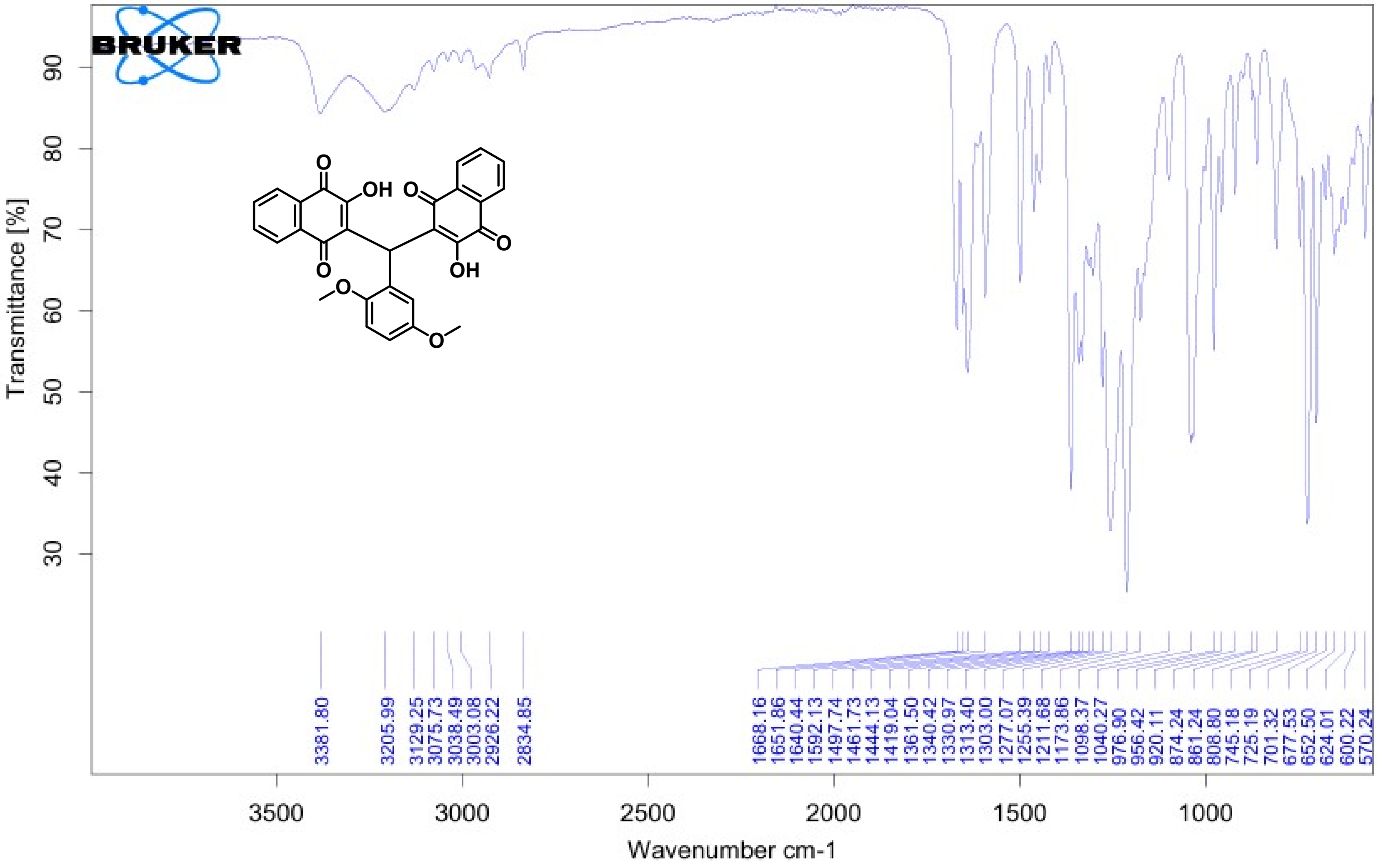

Figure S30. FT-IR spectrum of **3i**.

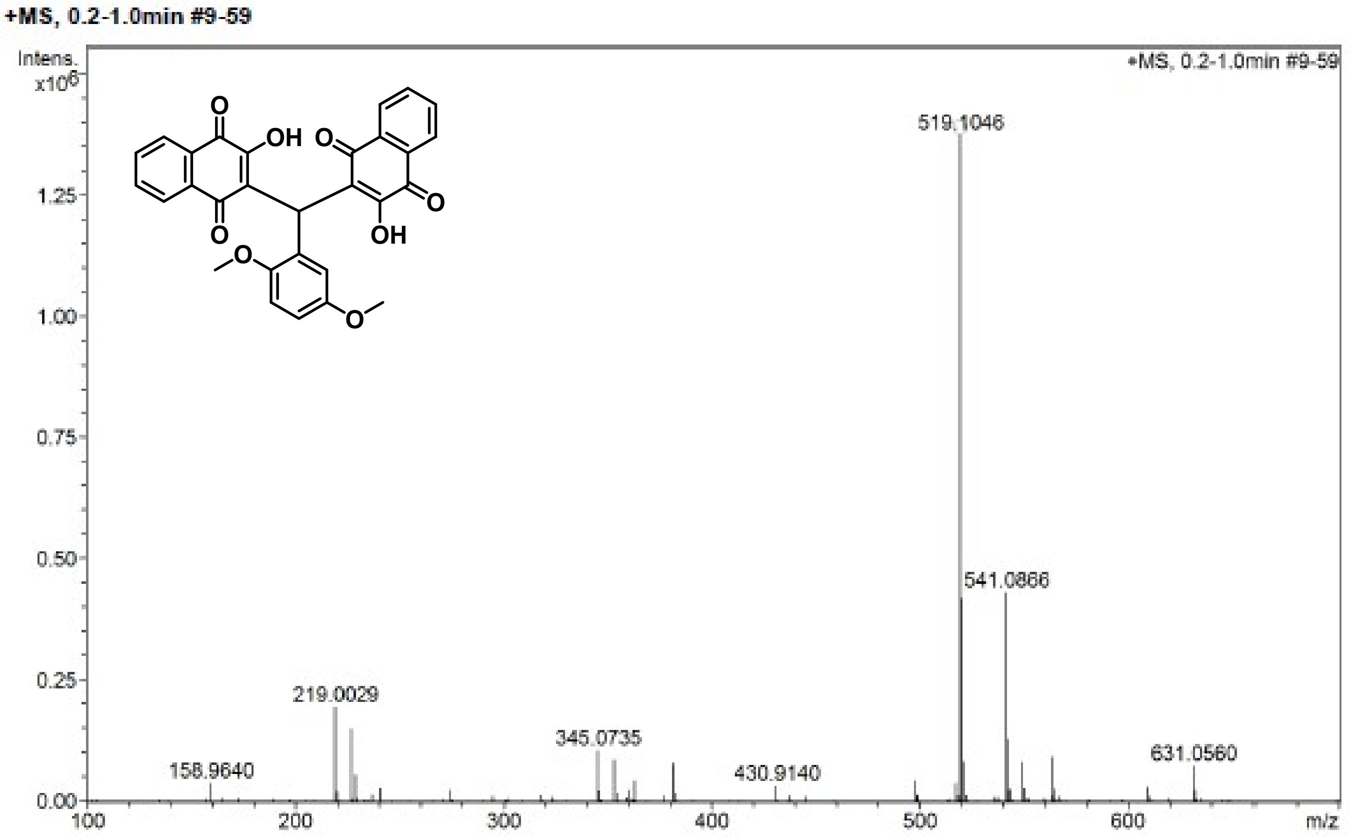

Meas. m/z	#	Ion Formula	m/z	err [ppm]	Mean err [ppm]	rdb	N-Rule	e⁻ Conf	mSigma	Std I	Std Mean m/z	Std I VarNorm	Std m/z Diff	Std Comb Dev
519.104551	1	C29H20NaO8	519.105038	0.9	1.1	19.5	ok	even	7.5	12.8	n.a.	n.a.	n.a.	n.a.
	2	C26H12N10NaO2	519.103690	-1.7	-2.3	25.5	ok	even	9.5	11.7	n.a.	n.a.	n.a.	n.a.

Figure S31. HRMS spectrum of **3i**.

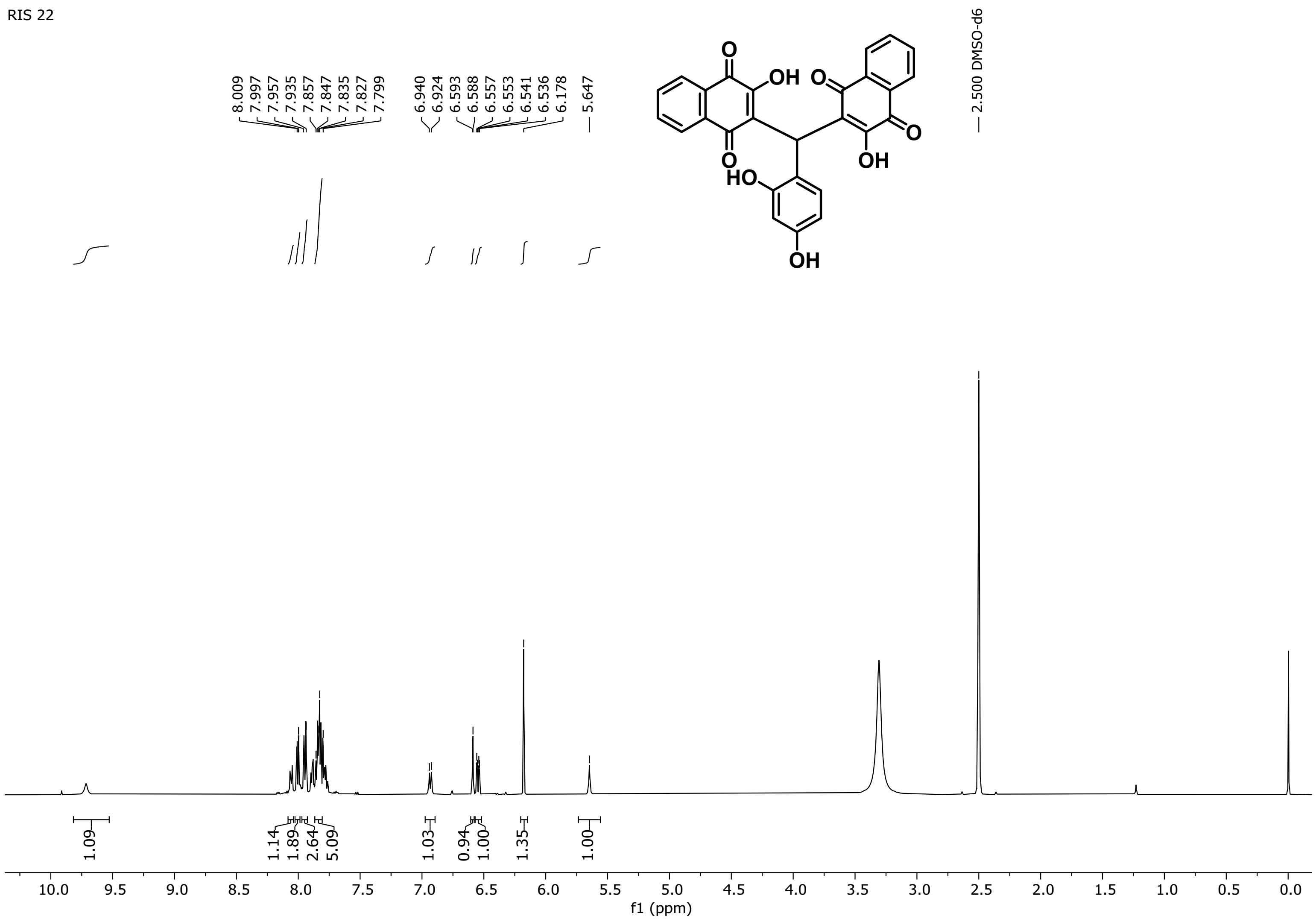

Figure S32. ^{1}H NMR spectrum of **3j** (500 MHz, DMSO-d$_6$).

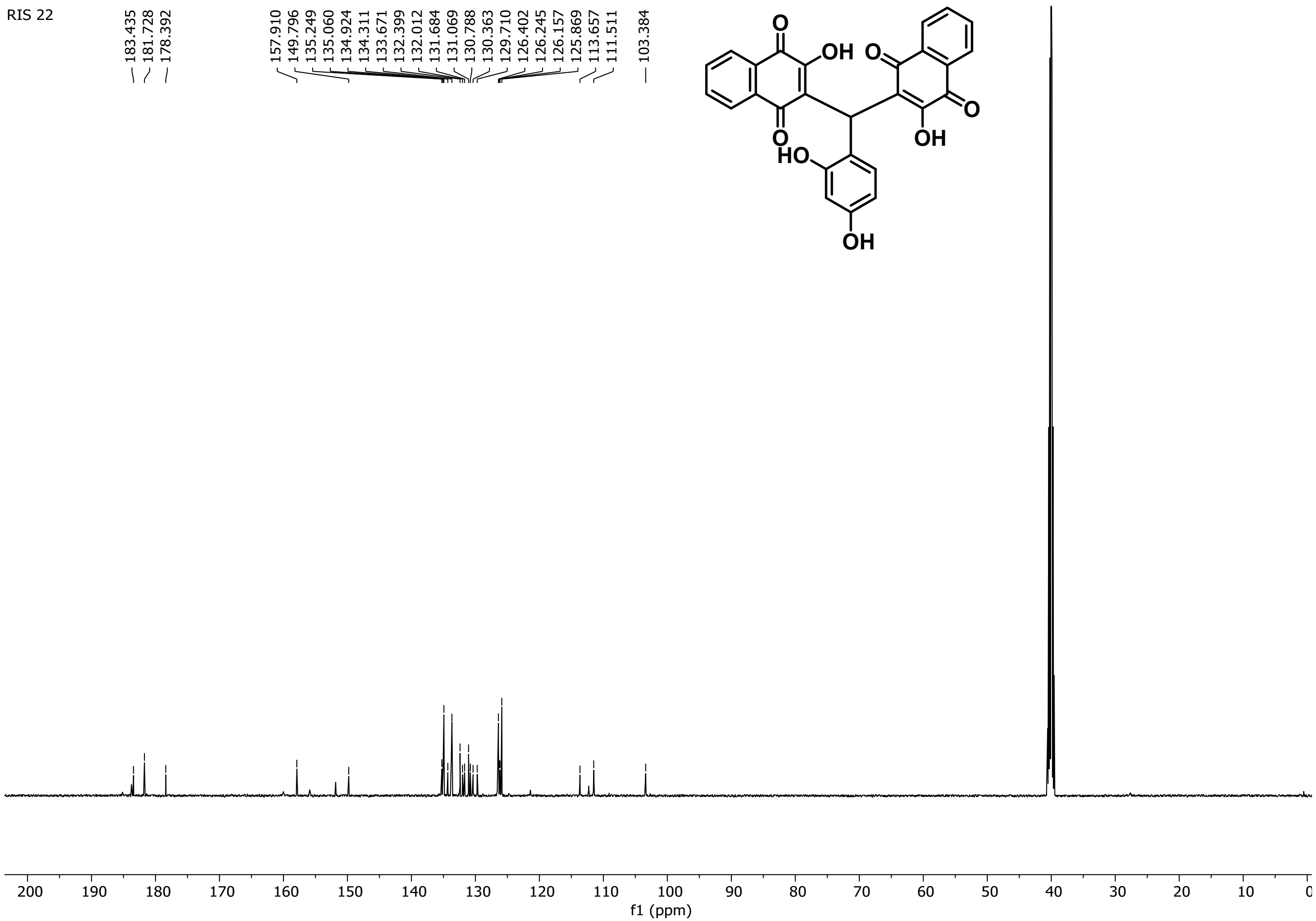

Figure S33. ^{13}C NMR spectrum of **3j** (125 MHz, DMSO-d$_6$).

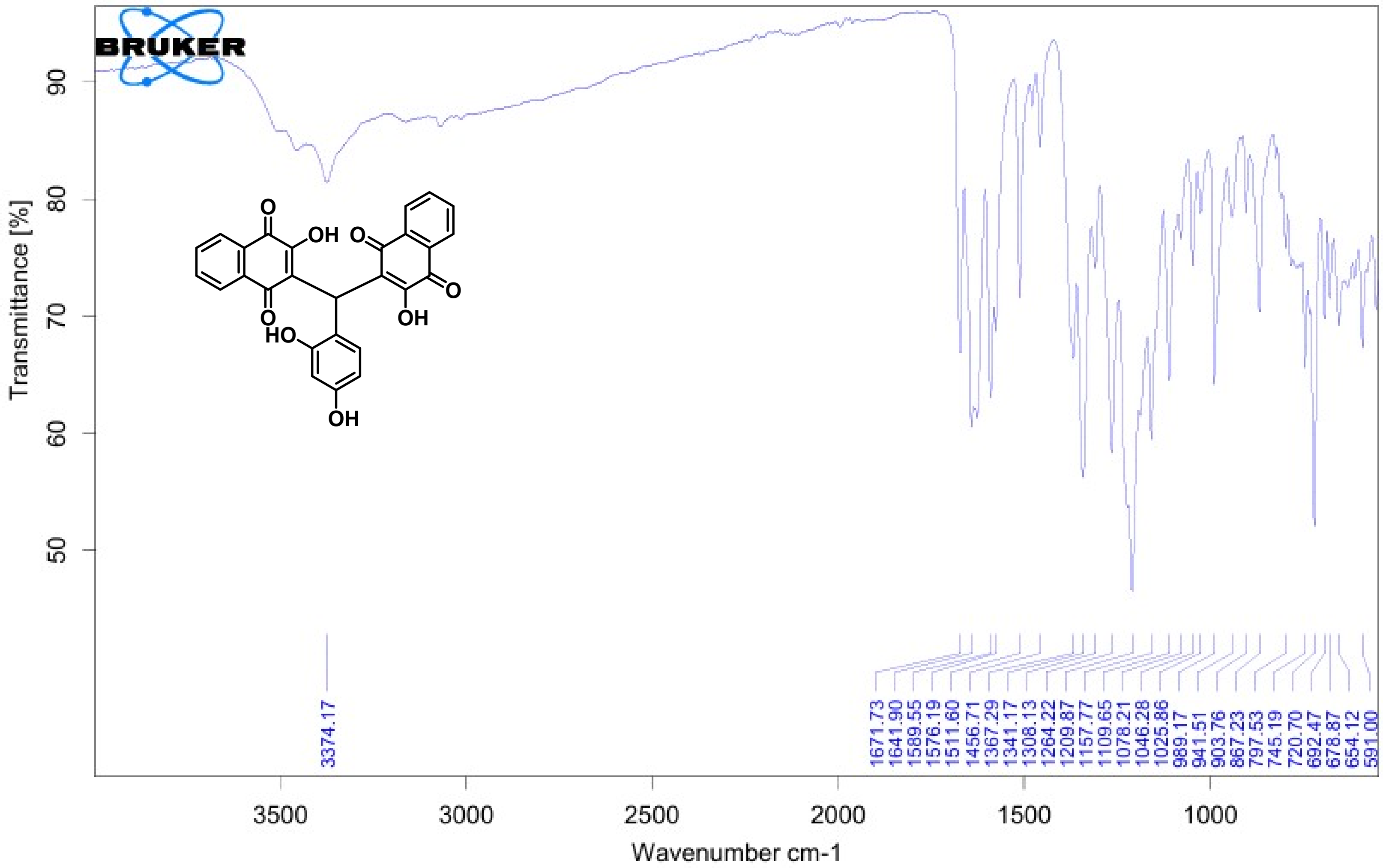

Figure S34. FT-IR spectrum of **3j**.

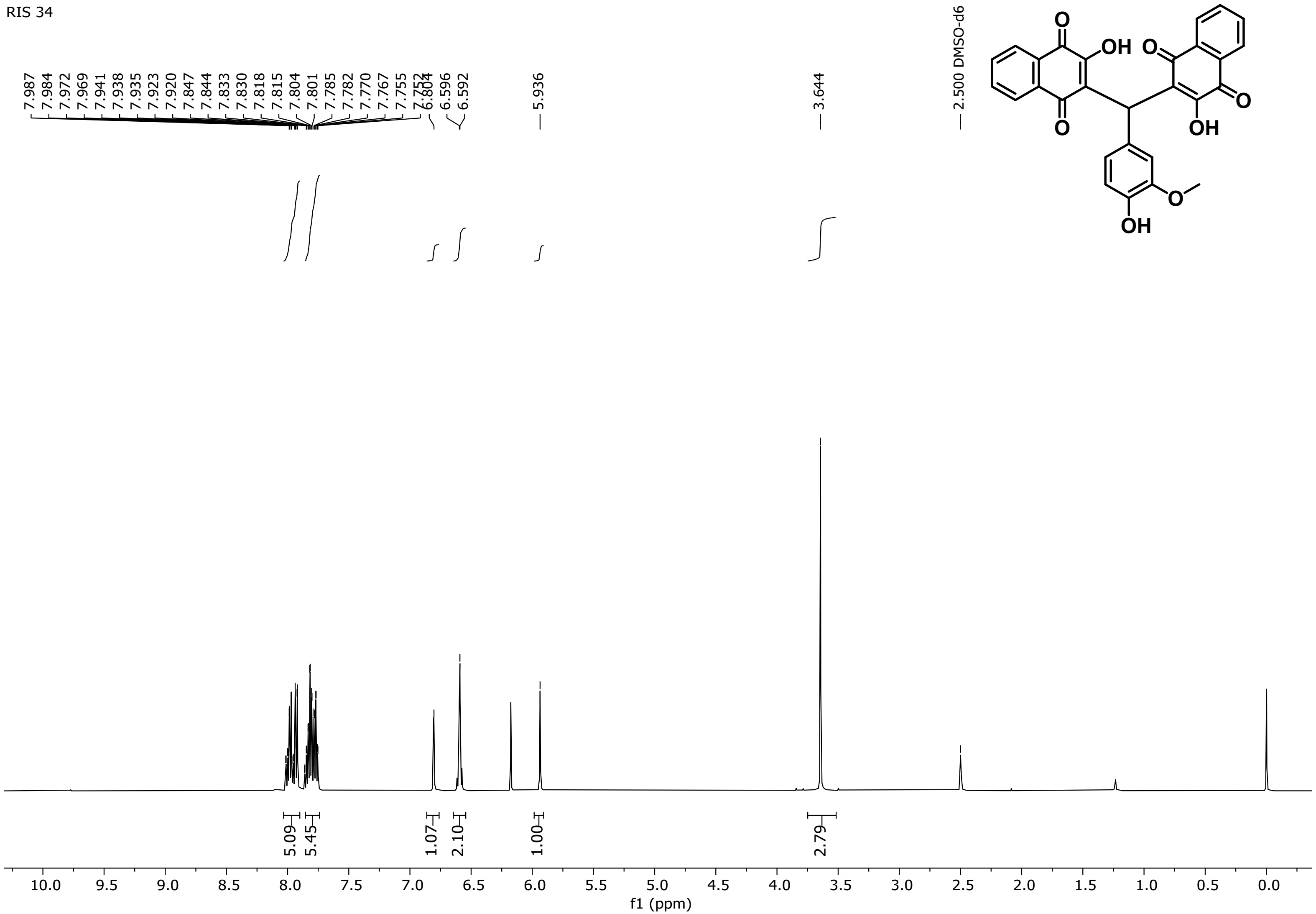

Figure S35. ${}^{1}H$ NMR spectrum of **3k** (500 MHz, DMSO-d_6).

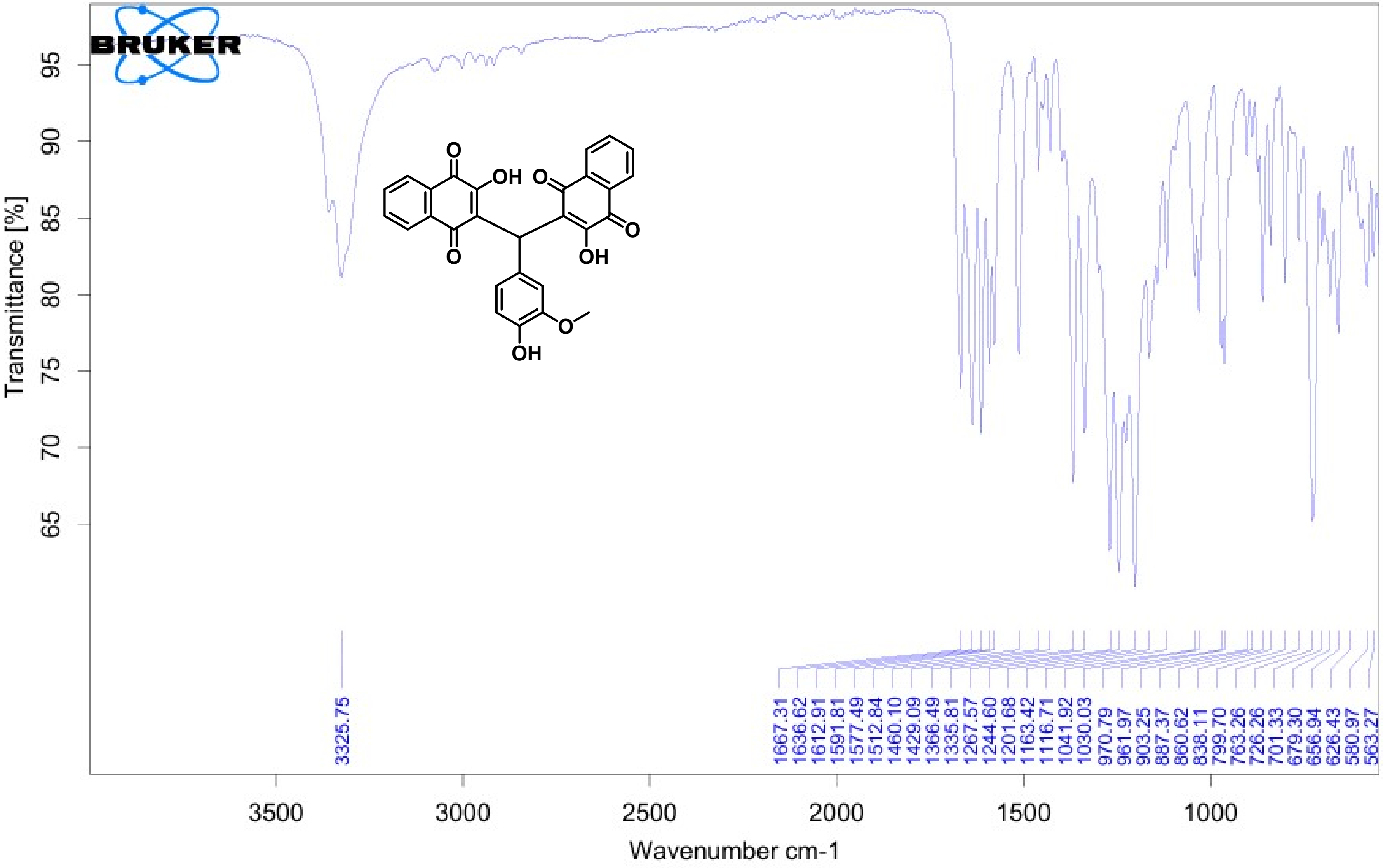

Figure S36. FT-IR spectrum of **3k**.

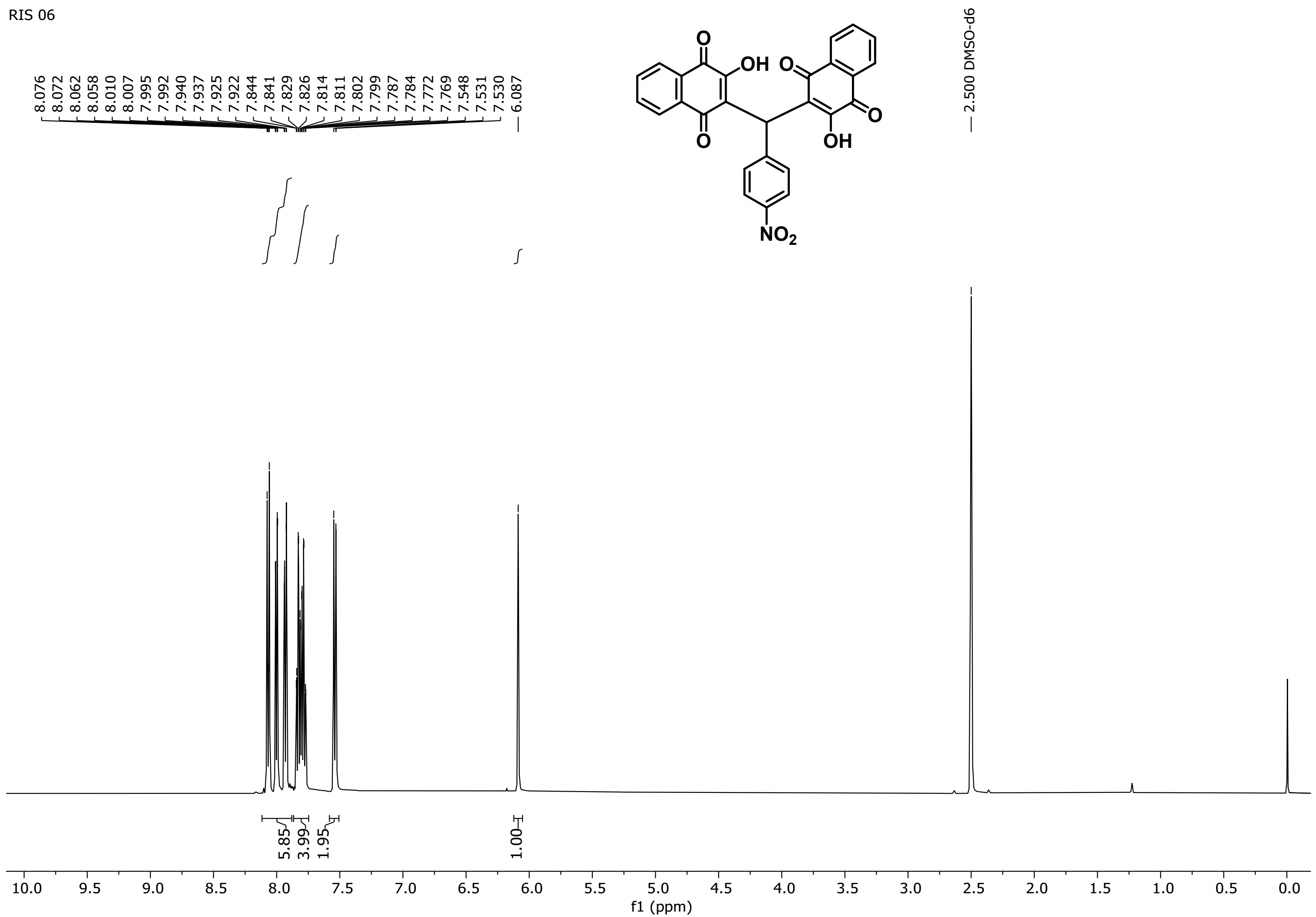

Figure S37. ¹H NMR spectrum of **3l** (500 MHz, DMSO-d_6).

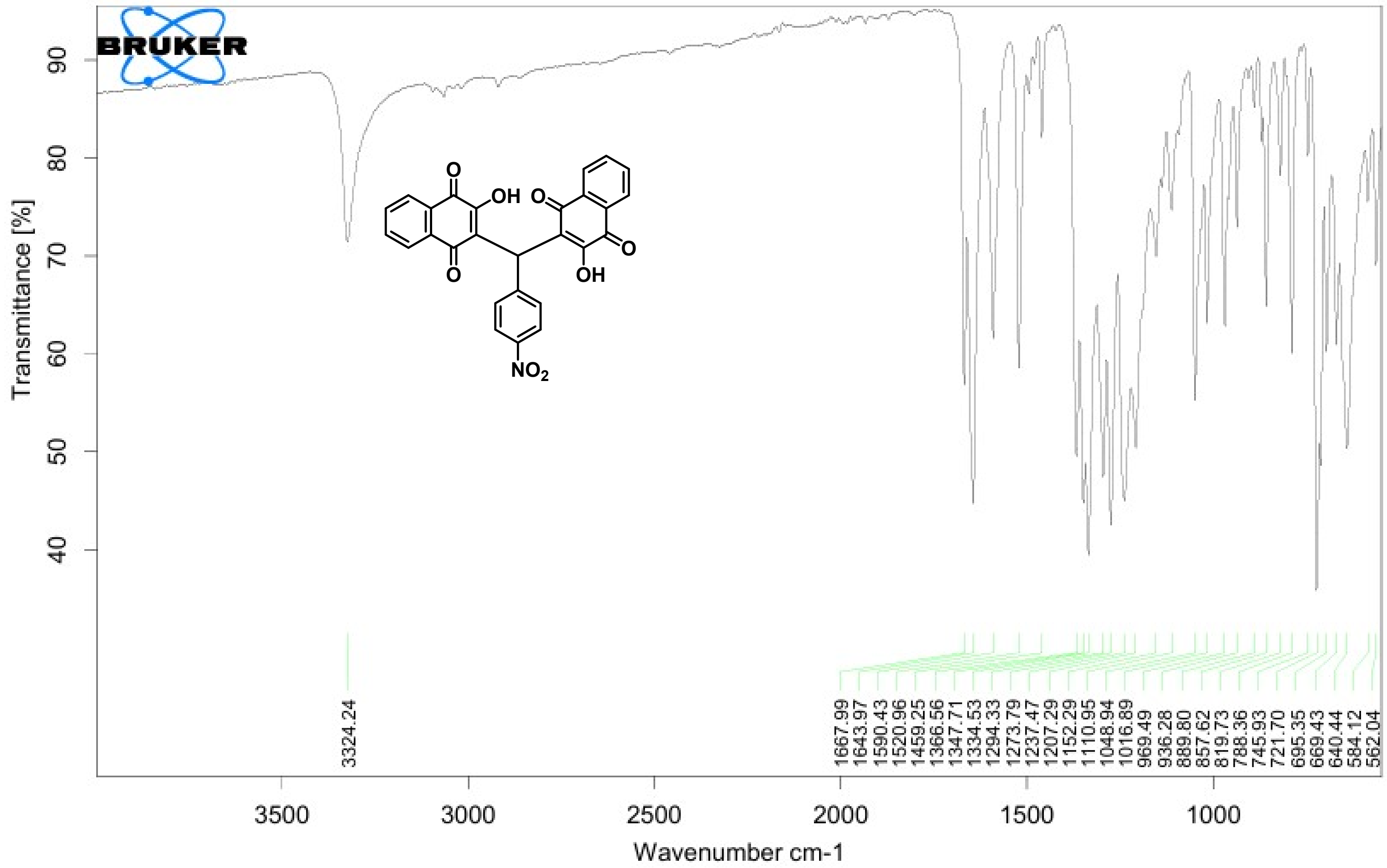

Figure S38. FT-IR spectrum of **3l**.

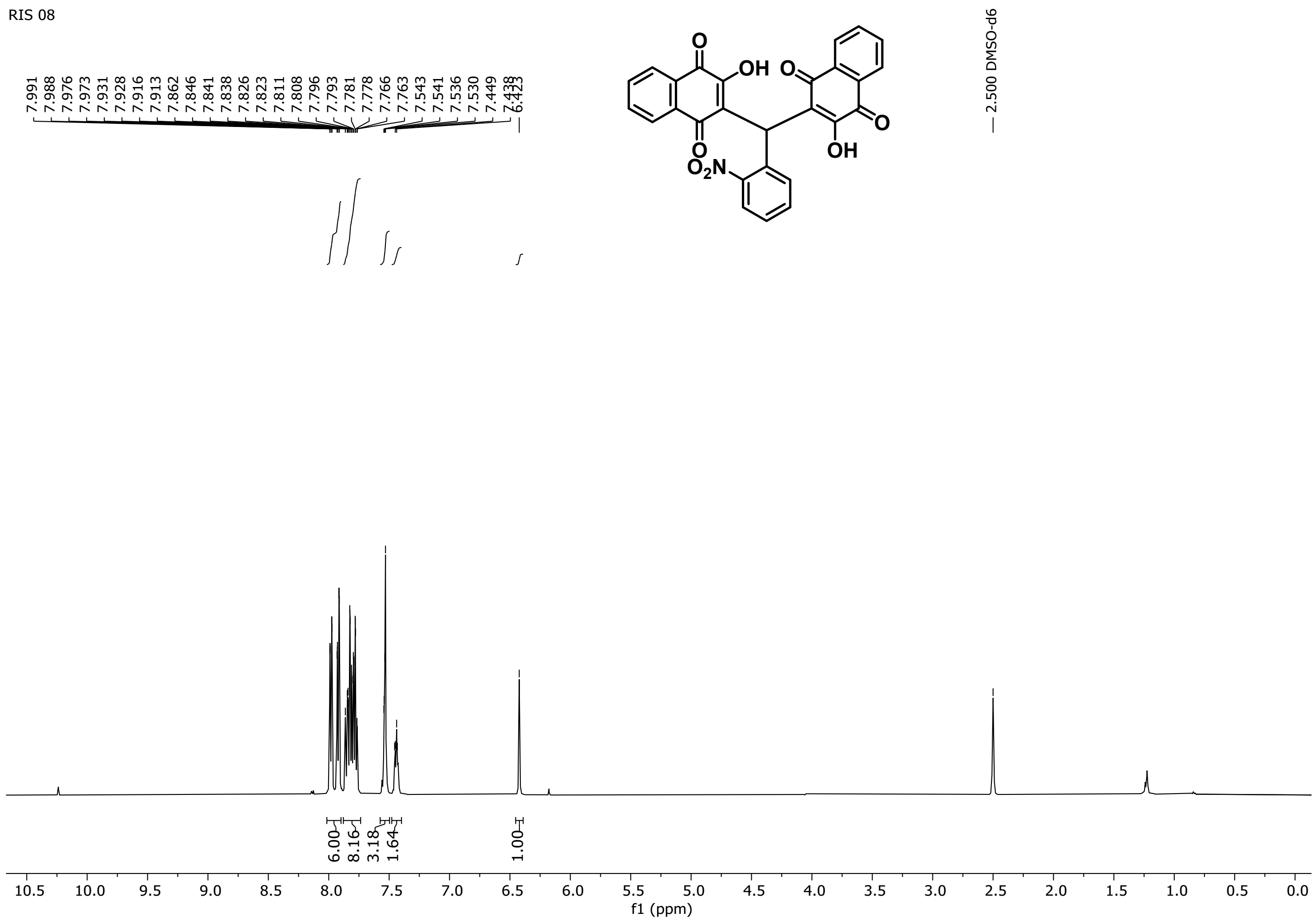

Figure S39. ^{1}H NMR spectrum of **3m** (500 MHz, DMSO-d_6).

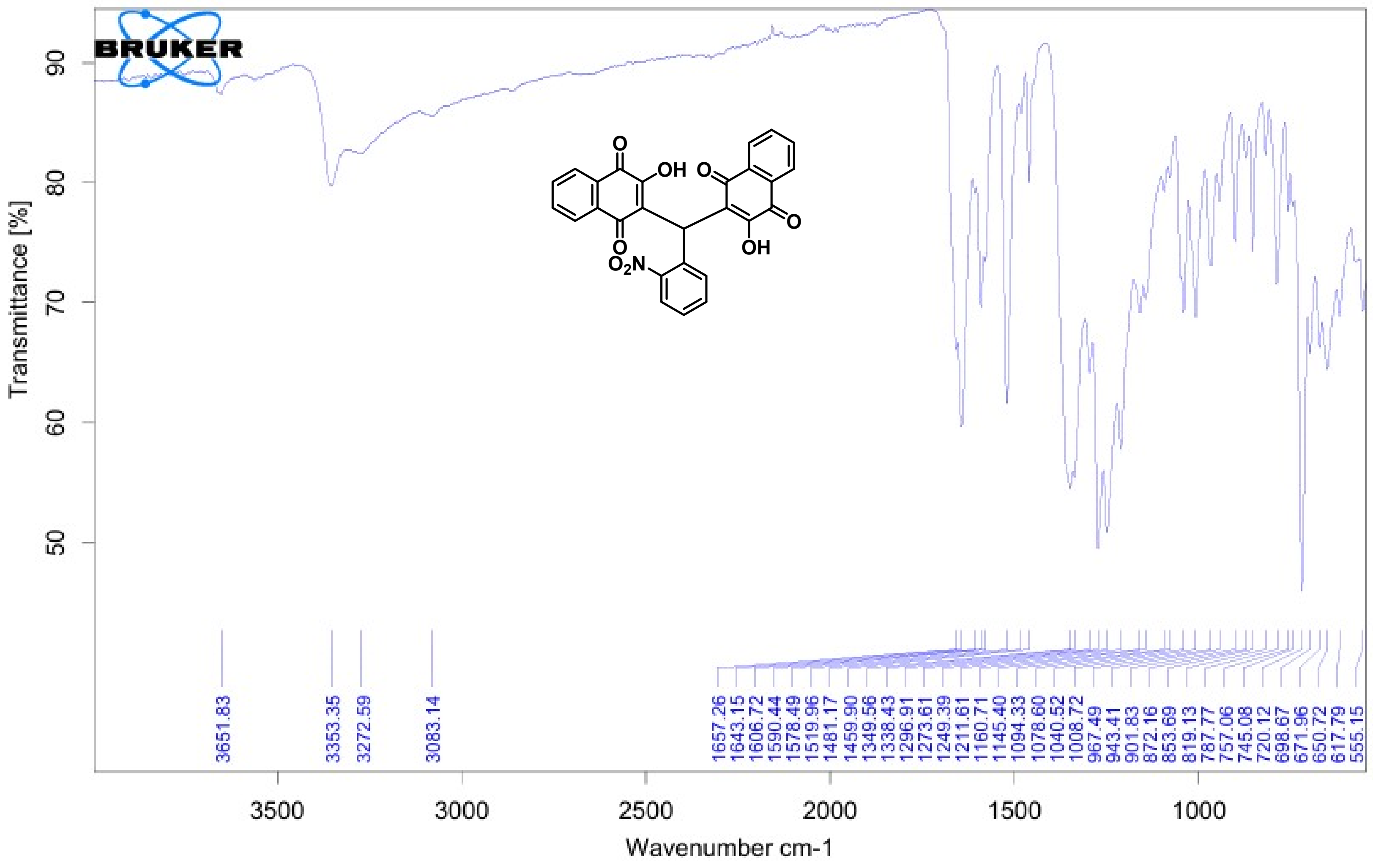

Figure S40. FT-IR spectrum of **3m**.

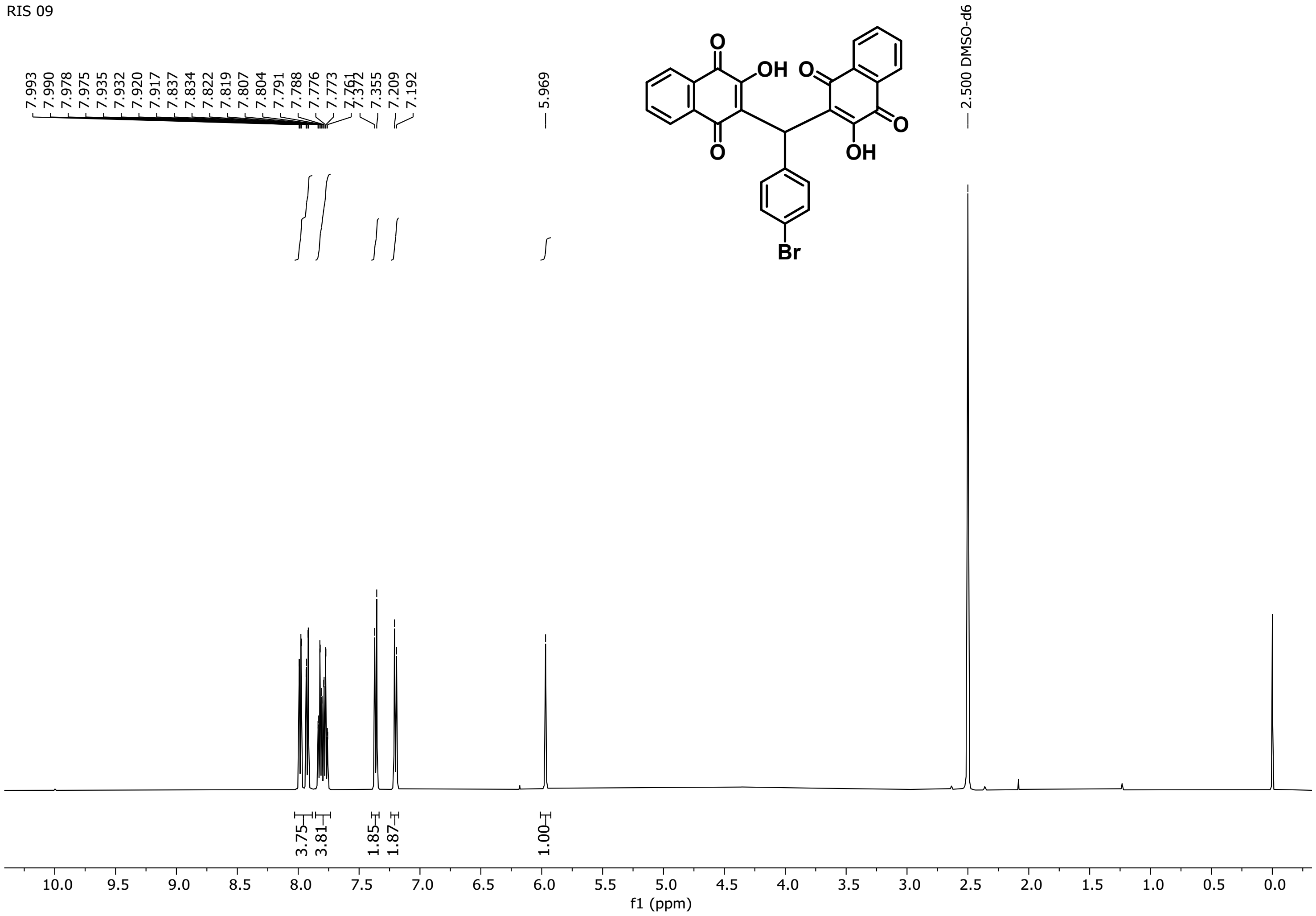

Figure S41. ^{1}H NMR spectrum of **3n** (500 MHz, DMSO-d$_6$).

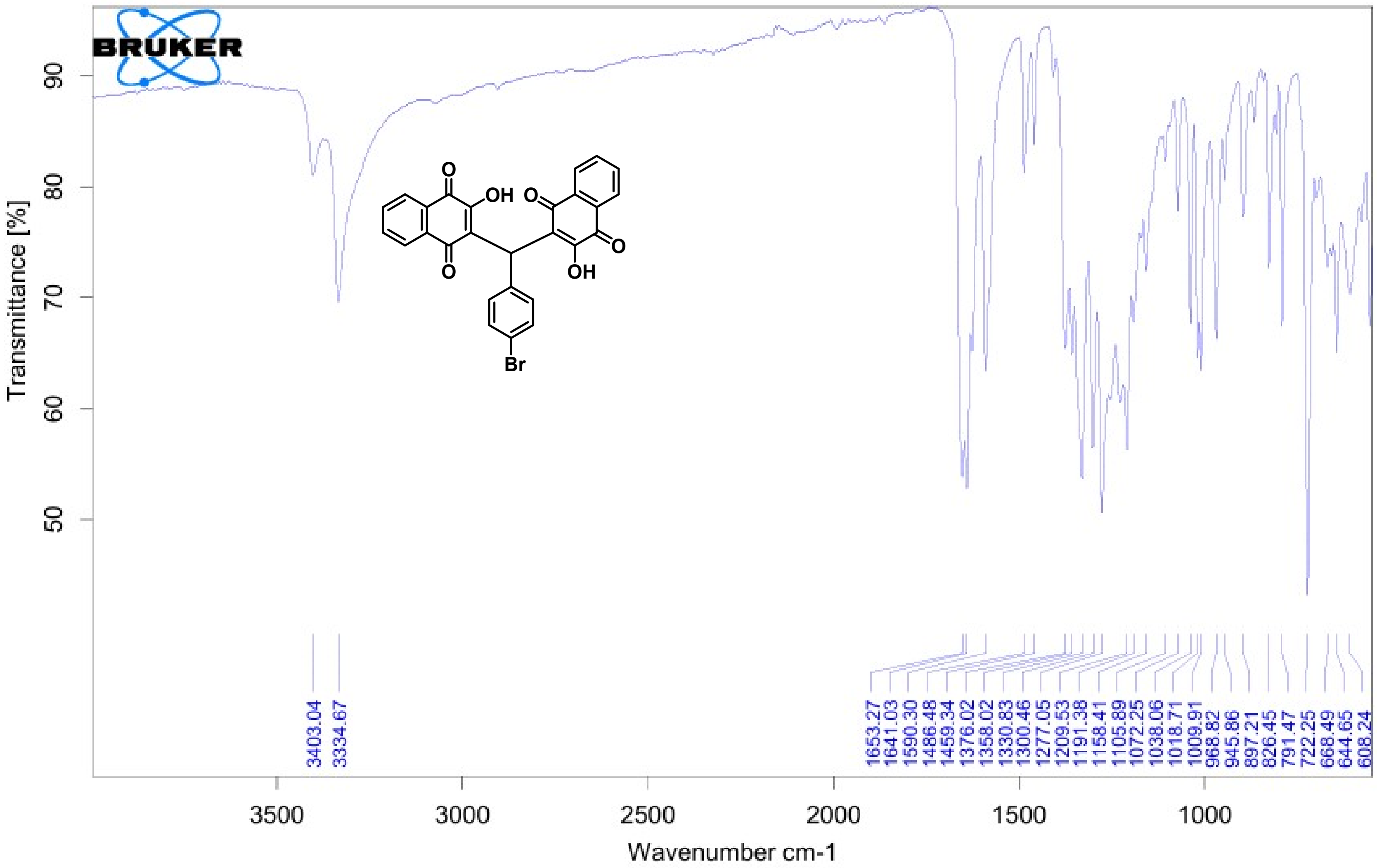

Figure S42. FT-IR spectrum of **3n**.

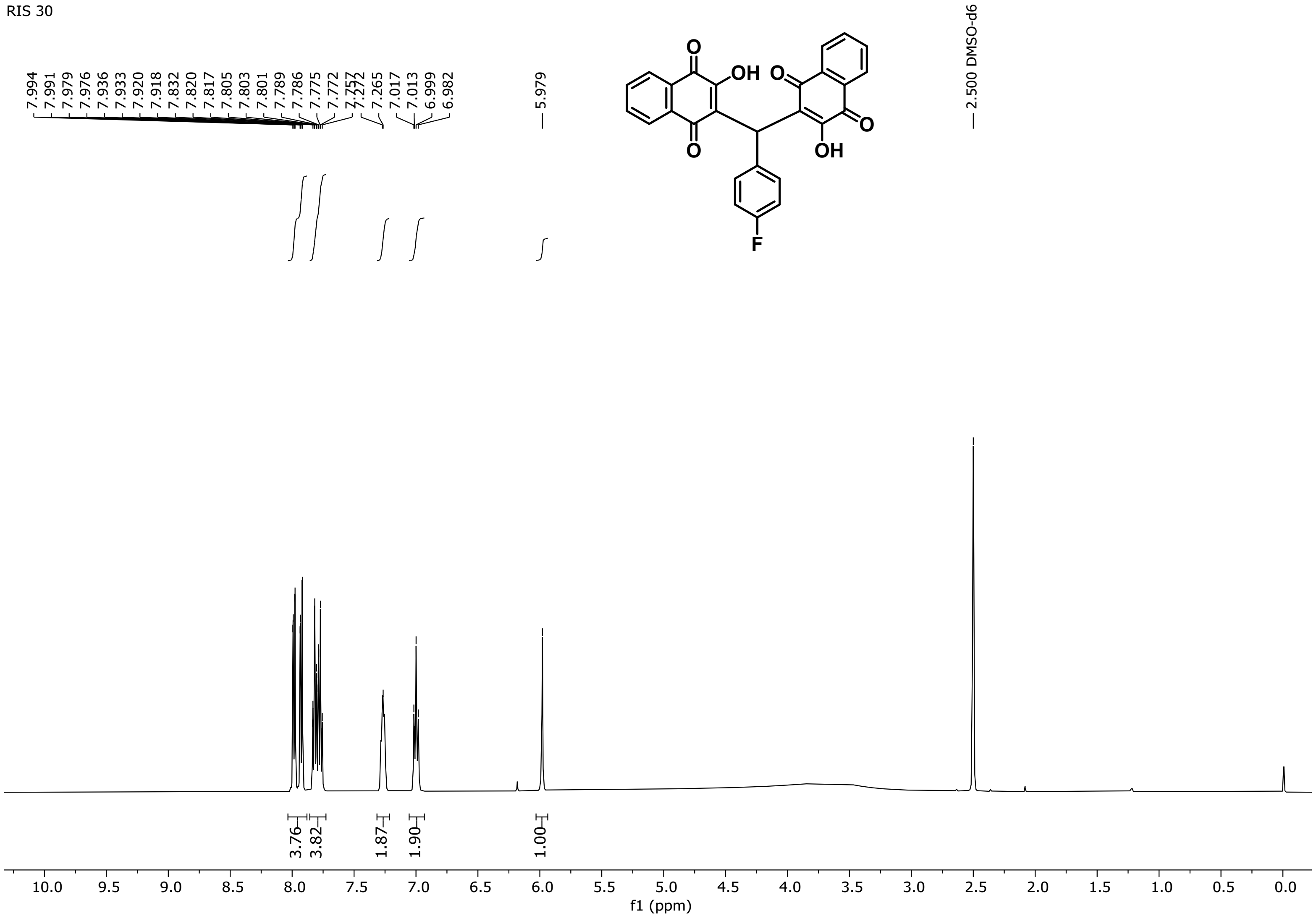

Figure S43. ^{1}H NMR spectrum of **3o** (500 MHz, DMSO-d$_6$).

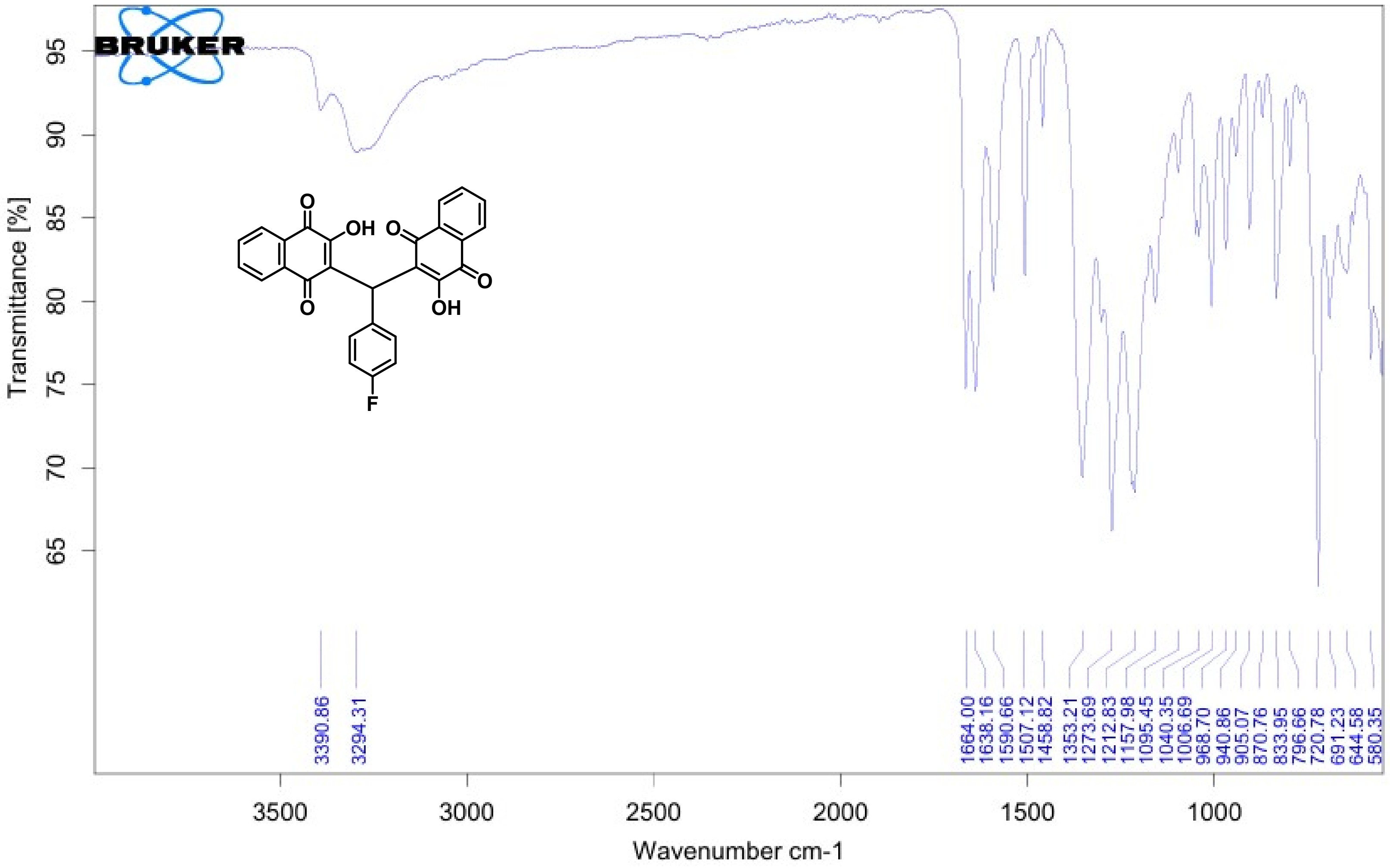

Figure S44. FT-IR spectrum of **3o**.

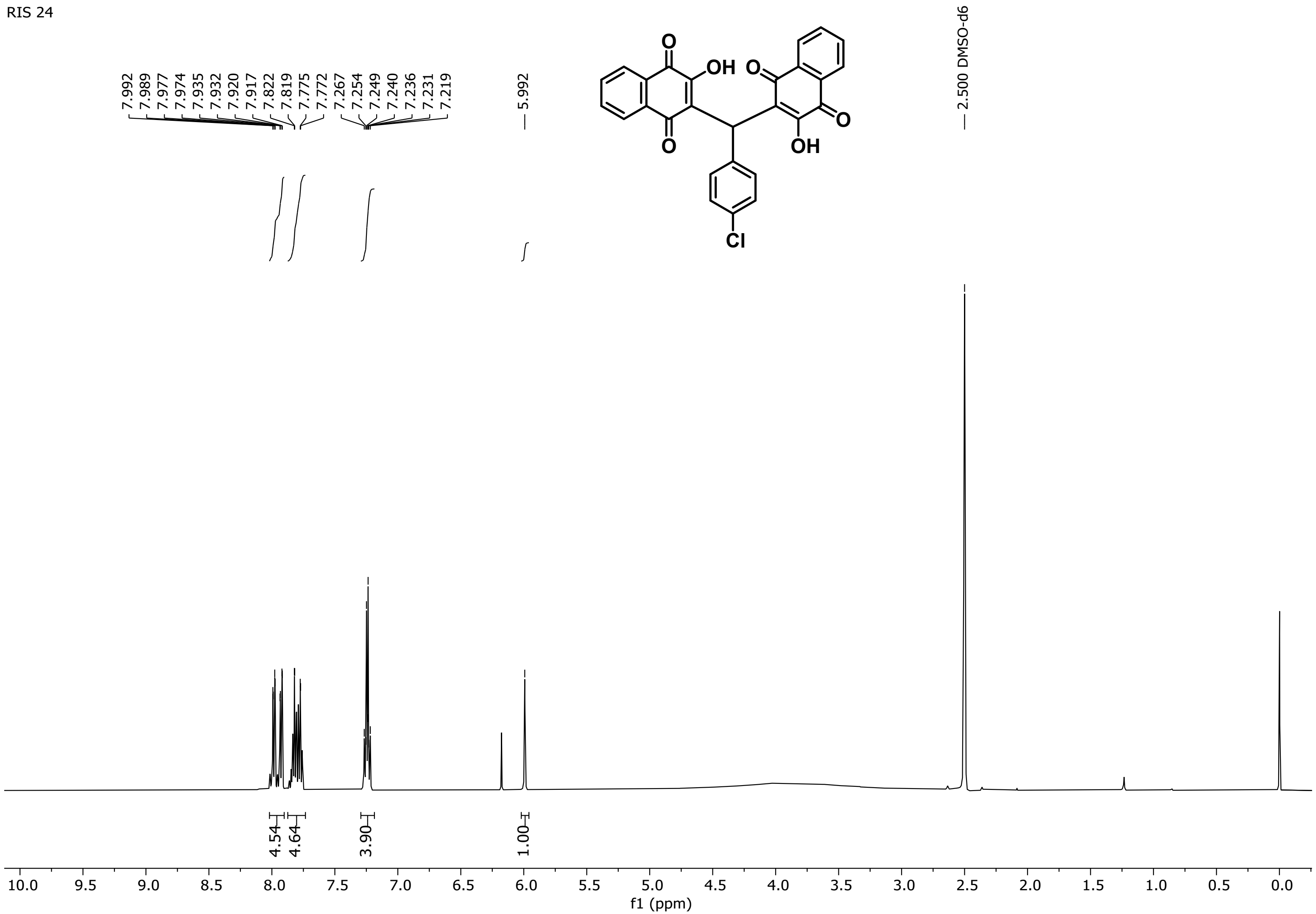

Figure S45. ^{1}H NMR spectrum of **3p** (500 MHz, DMSO-d_6).

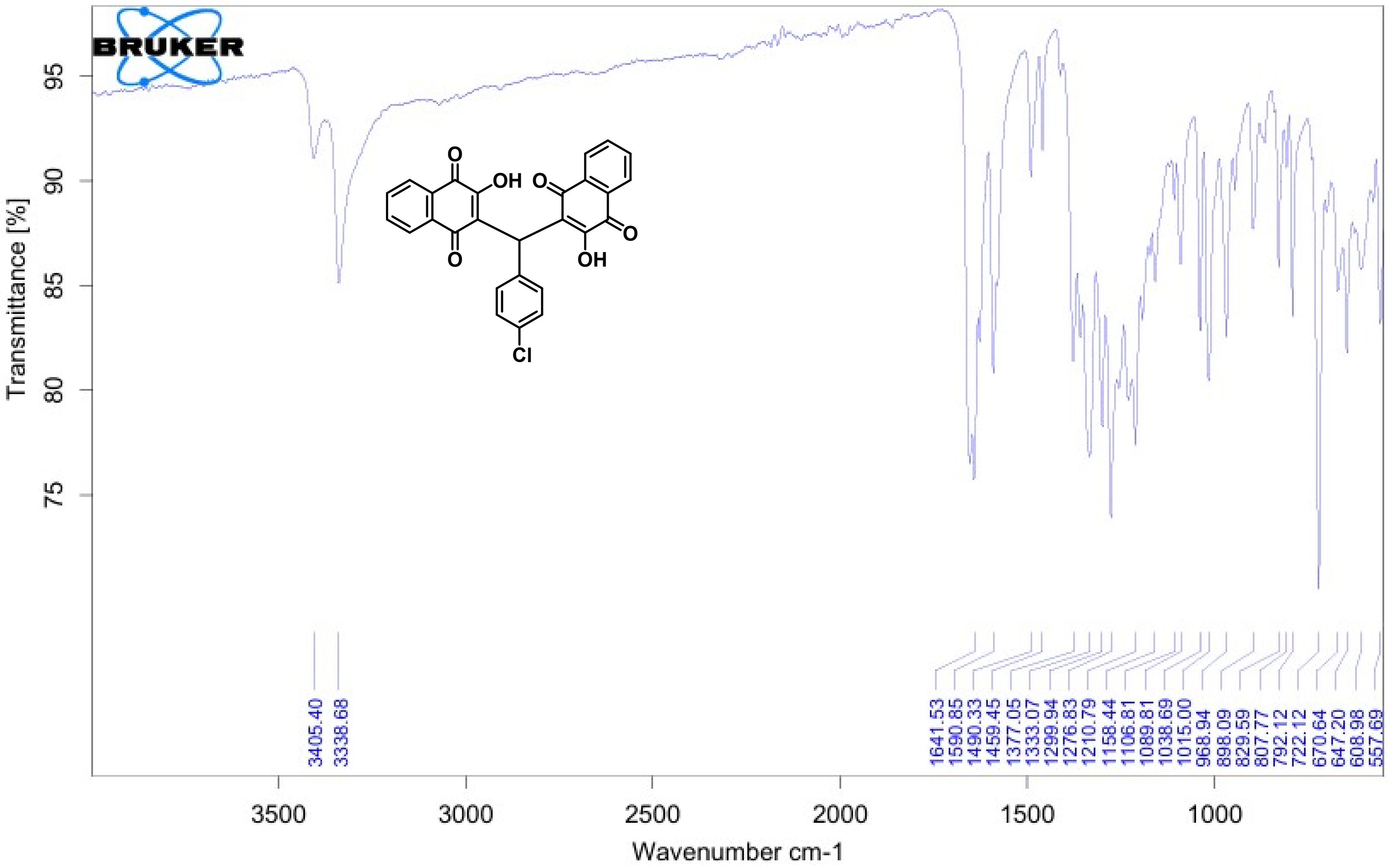

Figure S46. FT-IR spectrum of **3p**.

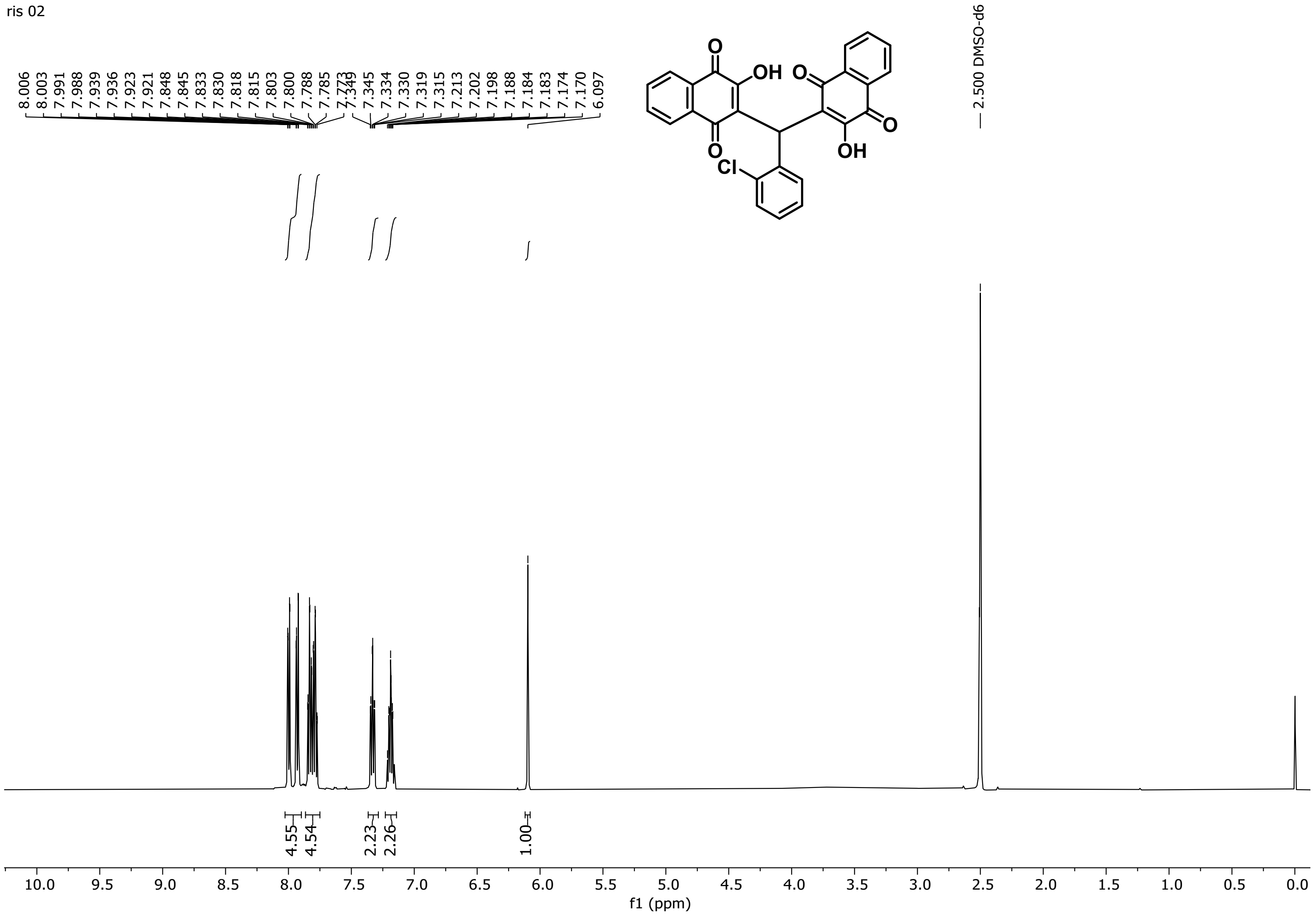

Figure S47. ^{1}H NMR spectrum of **3q** (500 MHz, DMSO-d_6).

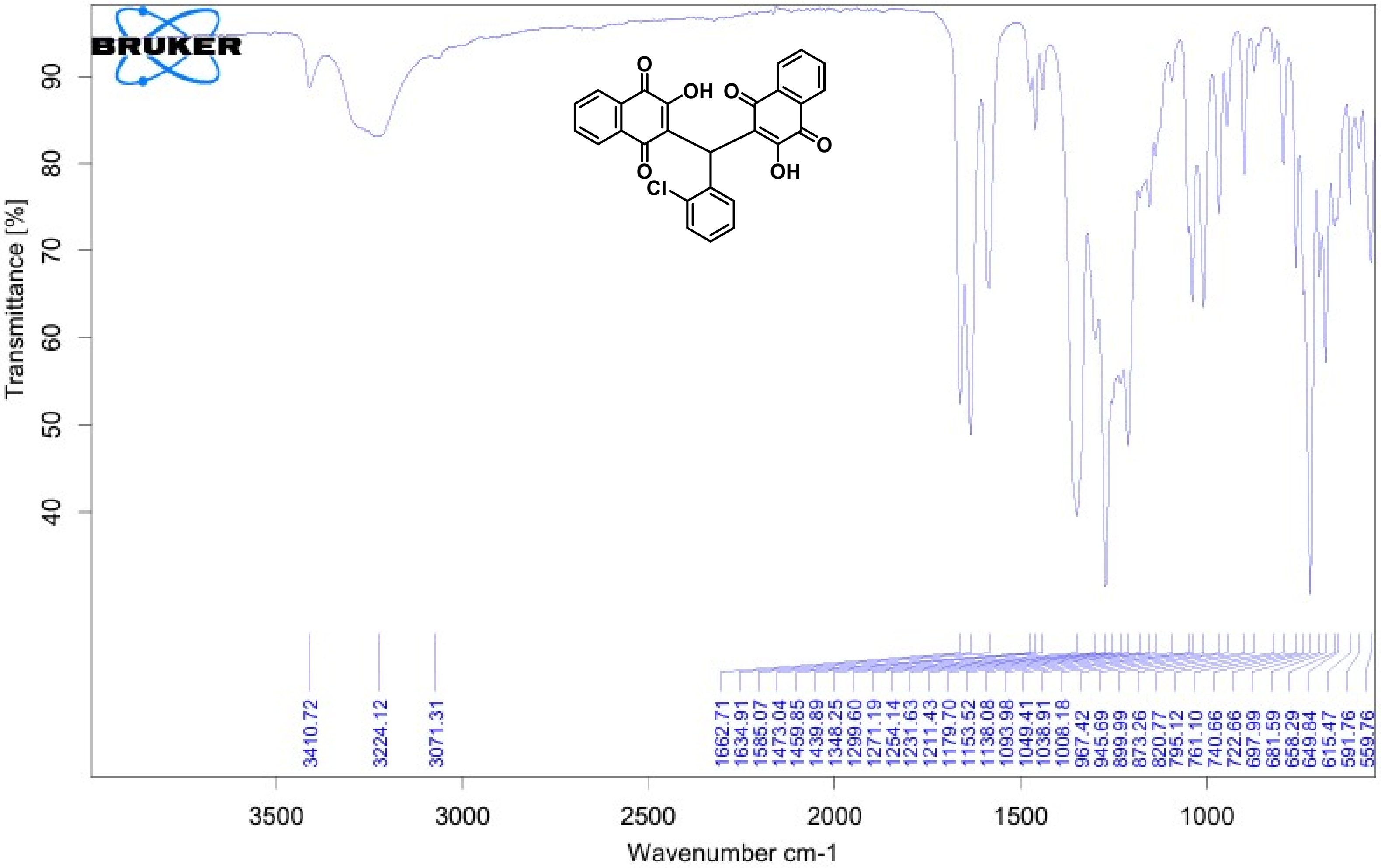

Figure S48. FT-IR spectrum of **3q**.

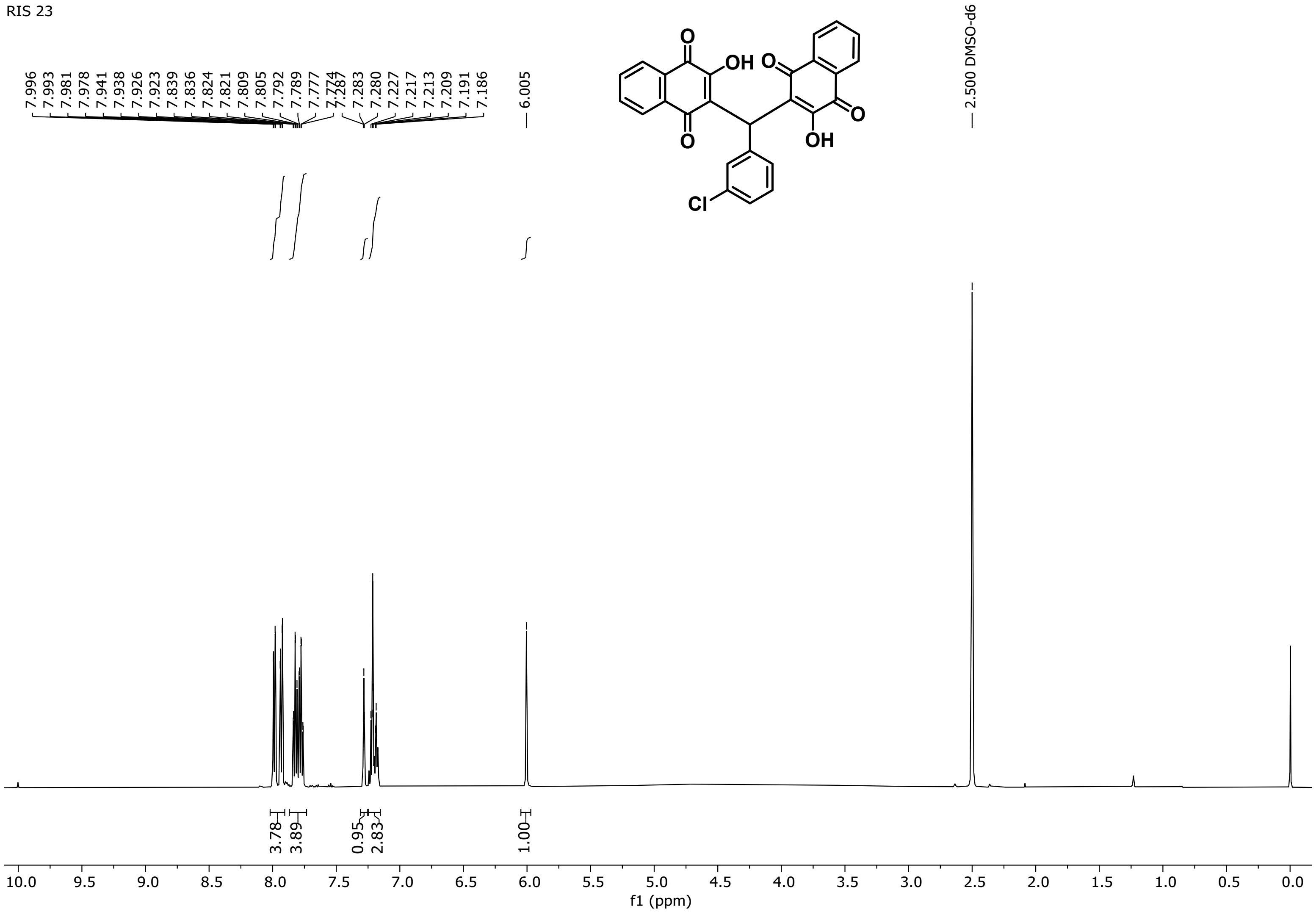

Figure S49. ^{1}H NMR spectrum of **3r** (500 MHz, DMSO-d_6).

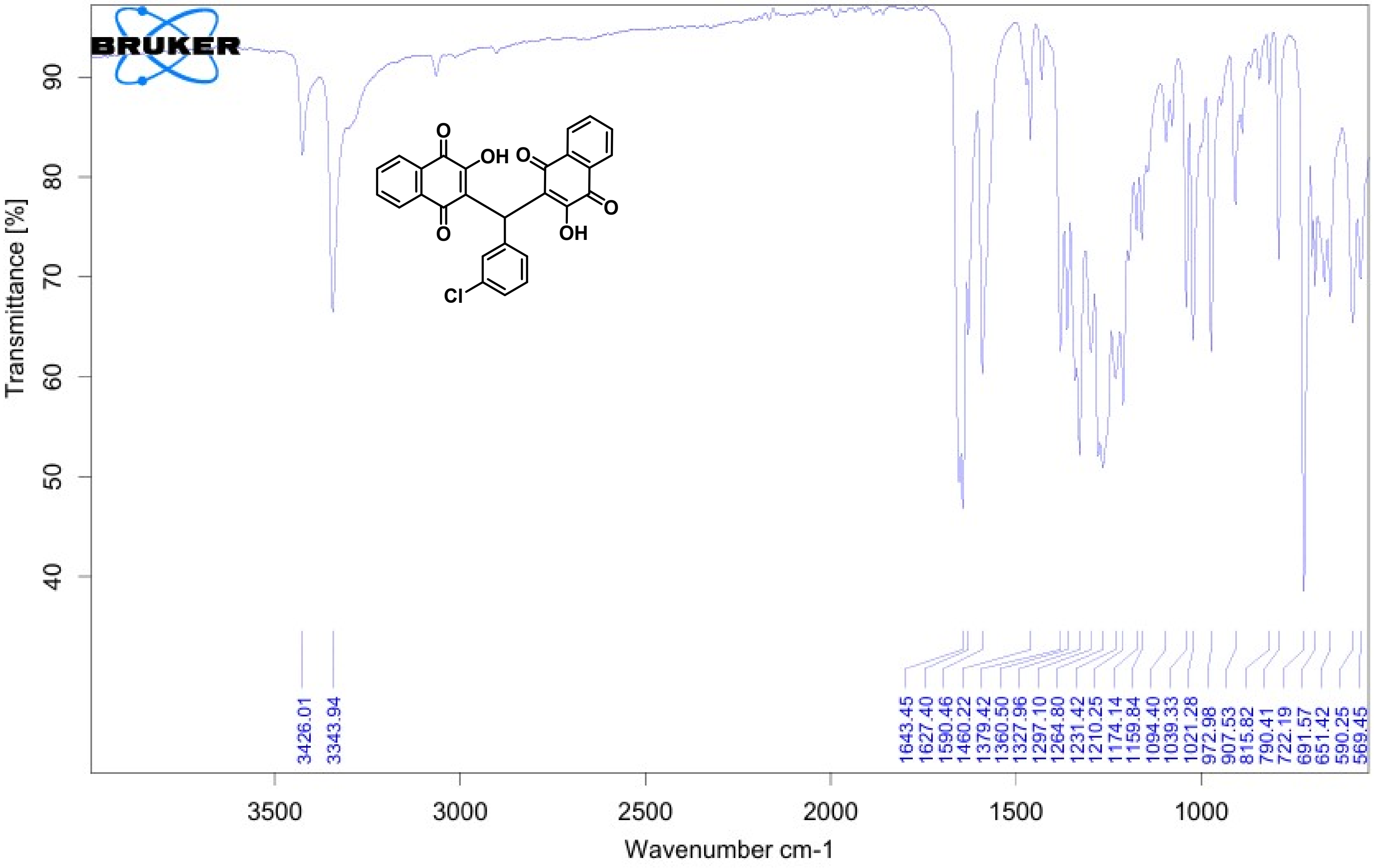

Figure S50. FT-IR spectrum of **3r**.

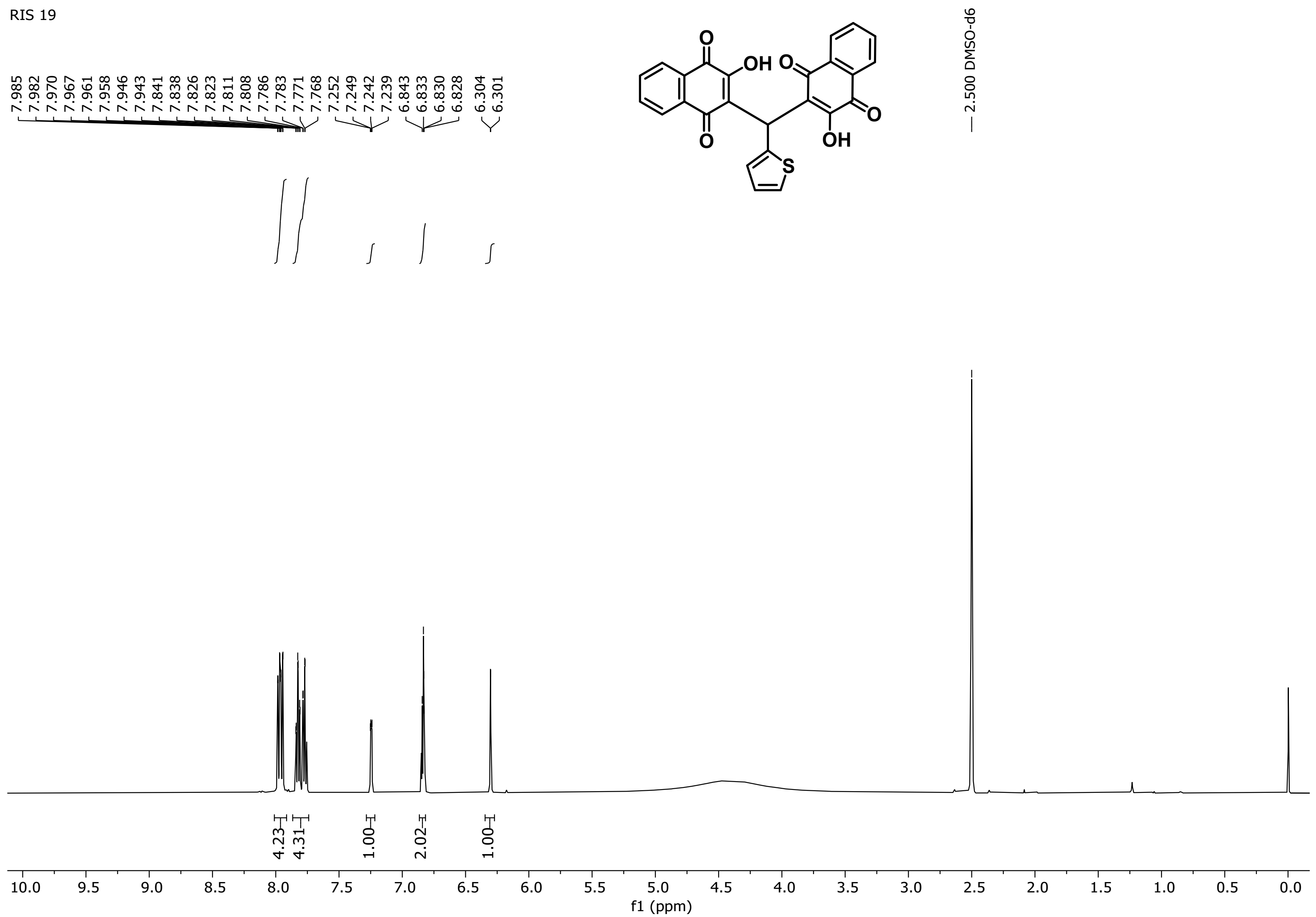

Figure S51. ^{1}H NMR spectrum of **3s** (500 MHz, DMSO-d_6).

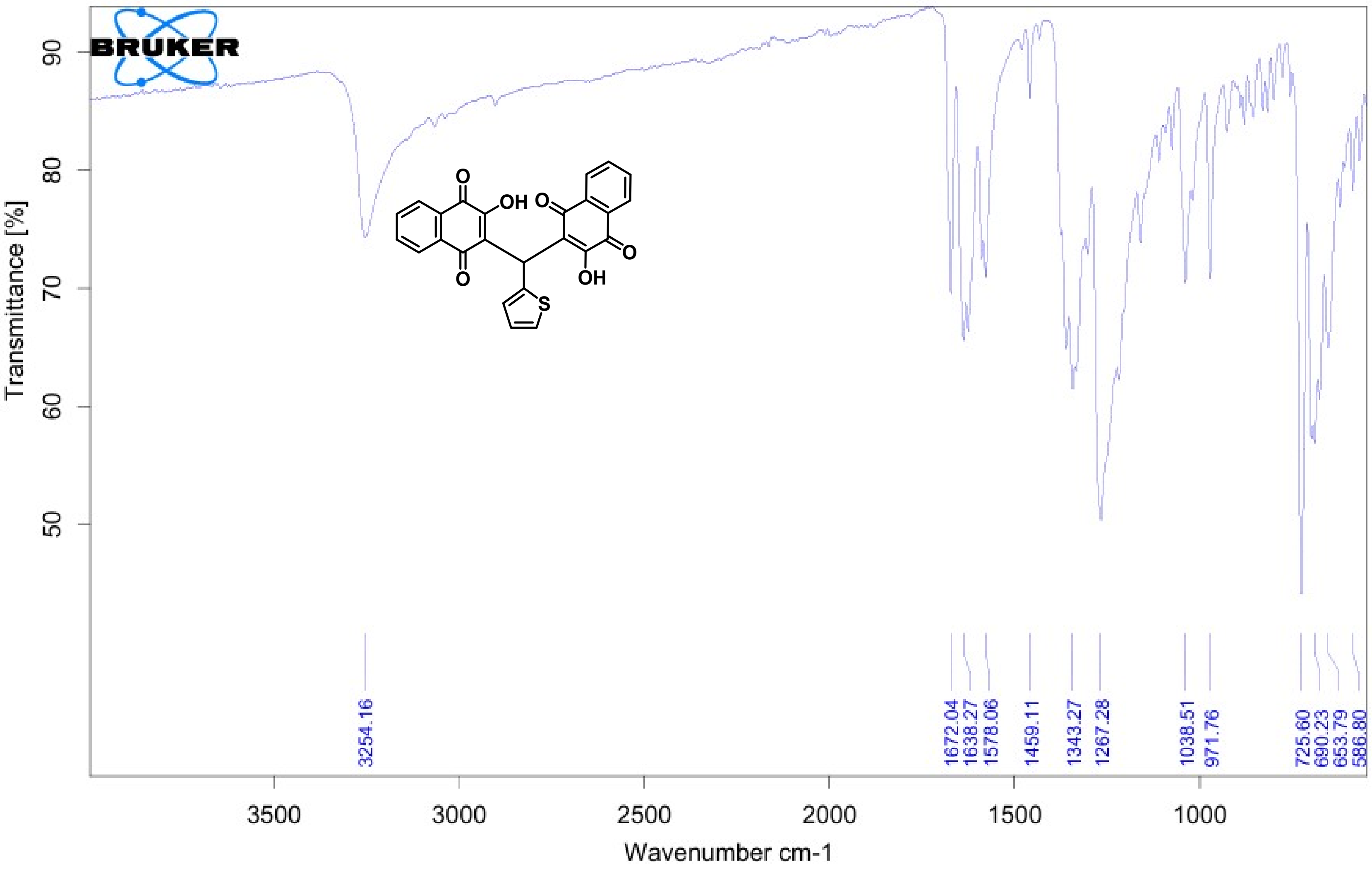

Figure S52. FT-IR spectrum of **3s**.

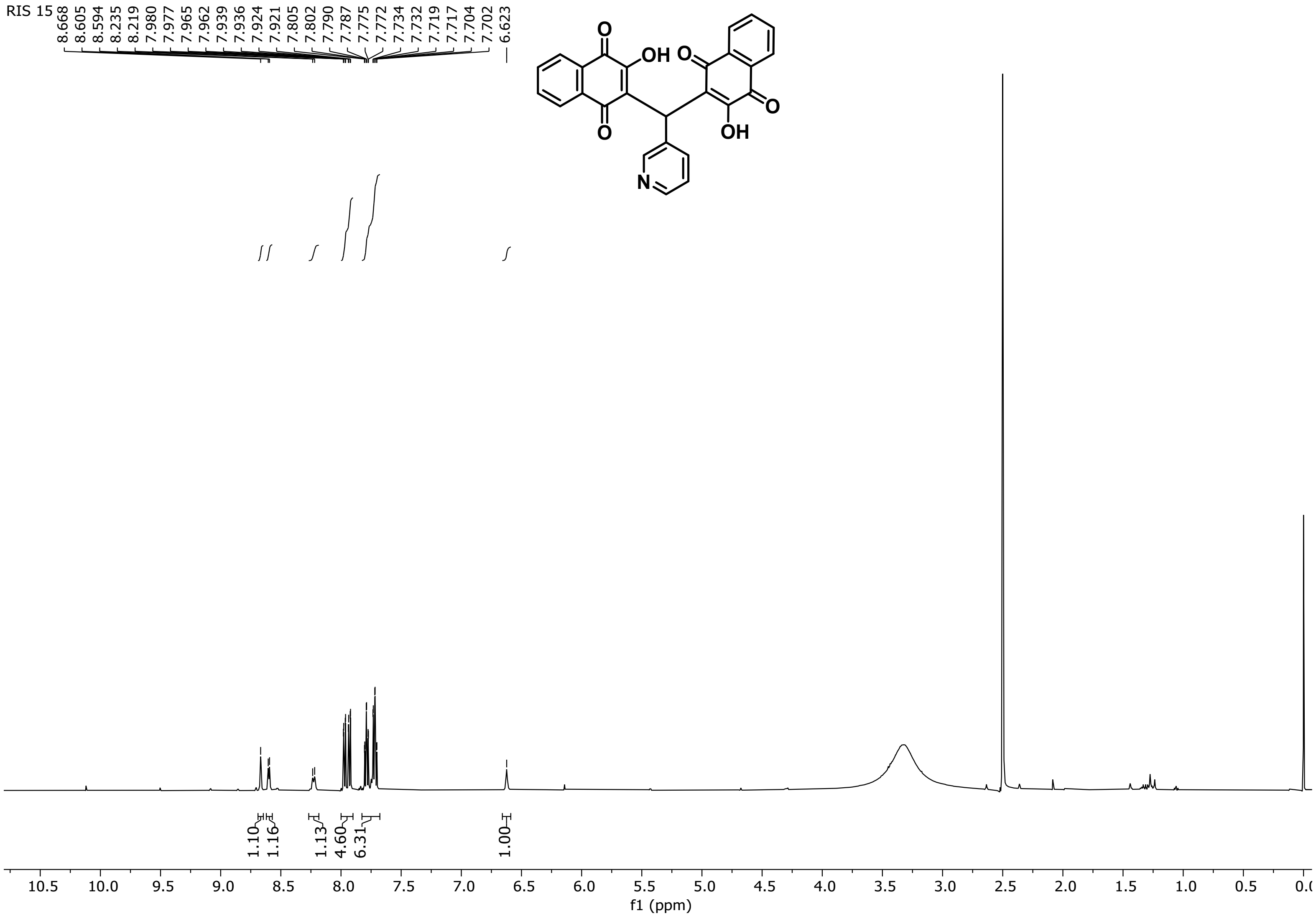

Figure S53. ^{1}H NMR spectrum of **3t** (500 MHz, DMSO-d_6).

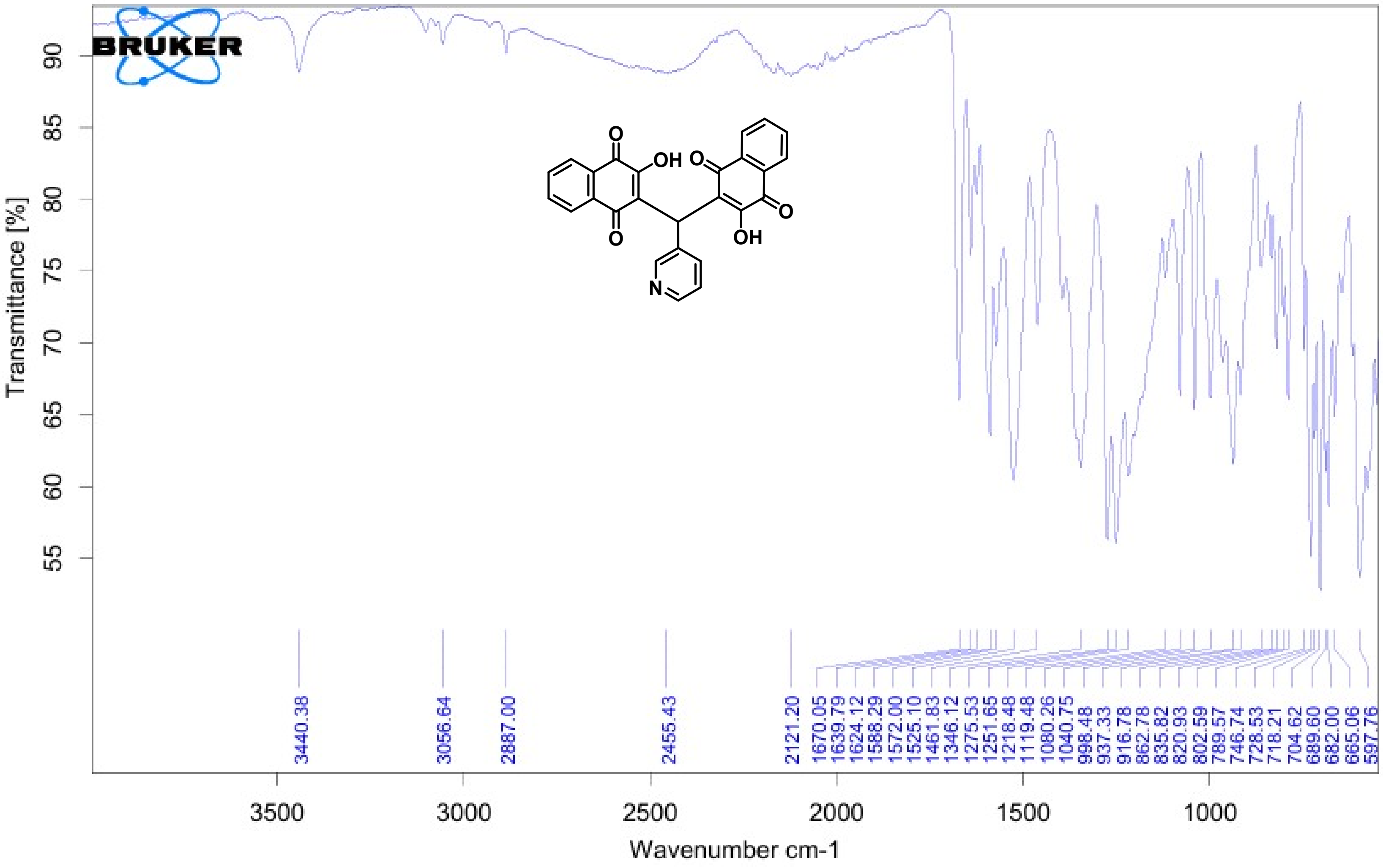

Figure S54. FT-IR spectrum of **3t**.

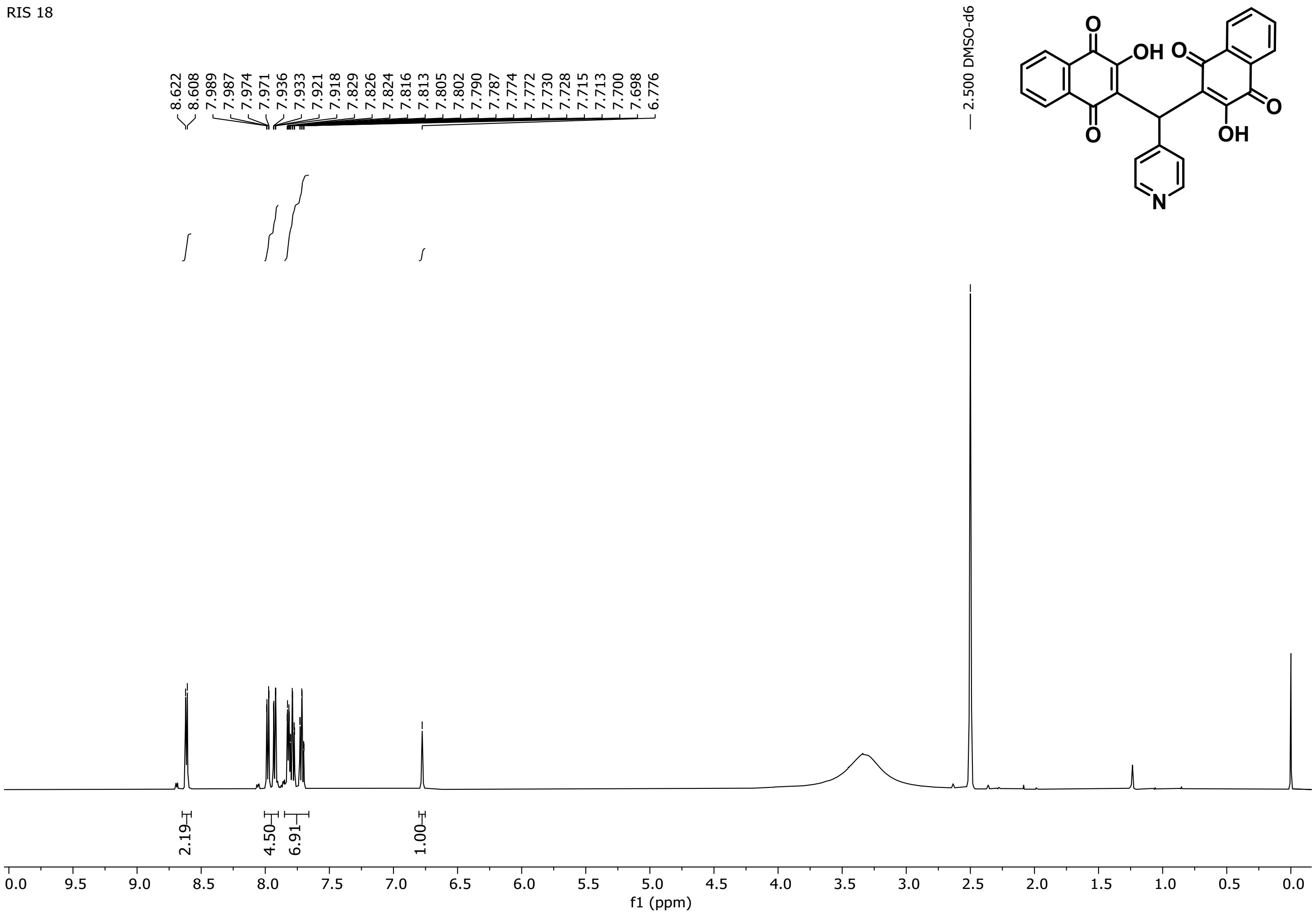

Figure S55. ^{1}H NMR spectrum of **3u** (500 MHz, DMSO-d_6).

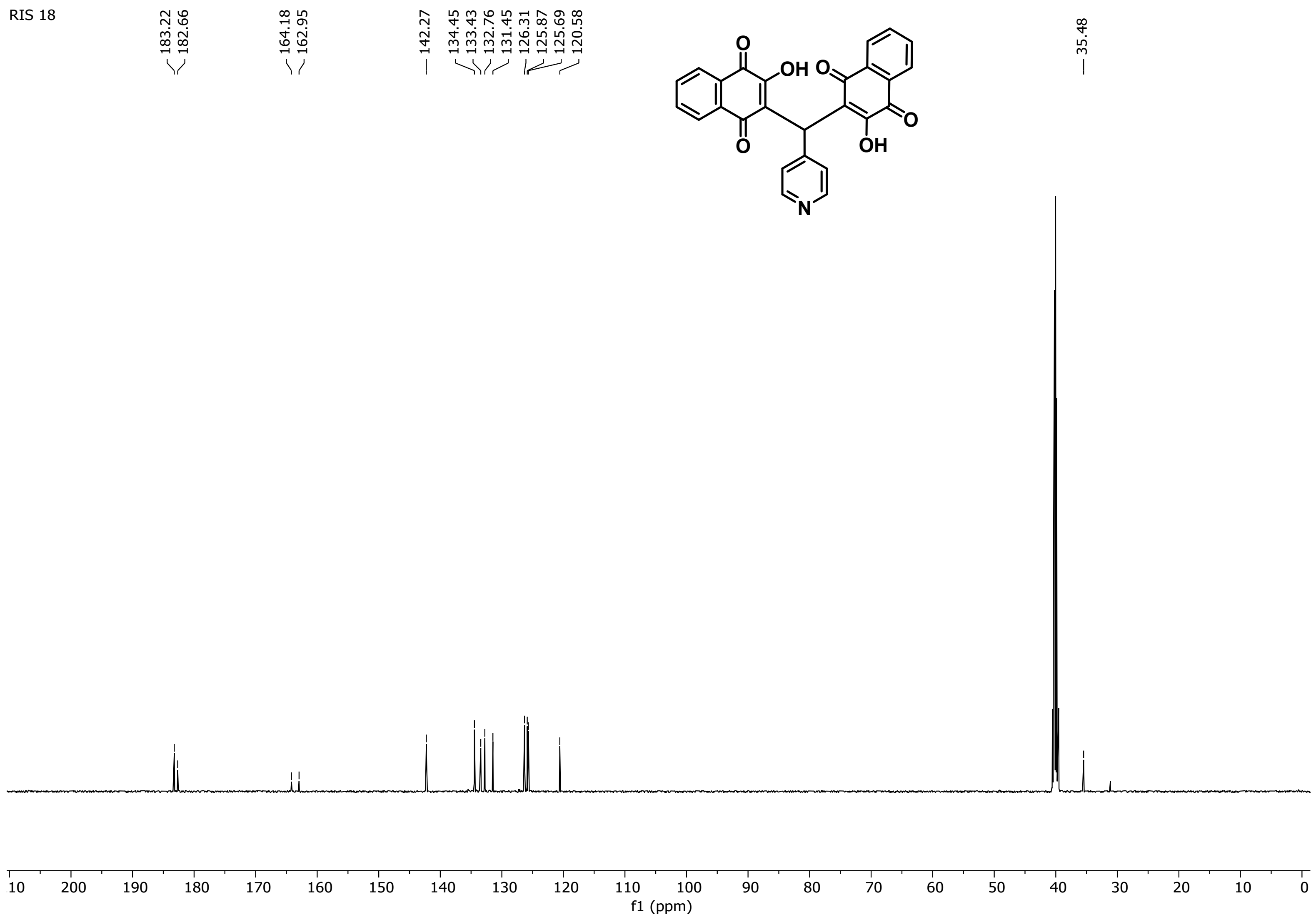

Figure S56. ^{13}C NMR spectrum of **3u** (125 MHz, DMSO-d$_6$).

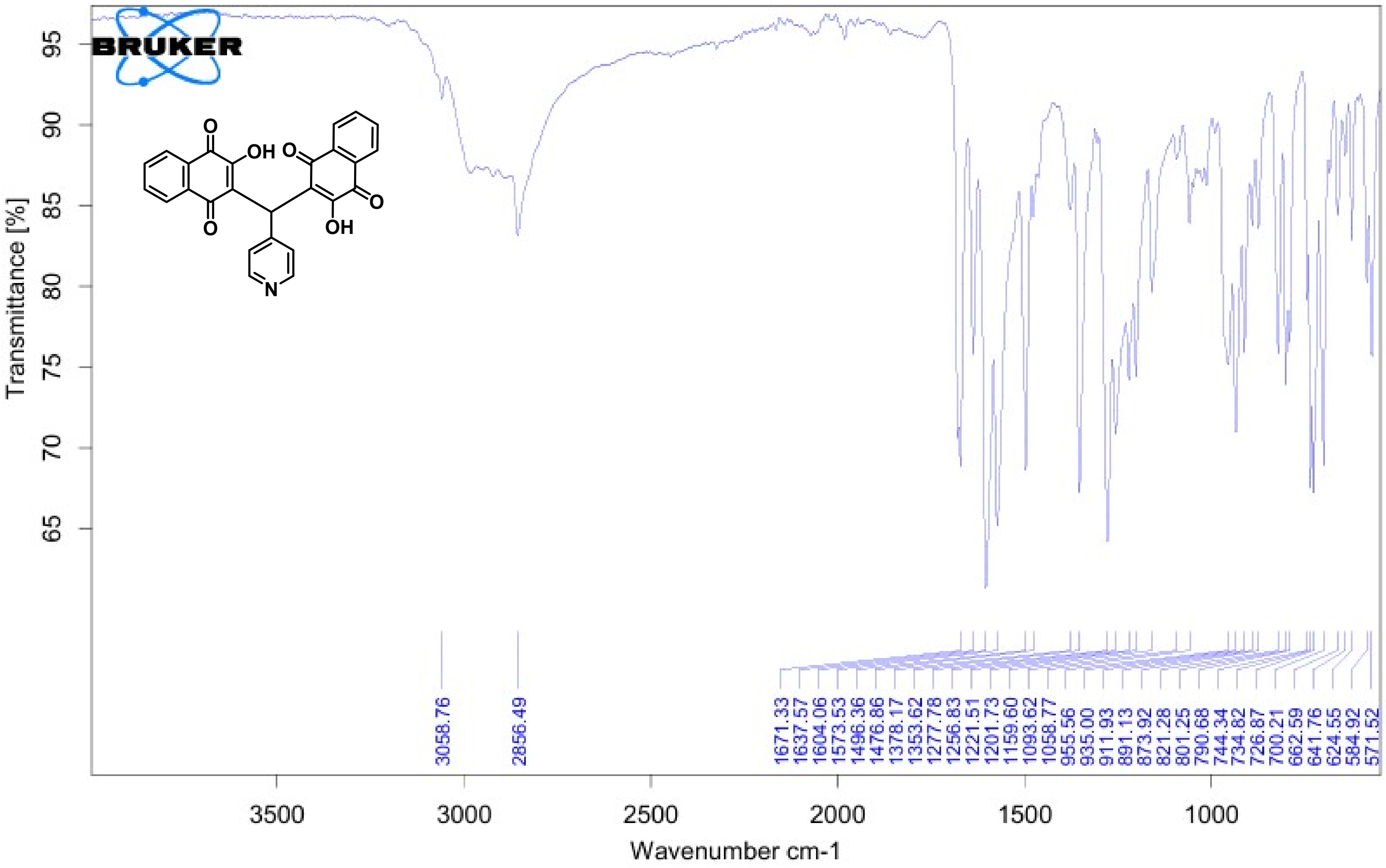

Figure S57. FT-IR spectrum of **3u**.

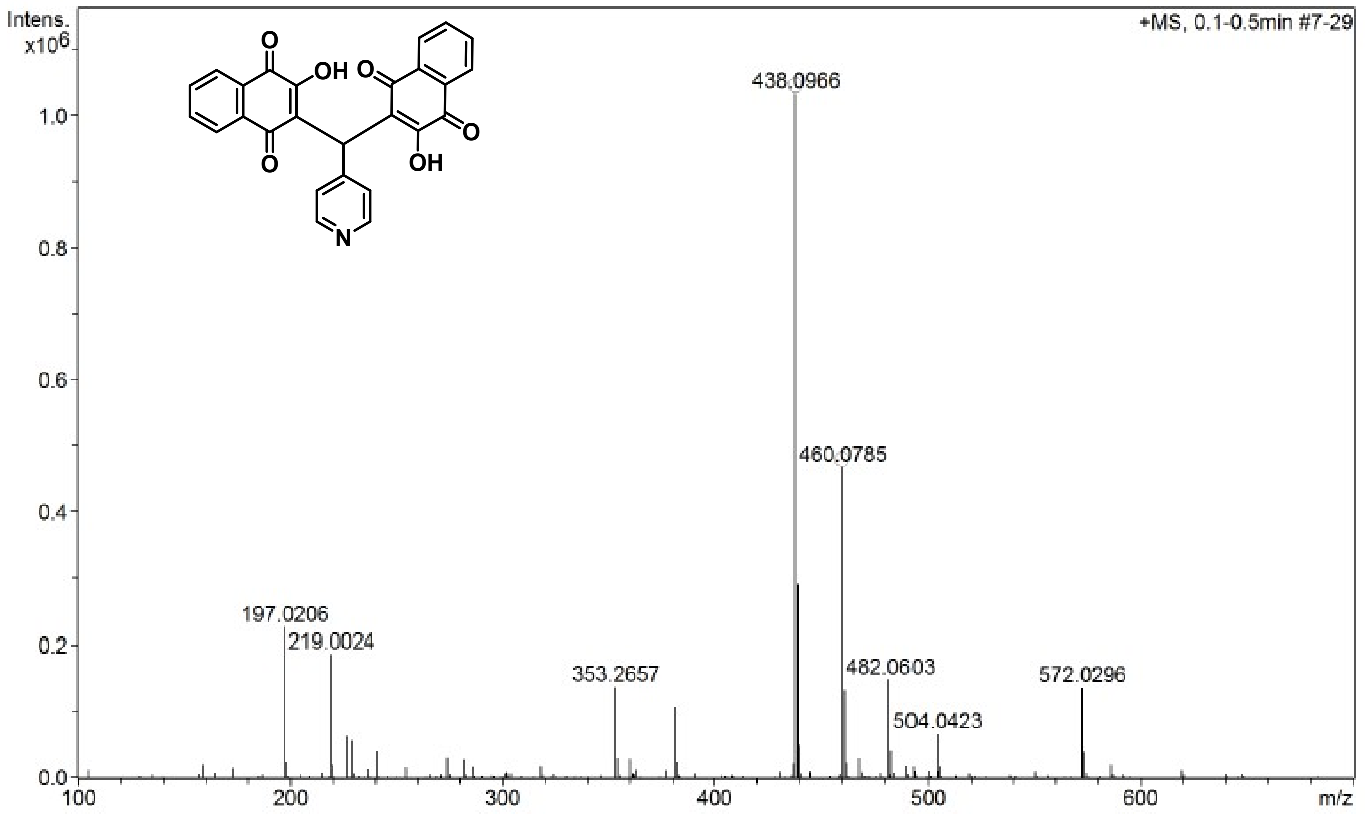

Meas. m/z	#	Ion Formula	m/z	err [ppm]	Mean err [ppm]	rdb	N-Rule	e⁻ Conf	mSigma	Std I	Std Mean m/z	Std I VarNorm	Std m/z Diff	Std Comb Dev
438.096563	1	$C_{26}H_{16}NO_6$	438.097214	1.5	1.7	19.5	ok	even	3.9	7.3	n.a.	n.a.	n.a.	n.a.
	2	$C_{23}H_8N_{11}$	438.095866	-1.6	-2.5	25.5	ok	even	6.4	8.4	n.a.	n.a.	n.a.	n.a.
460.078517	1	$C_{26}H_{15}NNaO_6$	460.079158	1.4	1.2	19.5	ok	even	3.0	5.3	n.a.	n.a.	n.a.	n.a.
	2	$C_{23}H_7N_{11}Na$	460.077810	-1.5	-2.4	25.5	ok	even	6.8	9.2	n.a.	n.a.	n.a.	n.a.

Figure S58. HRMS spectrum of **3u**.

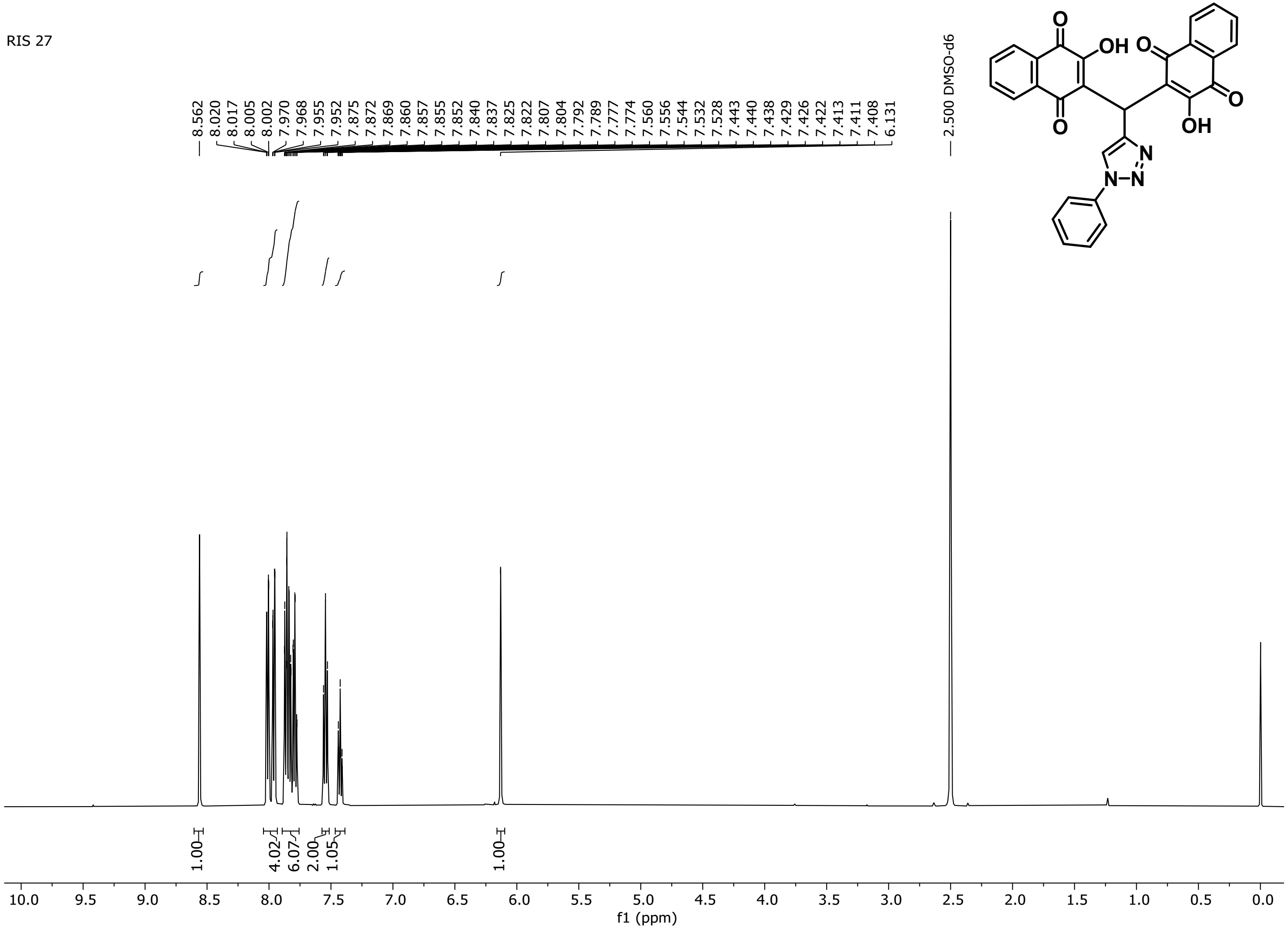

Figure S59. ^{1}H NMR spectrum of **3v** (500 MHz, DMSO-d_6).

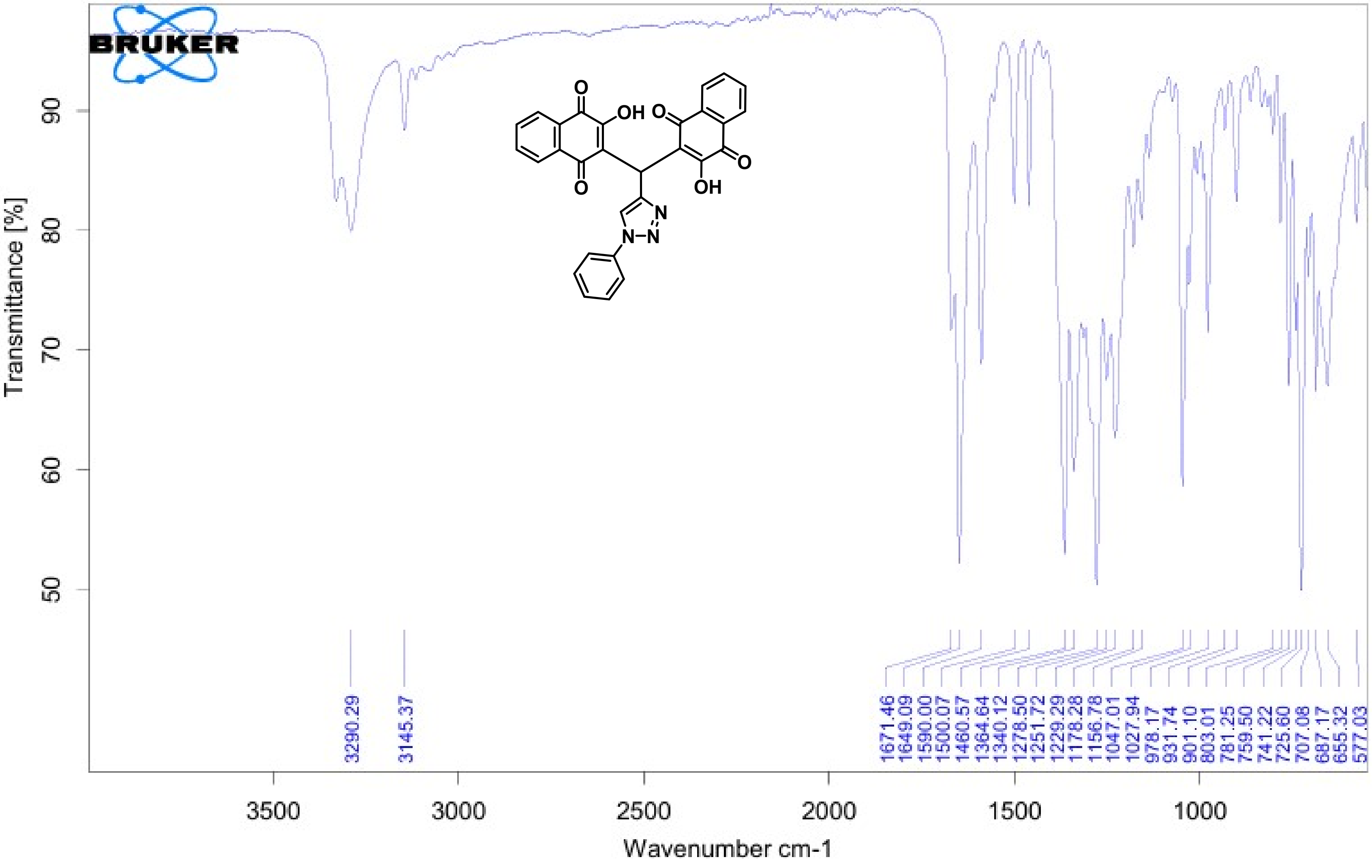

Figure S60. FT-IR spectrum of **3v.**

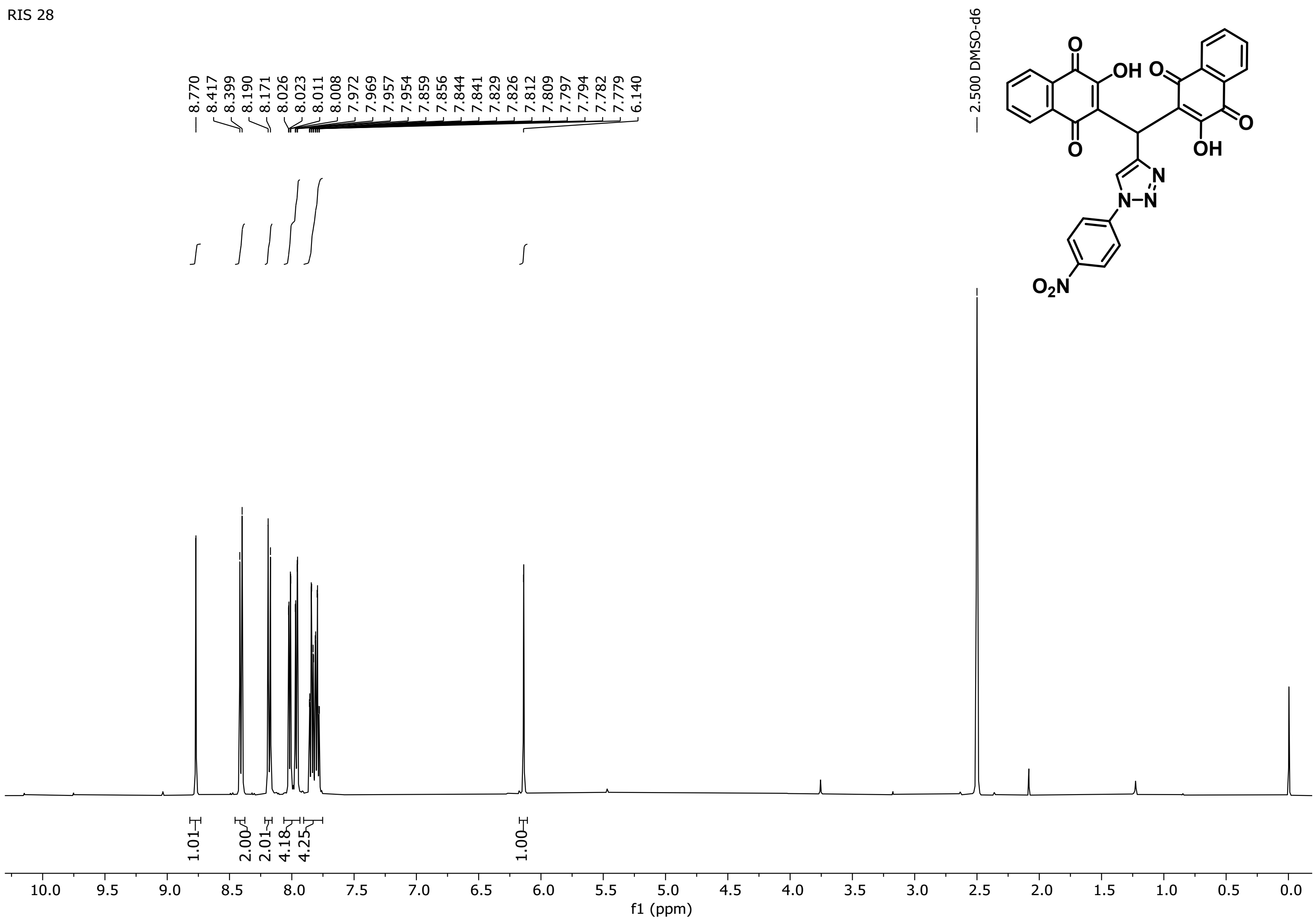

Figure S61. ^{1}H NMR spectrum of **3w** (500 MHz, DMSO-d_6).

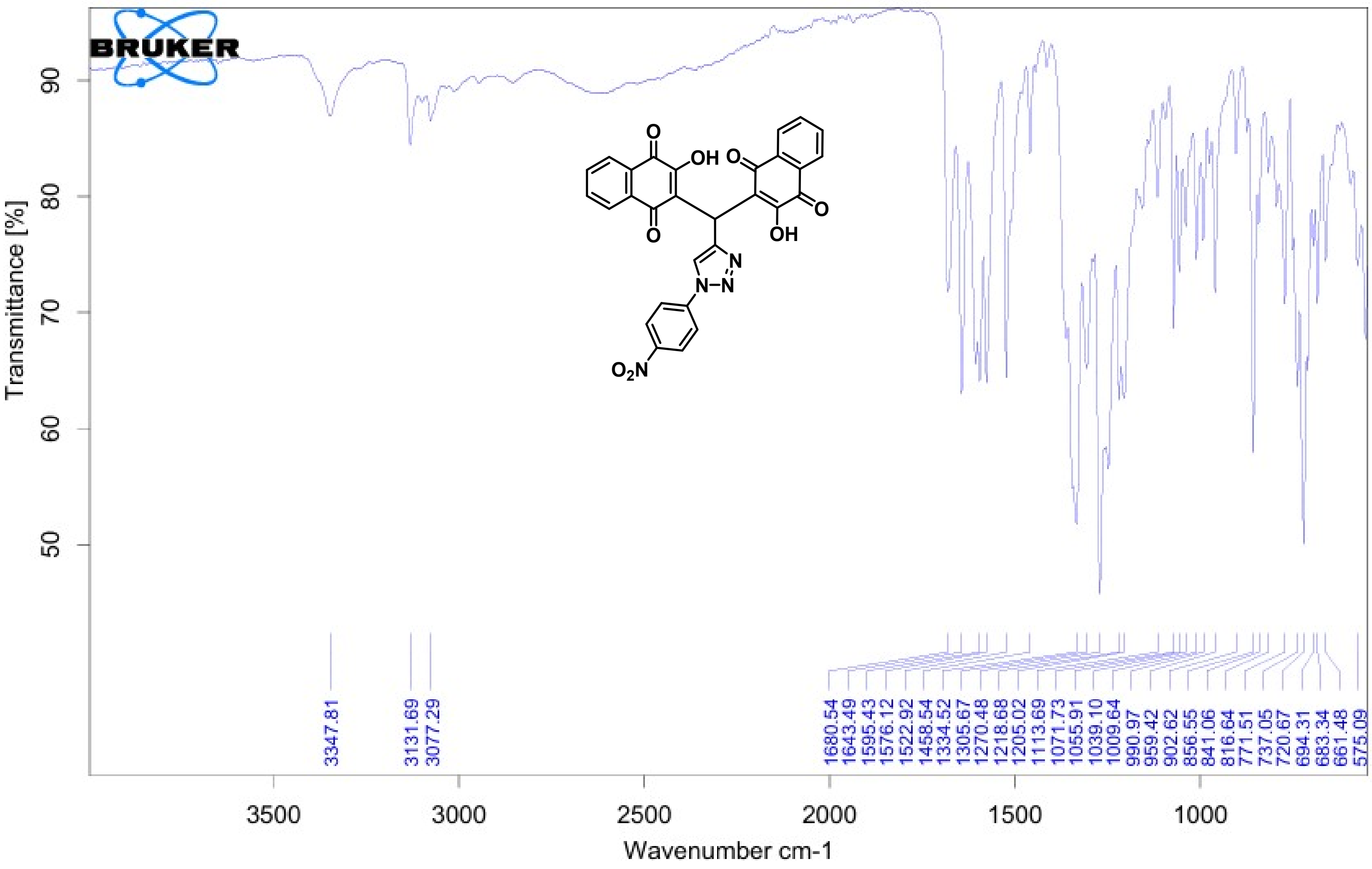

Figure S62. FT-IR spectrum of **3w.**

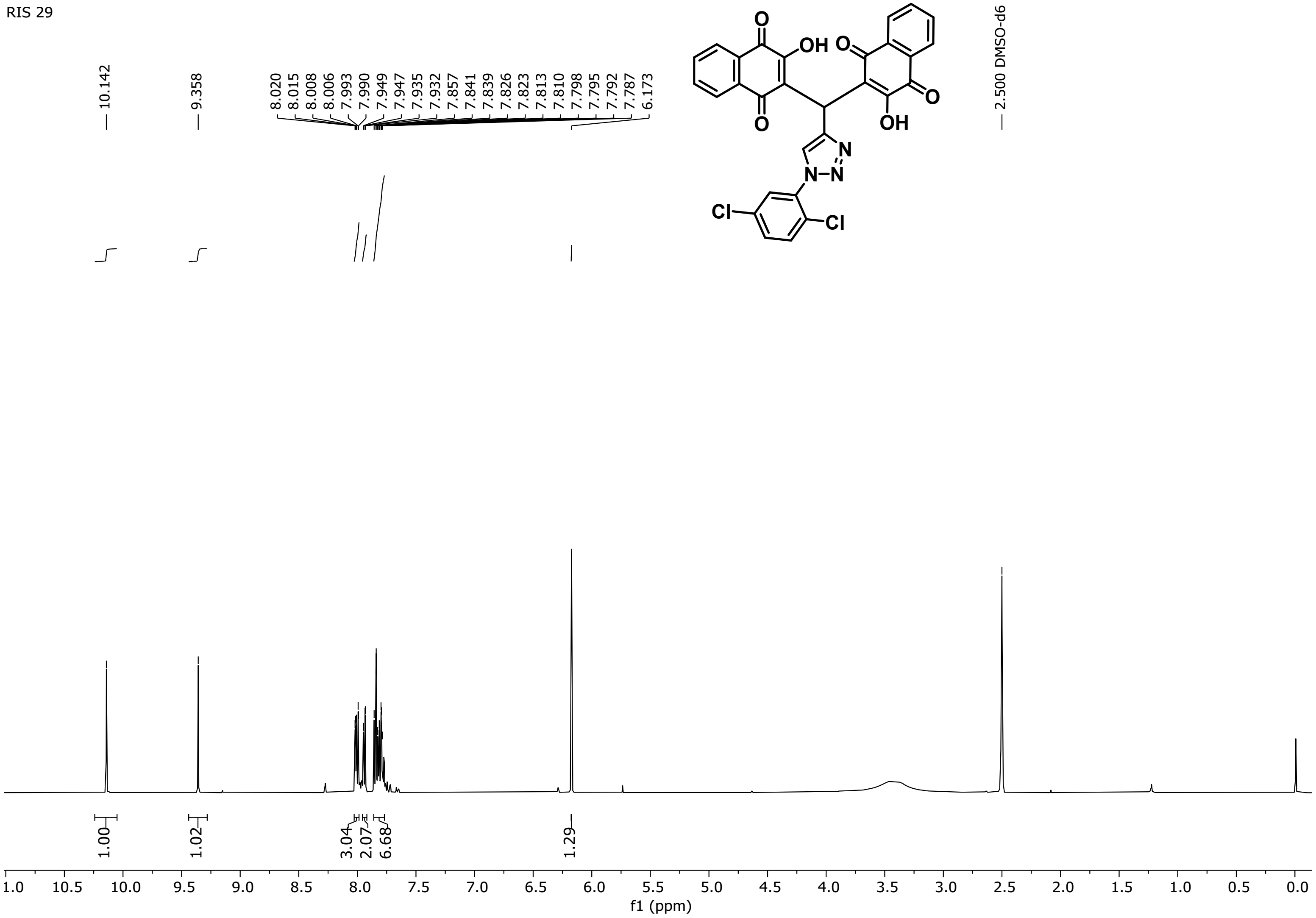

Figure S63. ^{1}H NMR spectrum of **3x** (500 MHz, DMSO-d_6).

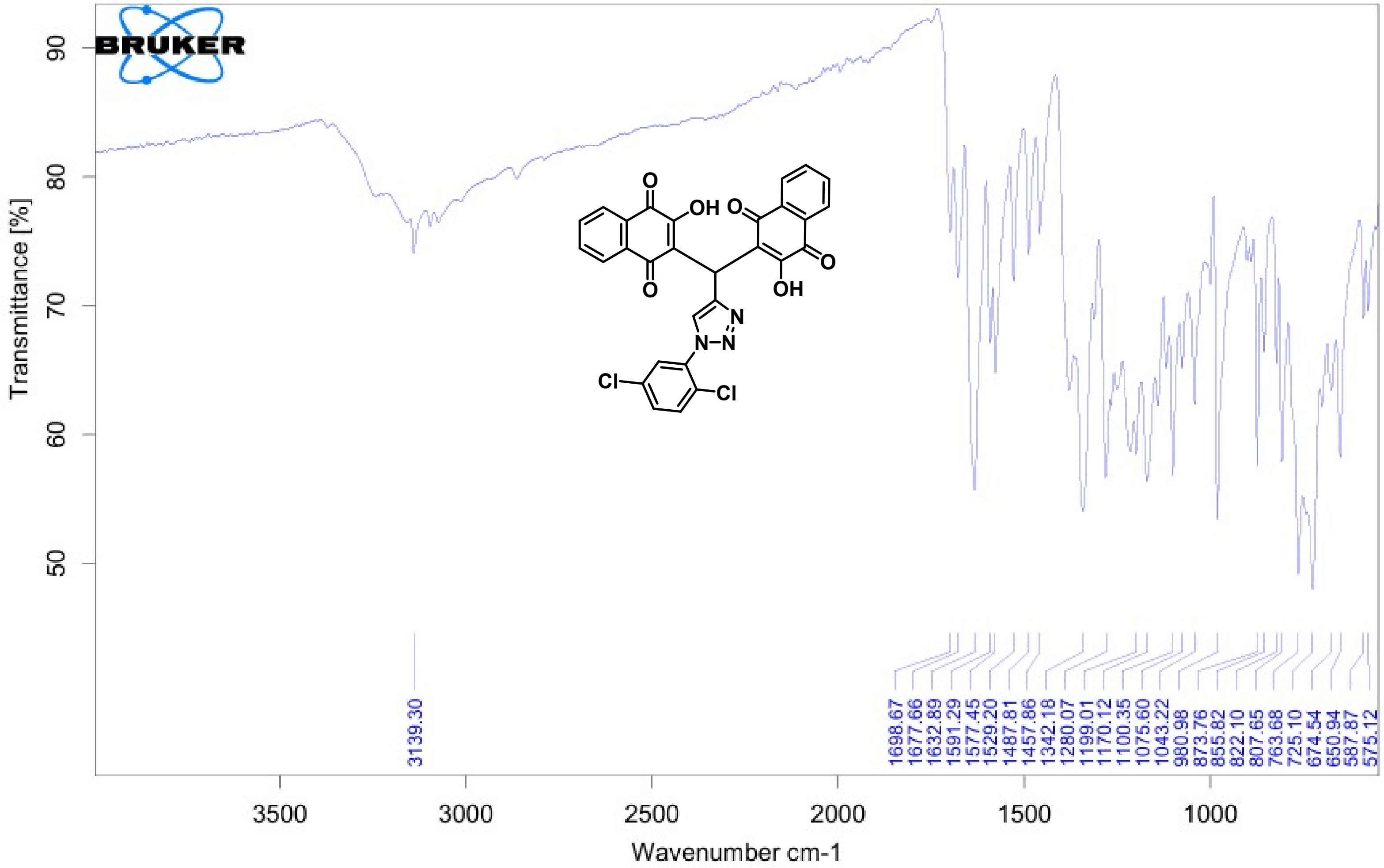

Figure S64. FT-IR spectrum of **3x.**

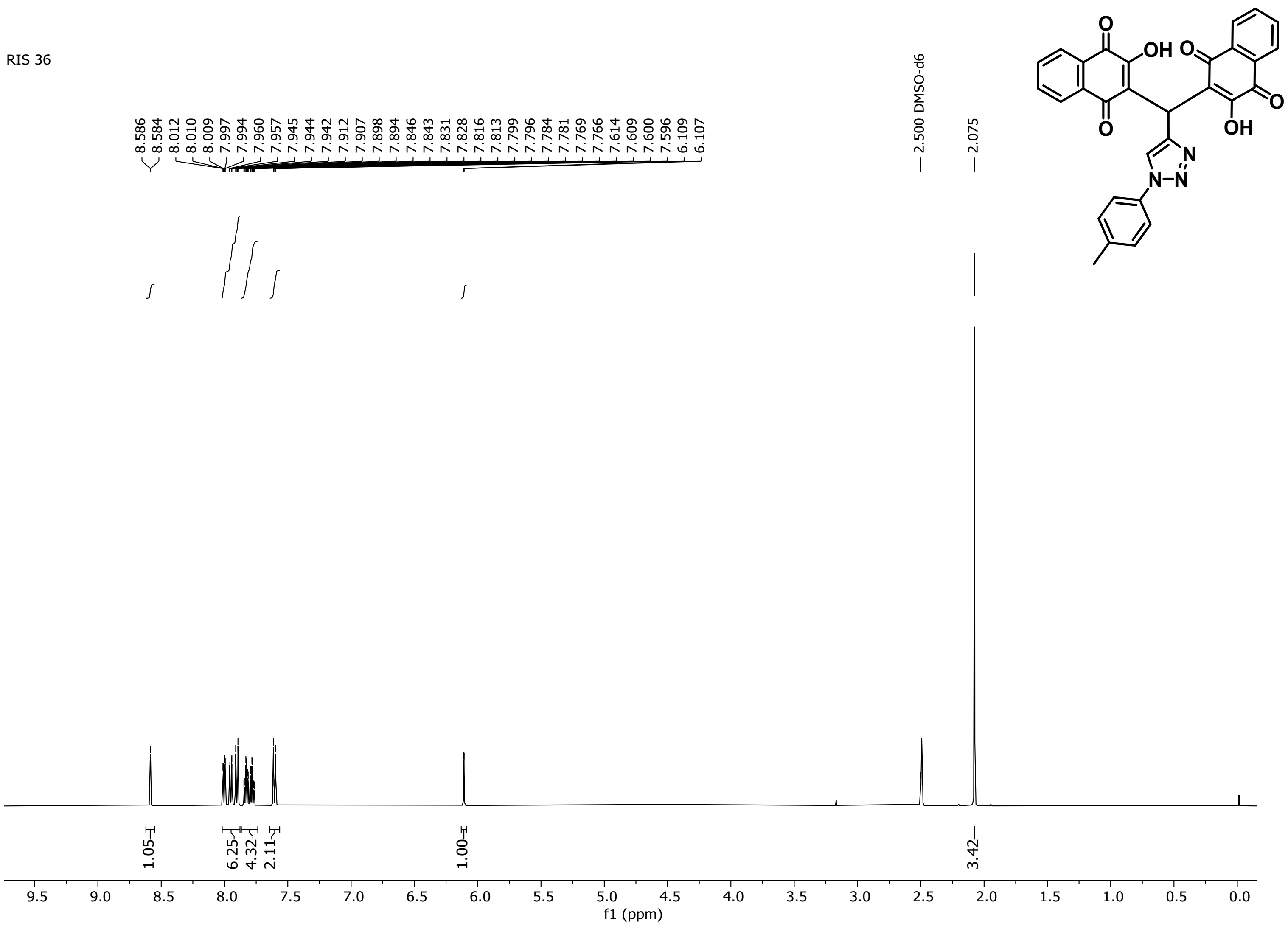

Figure S65. ^{1}H NMR spectrum of **3y** (500 MHz, DMSO-d_6).

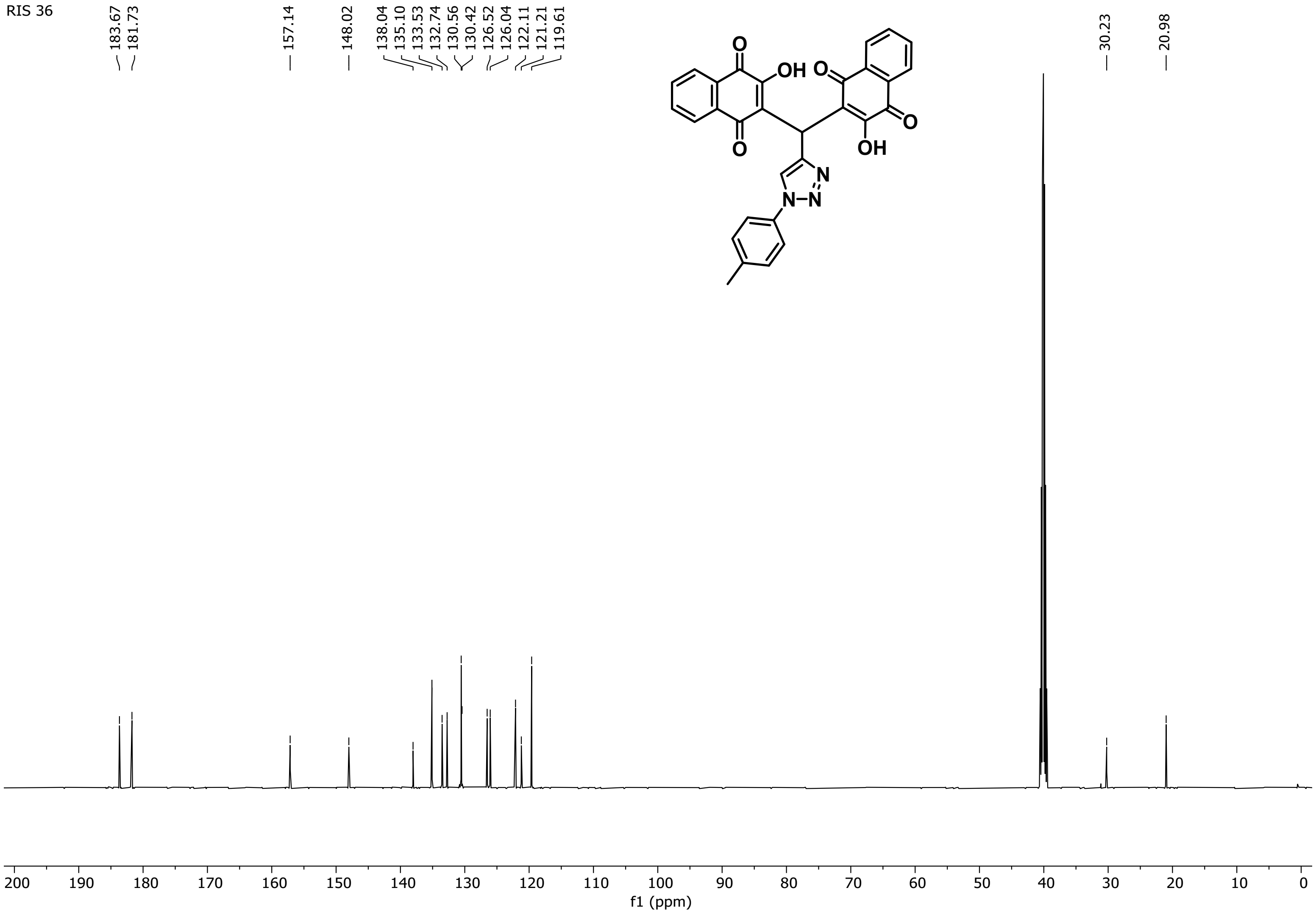

Figure S66. ^{13}C NMR spectrum of **3y** (125 MHz, DMSO-d_6).

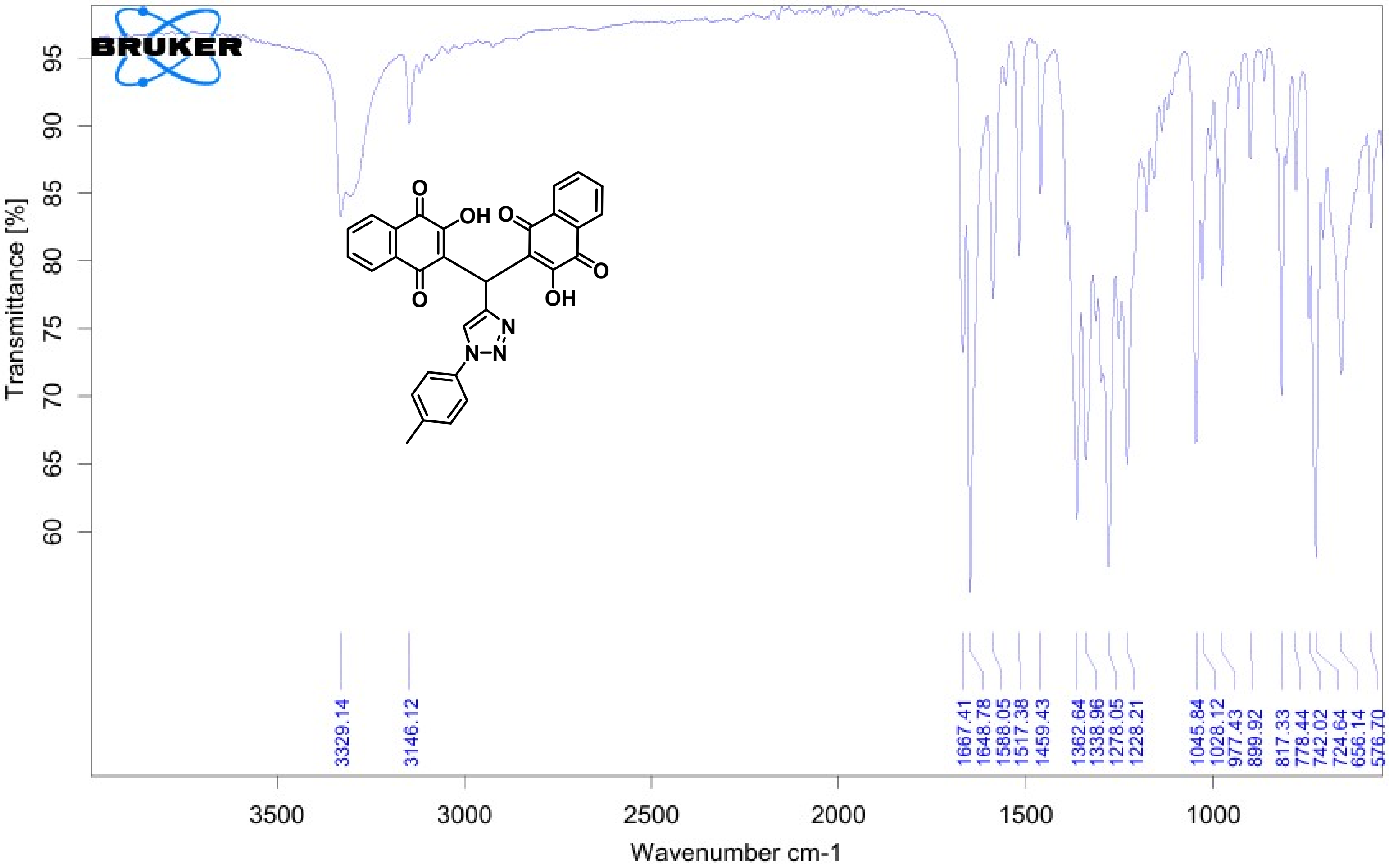

Figure S67. FT-IR spectrum of **3y.**

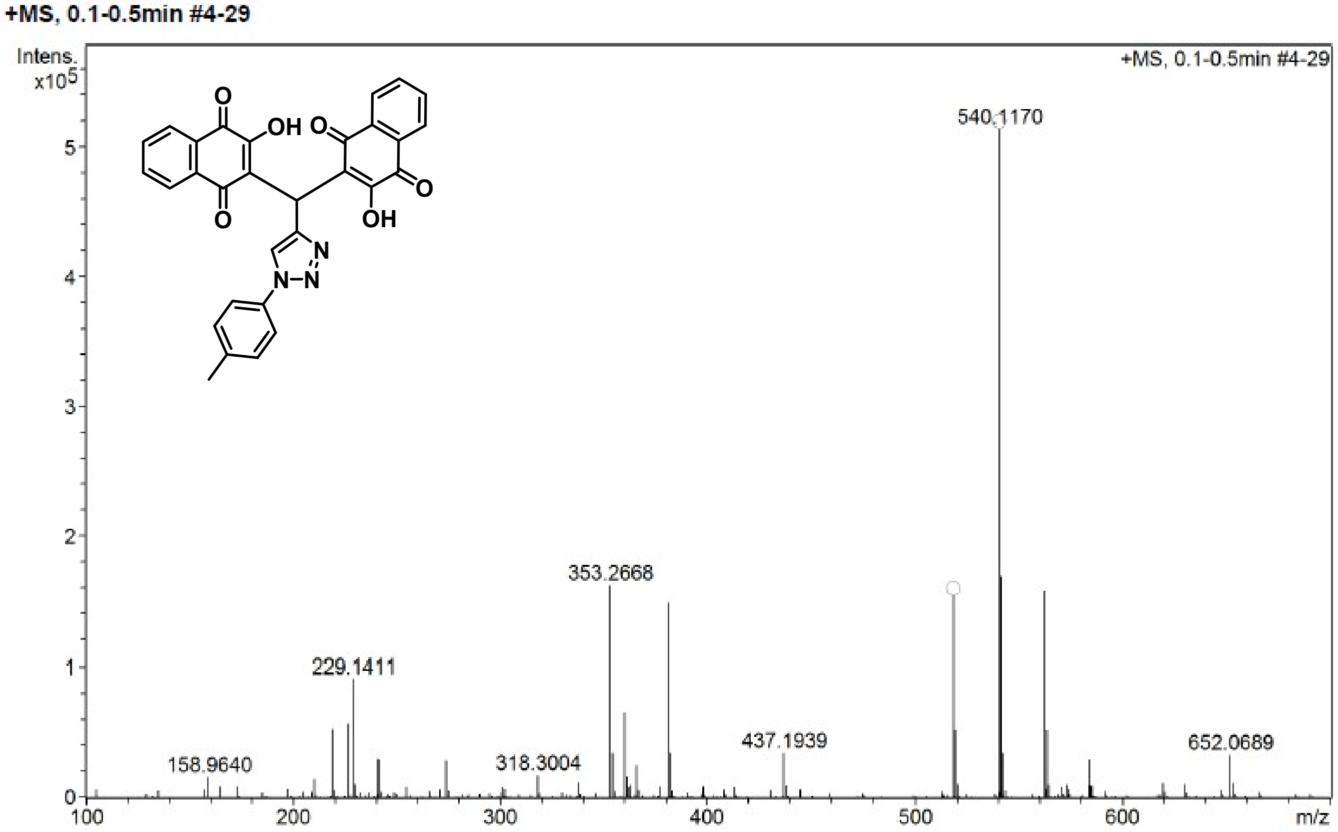

Meas. m/z	#	Ion Formula	m/z	err [ppm]	Mean err [ppm]	rdb	N-Rule	e⁻ Conf	mSigma	Std I	Std Mean m/z	Std I VarNorm	Std m/z Diff	Std Comb Dev
518.135101	1	C30H20N3O6	518.134662	-0.8	-0.9	22.5	ok	even	2.1	3.1	n.a.	n.a.	n.a.	n.a.
	2	C31H16N7O2	518.135999	1.7	1.4	27.5	ok	even	13.8	19.7	n.a.	n.a.	n.a.	n.a.
540.116959	1	C30H19N3NaO6	540.116606	-0.7	-0.4	22.5	ok	even	5.2	8.0	n.a.	n.a.	n.a.	n.a.
	2	C31H15N7NaO2	540.117943	1.8	1.9	27.5	ok	even	16.9	24.6	n.a.	n.a.	n.a.	n.a.

Figure S68. HRMS spectrum of **3y.**

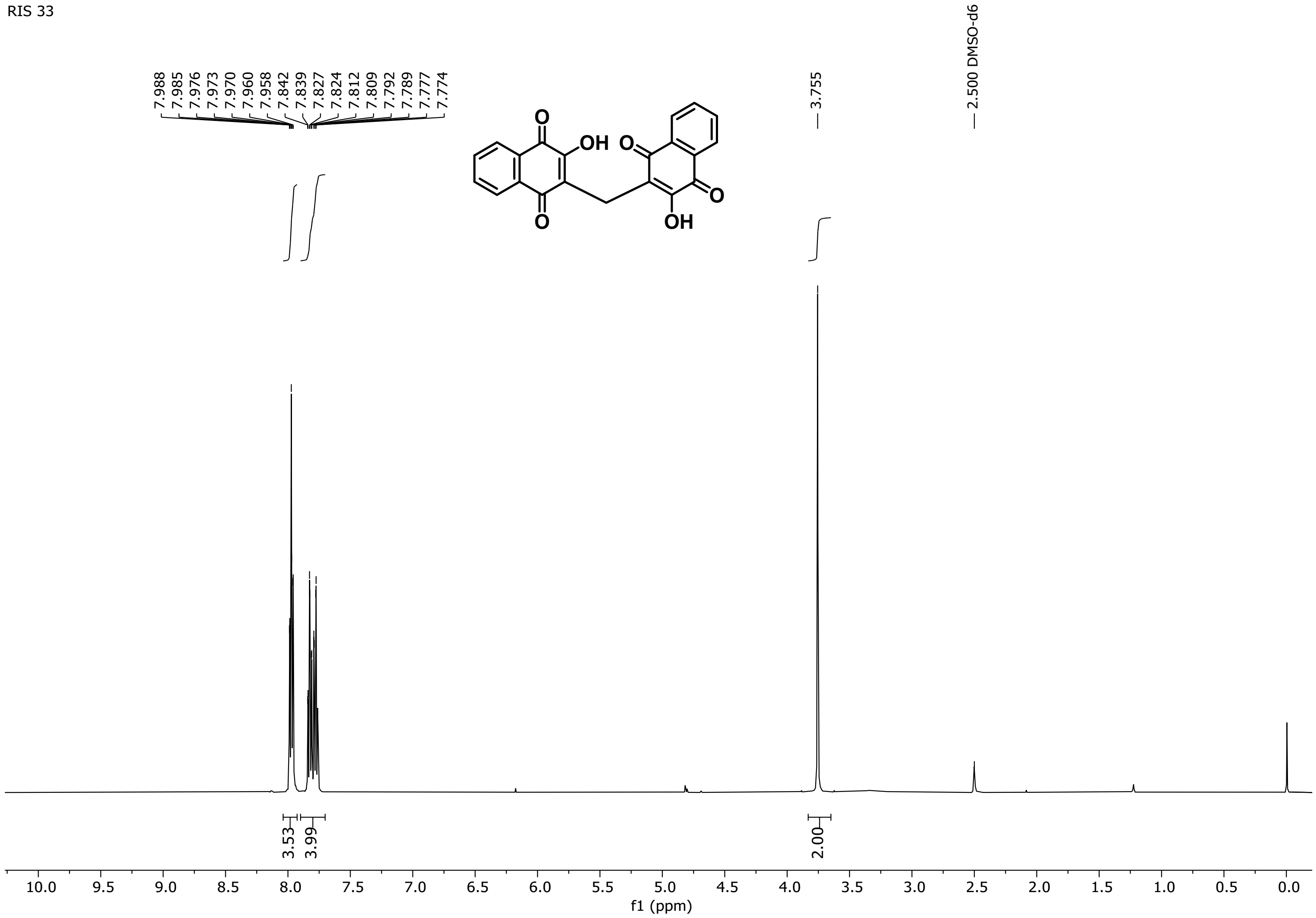

Figure S69. ^{1}H NMR spectrum of **3z** (500 MHz, DMSO-d_6).

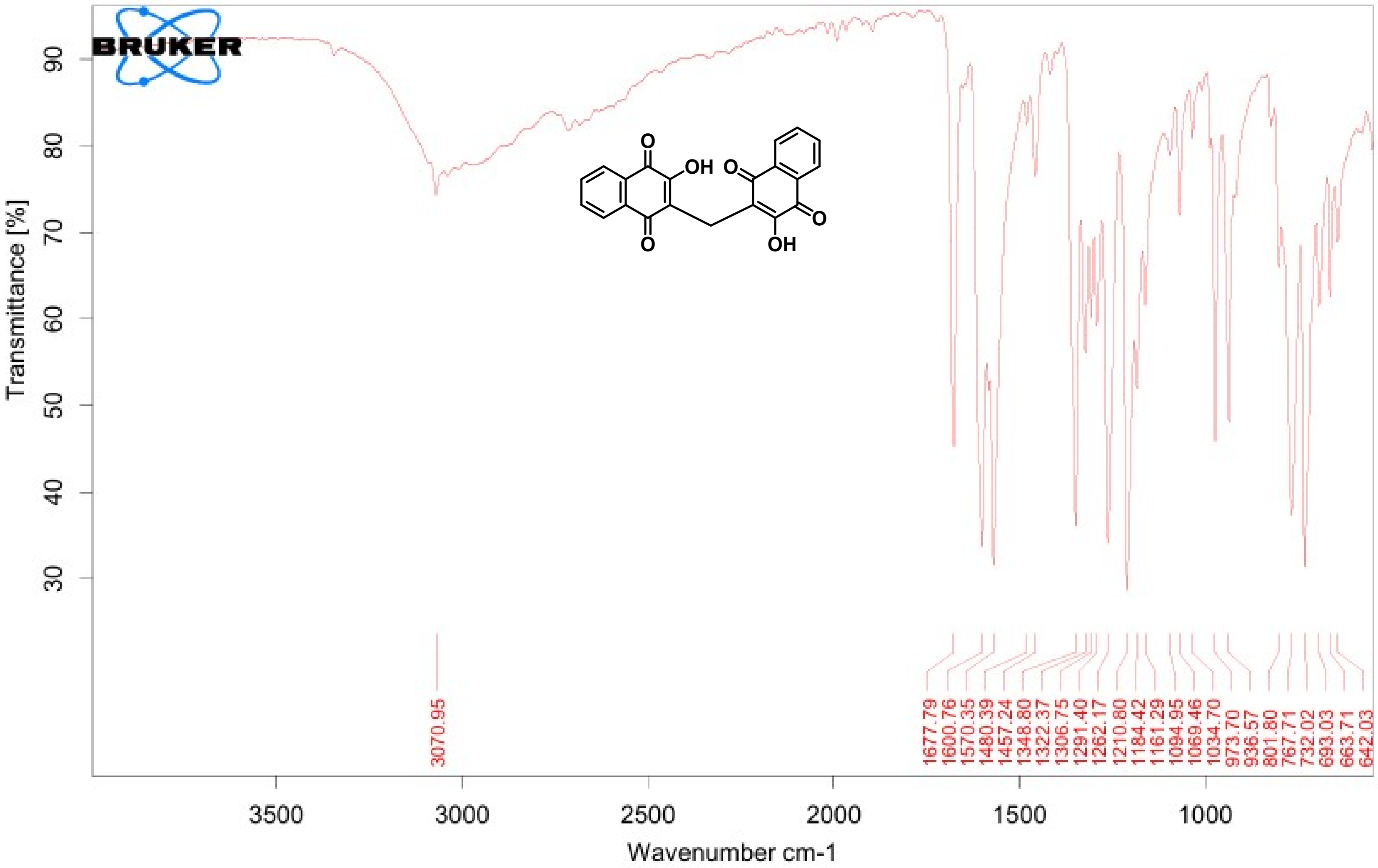

Figure S70. FT-IR spectrum of **3z.**
